# Persistent functional and taxonomic groups dominate an 8,000-year sedimentary sequence from Lake Cadagno, Switzerland

**DOI:** 10.3389/fmicb.2025.1504355

**Published:** 2025-02-03

**Authors:** Paula Rodriguez, Jasmine S. Berg, Longhui Deng, Hendrik Vogel, Michal Okoniewski, Mark A. Lever, Cara Magnabosco

**Affiliations:** ^1^Department of Earth and Planetary Sciences, ETH Zurich, Zurich, Switzerland; ^2^Faculty of Geosciences and Environment, Université de Lausanne, Lausanne, Switzerland; ^3^Institute for Biogeochemistry and Pollutant Dynamics, ETH Zurich, Zurich, Switzerland; ^4^School of Oceanography, Shanghai Jiao Tong University, Shanghai, China; ^5^Oeschger Centre for Climate Change Research, Institute of Geological Sciences, University of Bern, Bern, Switzerland; ^6^ID Scientific IT Services, ETH Zurich, Zurich, Switzerland; ^7^College of Natural Sciences, Marine Science Institute, University of Texas at Austin, Austin, TX, United States

**Keywords:** deep lacustrine sediments, functional potential, microbial communities, biogeochemical cycling, metagenomics

## Abstract

Most of our knowledge of deep sedimentary life comes from marine environments; however, despite their relatively small volume, lacustrine sediments constitute one of the largest global carbon sinks and their deep sediments are largely unexplored. Here, we reconstruct the microbial functional and taxonomic composition of an 8,000-year Holocene sedimentary succession from meromictic Lake Cadagno (Switzerland) using shotgun metagenomics and 16S rRNA gene amplicon sequencing. While younger sediments (<1,000 years) are dominated by typical anaerobic surface sedimentary bacterial taxa (*Deltaproteobacteria, Acidobacteria*, and *Firmicutes)*, older layers with lower organic matter concentrations and reduced terminal electron acceptor availability are dominated by taxa previously identified as “persistent populations” within deep anoxic marine sediments (*Candidatus* Bathyarchaeia, *Chloroflexi*, and *Atribacteria*). Despite these dramatic changes in taxonomic community composition and sediment geochemistry throughout the sediment core, higher-order functional categories and metabolic marker gene abundances remain relatively consistent and indicate a microbial community capable of carbon fixation, fermentation, dissimilatory sulfate reduction and dissimilatory nitrate reduction to ammonium. As the conservation of these metabolic pathways through changes in microbial community compositions helps preserve the metabolic pathway connectivity required for nutrient cycling, we hypothesize that the persistence of these functional groups helps enable the Lake Cadagno sedimentary communities persist amidst changing environmental conditions.

## Introduction

Subsurface marine and lacustrine sediments cover approximately 70% of Earth's surface (Oni et al., 2015; Hoshino et al., 2020) and constitute the largest global organic carbon reservoirs (Barber et al., 2017). This substantial habitat hosts a significant fraction of the world's bacterial and archaeal populations (Kallmeyer et al., 2012) and exhibits much slower metabolic rates than their surface sedimentary counterparts (Jorgensen and Marshall, 2016). This is in part due to the low oxygen supply, which often leads to anoxic conditions that promote the preservation of organic matter and environmental signatures in the sediment record (Jessen et al., 2017). Lacustrine sediments are often regarded as time capsules for paleo-reconstructions, as sediment deposition happens continuously and at higher rates, and experiences less reworking, compared to marine sediments (Zolitschka and Enters, 2009). The resulting high-resolution temporal records of environmental change (Zolitschka and Enters, 2009) make lake sediments ideal settings to study the interplay between long-term environmental changes in lake water sheds and biogeochemical processes which slowly alter environmental records after burial (Ariztegui et al., 2015). Despite their unique features, the effects of sediment burial on the community assembly, function, and diversity in deep lacustrine sediments are poorly understood, especially when compared to those of deep marine sediments (Ariztegui et al., 2015). Studies on microbial assemblages in deep lacustrine sediments are thus essential for understanding how the environmental features of lake ecosystems shape sediment microbial communities (Vuillemin et al., 2018).

On a global scale, organic carbon content is one of the main environmental parameters correlated with microbial biogeography patterns in anoxic sediments (Hoshino et al., 2020). In places with high sedimentation rates (e.g., Lake Cadagno, the site of this study), microorganisms living in the water-sediment interface rapidly deplete high-energy terminal electron acceptors (TEAs) such as O_2_ and nitrate and degrade the chemically most reactive organic matter pools (Orsi, 2018; D'Hondt et al., 2004; Berg et al., 2022). Below this zone, the utilization of terminal electron acceptors in catabolic reactions generates a vertical gradient in dominant respiration reactions (D'Hondt et al., 2004). Following the same trend, organic matter compositions shift toward increasingly degradation-resistant (refractory) species with sediment age and depth, thus limiting the electron donor pool available for microbial oxidation (Gajendra et al., 2023; Deng et al., 2020; Han et al., 2022).

As a general trend, the shift in sediment communities from freshly deposited sediments (Morono et al., 2020; Hoshino et al., 2020) to older “deeper” sediment communities is accompanied by a steep drop in microbial abundance (Chen et al., 2017; Hoshino et al., 2020). It is proposed that despite a reduction in cell numbers with increasing sediment depth, microorganisms continue to drive elemental cycling in deep sediments (Varliero et al., 2019; D'Hondt et al., 2004) and presumably utilize refractory organic matter (Marshall et al., 2019; Hoshino et al., 2020; Hubert et al., 2009). Under these conditions, it is hypothesized that a fraction of the surface microbial populations becomes predominant with sediment depth (Petro et al., 2017), and these are frequently referred to as “persistent sediment populations” (Starnawski et al., 2017). In anoxic marine sediments, these are commonly representatives from the groups *Atribacteria, Candidatus* Bathyarchaeia (also referred to as *Candidatus* Bathyarchaeota), and *Chloroflexi* (Hoshino et al., 2020; Zhou et al., 2018; Lloyd et al., 2013).

Here, we investigated how genome-inferred microbial community composition and metabolic potential relate to the environmental history and organic matter sources of lacustrine sediments from Lake Cadagno, an Alpine meromictic lake in Switzerland with anoxic, sulfidic bottom waters. The high-resolution limnological record of Lake Cadagno reflects these environmental changes, providing an ideal setting to study the co-evolution of anoxic lacustrine sediments and their microbial communities. Our study encompasses deposition throughout the Holocene and after the onset of meromictic conditions in the lake. An initial biological analysis of the sedimentary sequence revealed a weak (R^2^ = 0.35) correlation between total organic carbon and prokaryotic 16S rRNA gene abundances (Berg et al., 2022); however, the functional and population-level diversity of the microbial communities within these sediments was not investigated. In this study, we combine shotgun metagenomic and 16S rRNA gene sequencing data to investigate the functional and taxonomic microbial community composition across an 8,000-year lacustrine sedimentary covering the period after the establishment of water column stratification and bottom water euxinia in Lake Cadagno.

## Methods

### Sample collection and study site

Lake Cadagno is a meromictic lake located in the Swiss Alps at 1,921 meters above sea level. The lake basin was formed through glacial erosion into bedrock during the Last Glacial Period with lake conditions being established after the retreat of the glacier from the basin ~12,000 years ago (Wirth et al., 2013). Since its formation and throughout its history it has undergone water column redox transitions that are reflected in the sediment record (Wirth et al., 2013). Today, the water column of the lake is stratified into an oxic epilimnion and an anoxic and sulfidic hypolimnion separated by a 0.5–1.0 m thick chemocline populated by purple sulfur bacteria (PSB) and green sulfur bacteria (GSB) (Philippi et al., 2021). The bottom layer of the water column exhibits a high sulfate concentration (~2 mM) relative to other freshwater systems, and surface sediments are similarly enriched in sulfate (up to 1.5 mM) which is rapidly depleted (<0.05 mM) within the first 20 cm below the lake floor (cmblf) (Berg et al., 2022). In the summer of 2019, a 10 m sediment core was retrieved and an initial 16S rRNA gene-based microbial characterization was performed (Berg et al., 2022). In our current study, a new set of sediment samples from this core were independently analyzed for 16S rRNA gene and shotgun metagenomic analyses. A detailed description of the coring, sampling strategy and geochemistry can be found in Berg et al. (2022). In brief, three sediment cores from Lake Cadagno were collected at a water depth of 21 m (46.55060 N and 8.71201 E) using an UWITEC coring platform (Uwitec, TA) and subsampled to biomolecular analytical standards. After retrieval, biomolecular samples were flash-frozen in liquid N_2_ and stored at −80°C until the DNA extractions described below.

### Sample selection, DNA extraction, and metagenomic sequencing

DNA samples from 13 sediment depths between 0 and 738 cmblf were selected for DNA extraction and metagenomic sequencing (Table 1). Sample selection was based on lithostratigraphic and geochemical parameters to make sure the environmental variability of the sedimentary sequence was well represented. The core exhibits layers of organic matter-rich sediments of lacustrine origin and turbidite layers originating from mass-movement events that vary in porewater $\left(\text{M}{\text{n}}^{2+},\text{ F}{\text{e}}^{2+},\text{ S}{\text{O}}_{4}^{2-},\text{ N}{\text{O}}_{3}^{-},\text{ P}{\text{O}}_{4}^{3-},\text{ N}{\text{H}}_{4}^{+}\right)$ and solid phase (S^0^, TOC, C:N ratio, δ^13^C-TOC) chemistry (Berg et al., 2022) (Table 1). The 13 samples cover the depositional history of the lake in the last 8,000 years after the onset of water column stratification in the lake during the Holocene, as indicated by age-depth modeling based on ^14^C radiocarbon dating (Berg et al., 2022; Wirth et al., 2013).

**Table 1:** General information and geochemical data (Berg et al., 2022) for the 13 sediment samples analyzed this study.

**Sample depth (cm)**	**Sediment type**	**Age mean (Cal BP)**	**TOC (%)**	**δ^13^C-TOC**	**CH_4_ (mmoI/I)**	**S^0^ (μml/g dry sed)**	**C:N**	**${\text{SO}}_{4}^{2-}$(μmoI/I)**	**${\text{NO}}_{3}^{-}$(μmol/l)**	**${\text{NH}}_{4}^{+}$ (μmol/L)**
3	Pelagic	−17.3	17.3	−32.02	0.35	211.4	11.44	1,347.4	0	200
40	Turbidite	221.8	1.7	−27.15	2.23	6.29	13.41	0.09	0	-
153	Turbidite	1,026.6	1.3	−28.19	3.16	1.06	10.24	13.55	0.56	982
187	Turbidite	1,270.1	2.81	−28	4.49	6.75	13.23	12.8	0.26	793
213	Pelagic	1,456.3	2.3	−28.44	4.20	0.36	14.11	24.52	0.32	859
233	Turbidite	1,586.7	2.08	−28.04	4.27	0.57	13.67	51.41	0.37	878
283	Pelagic	1,741.7	1.43	−27.5	2.73	0.6	12.15	26.51	0.88	651
382	Pelagic	2,504.3	1.14	−28.47	2.12	0.34	9.93	10.62	0.19	470
532	Turbidite	3,472.5	1.97	−27.64	1.65	0.06	16.57	11.54	1.66	453
566	Pelagic	3,725.5	4.46	−30.04	4.34	0.28	13.3	-	-	-
582	Turbidite	4,179.5	4.29	−29.93	2.76	0.09	15.02	1.98	0.55	547
693	Turbidite	7,019.8	7.29	−34.67	1.41	0.07	13.1	5.31	0.68	348
738	Turbidite	8,268.6	4.66	−33.8	1.27	8.63	13.97	3.83	0.61	279

Additional geochemical parameters can be found in Supplementary Table 1. Cal BP, calibrated ^14^C ages expressed in years before present; TOC, Total Organic Carbon, C:N, carbon-nitrogen ratio. Remineralization zones with peaks in phosphate ($PO_{4}^{3-}$), ammonium ($NH_{4}^{+}$), iron (Fe^2+^) and manganese (Mn^2+^) are highlighted in lime green. For further details on geochemical data, refer to Supplementary Table 1 (extended version).

DNA was extracted from three 0.2 g sediment aliquots per sample depth using a modular DNA extraction protocol (Lever et al., 2015) to maximize the DNA yield in deep, old sediments. Briefly, the aliquots were transferred to 2 mL beat-beating tubes and soaked with 10 mM sodium hexametaphosphate solution, and lysis solution I (30 mM Tris-HCl, 30 mM EDTA, 800 mM guanidium hydrochloride, and 0.5% Triton X-100) and then placed in a vortex genie and shaken at maximum speed for 30 s. The samples were then purified using lysis solution II (2% CTAB and 0.1% PVPP) and 24:1 chloroform isoamyl alcohol to remove humic and fluvic acids, residual proteins, and lipids. DNA precipitation was performed using linear polyacrylamide and 70% ethanol. DNA extracts were then purified using magnetic beads (AMPure XP beads 1.0*x*) followed by a final purification and concentration step using the commercial kit ReliaPrep^TM^ DNA Cleanup and Concentration System (Promega, WI, United States).

After extraction and clean-up, the final DNA concentration of samples ranged between 2.44 ng/μL and 17.15 ng/μL (Supplementary Table 1). Samples were normalized to the minimum concentration of 2 ng/μL, which allowed us to perform metagenomic sequencing using the standard NEBnext Ultra II DNA library preparation kit which uses DNA fragmentation by sonication (New England Biolabs, Massachusetts, United States). The NEBnext Ultra II DNA for Illumina library preparation kit requires an initial DNA input of 50 ng and a three-cycle PCR amplification step. This reduced number of PCR cycles minimizes the impact of PCR on gene duplication rates, unequal amplification of genes, and sequencing artifacts (Rochette et al., 2023). Paired-end high-throughput sequencing was performed on a NovSeq 6000 Sequencing System (Illumina, San Diego, CA, USA) at the Functional Genomics Center Zurich (Zurich, Switzerland).

### Metagenomic assembly

Sequences were quality-filtered and trimmed using Trimmomatic v.0.35 (Bolger et al., 2014) with the parameters SLIDING WINDOW:4:15 MINLEN:36 and assembled using Spades 3.14.1 (metaSPAdes.py) (Nurk et al., 2017) with k-mer sizes 21, 33,55. Contigs larger than 1,000 bp were retained for further analyses and protein-coding regions were predicted using Prodigal v.2.6.3 (Hyatt et al., 2010). Predicted proteins from all samples were clustered at a 90% amino acid sequence identity threshold using CD-HIT v.4.6.8 (Li and Godzik, 2006). The representative sequence for each predicted protein cluster was functionally and taxonomically annotated as described below. The taxonomic and functional annotation of each predicted protein cluster's representative protein sequence was then assigned to all sequences within the protein cluster.

### Taxonomic and functional gene annotation

Functional annotation of protein clusters within the Clusters of Orthologous Groups (COG) broad metabolic categories was made using eggNOG mapper v.2.1.6 with a likelihood threshold of 1e−5. The functional annotation of METABOLIC-derived marker genes (Zhou et al., 2022) for carbon, sulfur, and nitrogen biogeochemical cycling was performed using HMMER v.3.3.2 (Finn et al., 2011, e-value threshold of 1e−15) and the identity of the marker genes was confirmed using BLAST+ v.2.9.0 (e-value threshold of 1e−30, Camacho et al., 2009) against the NCBI-nr database (February 2024). The taxonomic annotation of the RpS3 marker genes was confirmed using BLAST+ v.2.9.0 (e-value threshold of 1e−30; Camacho et al., 2009) against the NCBI-nr database (January and June 2023). The taxonomy was assigned to the last common ancestor using a consensus vote approach with the top 3 hits of the previously described BLAST*p* search. The functional identities of marker genes involved in nitrogen cycling (*hao, narG, nifD, nifH, nifK, norB*) and sulfur oxidation (*soxB*) genes were further confirmed, and the taxonomic identities of the sequences were assigned through phylogenetic placement. Briefly, a “reference” phylogenetic tree for each marker gene was constructed by randomly sampling 100 protein sequences from the original METABOLIC marker gene database using seqtk v.1.3. Protein sequence alignments of METABOLIC marker gene and Lake Cadagno sequences were computed using MUSCLE v.3.8.31 (Edgar, 2004) and *de-novo* phylogenetic trees were calculated using FastTree v.2.1.11 (Price et al., 2010) using the Le and Gascuel (LG) model (Le et al., 2008). The placement of Lake Cadagno sequences within these marker gene trees was used for marker gene taxonomic identification.

### Functional annotation and analysis of carbohydrate cycling genes

The complete set of protein sequences clustered at 90% amino-acid identity was annotated using the eggNOG mapper v.2.1.6, with a likelihood threshold of 1e−5. Protein clusters within each of the Clusters of Orthologous Groups (COGs) were subsampled for a beta-diversity analysis using complete linkage hierarchical clustering. Based on these results, the category “G” (Carbohydrate transport and metabolism) was selected for an enrichment analysis using the Maaslin2 (Mallick et al., 2021). The enrichment analysis was performed on samples where persistent Amplicon Sequencing Variants (ASVs) from *Ca*. Bathyarchaeia, *Atribacteria*, and *Chloroflexi* constitute more than 10% of the total abundance in the microbial community (samples below 40 cm). Proteins showing significant correlations and anticorrelations to sample depth (q-value <0.05) were further annotated using BLAST (e-value threshold of 1e−30; Camacho et al., 2009) against the NCBI-nr database (April 2024).

Finally, to identify potential cellulases in the complete metagenomic dataset, protein sequences identified as cellulases based on the eggNOG mapper annotation were selected. The taxonomic and functional annotations of these proteins were further confirmed using BLAST (e-value threshold of 1e−30; Camacho et al., 2009) against the NCBI database (April 2024).

### Metabolic pathway completeness assessment

Protein sequences clustered at 90% amino-acid identity were annotated using eggNOG mapper v.2.1.6 with a likelihood threshold of 1e−5. Protein clusters annotated within any of the KEGG categories were further annotated using GhostKOALA (Kanehisa et al., 2016). The resulting output file was used as input for KEGGDecoder (Graham et al., 2018) to generate a functional heatmap illustrating the completeness of the metabolic pathways across different sediment depths for the entire metagenomic dataset. The completeness of the metabolic pathways at any given depth is expressed using scores that range from 0.0 (non-present pathway) to 1.0 (indicating a fully present metabolic pathway).

### Phylogenetic analysis of *Candidatus* Bathyarchaeia

A collection of RpS3 protein sequences was obtained from genomes assigned to *Ca*. Bathyarchaeia in GenBank (Benson et al., 2017), reported in the *Ca*. Bathyarchaeia phylogenetic tree of a previous study (Zhou et al., 2018), and identified within the Lake Cadagno metagenomic dataset. A protein sequence alignment was made using MUSCLE (Edgar, 2004). To improve the topological correspondence of our *Ca*. Bathyarchaeia RpS3 tree and previously reported *Ca*. Bathyarchaeia phylogenetic trees (Zhou et al., 2018; Hou et al., 2023), a guide tree was calculated using FastTree v.2.1.11 (Price et al., 2010) using the LG substitution model (Le et al., 2008). A maximum likelihood phylogenetic tree was then calculated using the LG protein substitution model (Le et al., 2008), gamma likelihood optimization and guide tree in RAxML v.8.2.12 (Stamatakis, 2014). Bootstrap analysis with 1,000 replicates was performed to assess the robustness of the tree topology.

### 16S rRNA targeted gene sequencing

The V4–V5 hypervariable regions of the 16S rRNA gene were amplified using the universal primer pair 515 F (5′-GTG YCA GCM GCC GCG GTA A-3′) and 926 R (5′-CCG YCA ATT YMT TTR AGT TT-3′) (Quince et al., 2011; Parada et al., 2016). This primer pair covers ~500 bp in the V4–V5 hypervariable regions of the bacterial and archaeal 16S rRNA gene and is different from the universal, bacteria-specific and archaea-specific primer pairs used by Berg et al., 2022 [S-D-Bact-0341-b-S-17/S-D-Bact-0785-a-A-21 (Herlemann et al., 2011) and -D-Arch-0519-a-A-19 (Sørensen and Teske, 2006)/967Rmod (Cadillo-Quiroz et al., 2006)]. Amplicon libraries were prepared using a single-step PCR on the C1000 Touch Thermal Cycler (BioRad, Hercules, California, United States). Samples were run in triplicates, with a reaction volume of 25 μL including 1.0 μL template DNA, 0.5 μL forward primer (10 μM) and reverse primer (10 μM), 10 μL of PlatinumTM Hot Start PCR 2X Master Mix (Thermo Fisher Scientific, Waltham, Massachusetts, United States), and 13 μL of PCR-grade water. The PCR program was run as follows: initial denaturation at 95°C for 3 min, followed by 30 cycles of denaturation at 95°C for 35 s, annealing at 50°C for 45 s, elongation at 68°C for 90 s and a final elongation step at 68°C for 5 min. The DNA amplicons were purified using AMPure XP magnetic beads (Beckman Coulter, Brea, California, United States) at a concentration of 0.8*x*. After purification, the DNA amplicon concentrations were measured using a Qubit and diluted to create a pool with a final concentration of 4 nM. The quality of the 16S rRNA gene library was determined using the D1000 ScreenTape on the Agilent 4150 TapeStation (Agilent Technologies, Santa Clara, California, United States). Paired-end sequencing was performed on a MiSeq sequencer (Illumina Inc., San Diego, California, United States) using the MiSeq Reagent Kit v2 Nano (500 cycles) (Illumina).

### 16S rRNA gene sequencing data analysis

The quality of the 16S rRNA gene sequences was assessed using FastQC (Andrews, 2010). Data analysis was done using the standard the Qiime2 workflow (Bolyen et al., 2019). In brief, the first six nucleotides of the forward reads and 22 of the reverse reads were trimmed using the –p-trim-left parameter in the denoise-paired function of DADA2 (Callahan et al., 2016). Forward and reverse reads were truncated at 200 and 190 bp, respectively, with the –p-trunc-len function. The 16S rRNA gene feature table was generated and summarized using the qiime feature-table summarize function in Qiime2 (Bolyen et al., 2019). Amplicon sequencing variants (ASVs) related identified within the negative control samples were removed from all samples and excluded from all downstream analyses. Remaining ASVs were annotated using the Silva (Quast et al., 2013) nr database version 138.1.

The OTU table was rarefied to the minimum sample read count using the rrarefy function in the Vegan package (Oksanen et al., 2024) in RStudio to normalize sequencing depth across samples. Richness, defined as the number of unique taxa per sample, was calculated by counting non-zero taxa in the rarefied and non-rarefied data. The Shannon diversity index was calculated for each sample using the diversity function from the Vegan R package.

### 16S rRNA gene quantification

Abundances of prokaryotic 16S rRNA genes were quantified on a LightCycler 480 II (Roche Life Science, Penzberg, Germany) at ETH Zurich's Genetic Diversity Center (Switzerland) using the universal primer pair 515 F (5′- GTG YCA GCM GCC GCG GTA A−3′) and 926 R (5′- CCG YCA ATT YMT TTR AGT TT−3′) (Quince et al., 2011; Parada et al., 2016). Plasmids of 16S rRNA genes from *Thermoplasma acidophilum*-affiliated archaea and *Holophaga foetida* were used as standards as described in Han et al. (2020). The samples were run in duplicates with a reaction volume of 10 μL including 2 μL of DNA template, 1 μL of water, 1 μL of BSA, 0.5 μL of forward primer (10 μM) and 0.5 μL of reverse primer (10 μM), and 5 μL SYBR green (SsoFast™ EvaGreen^®^ Supermix with Low ROX 2x) (BioRad, Hercules, California, United States). The quantitative PCR (qPCR) reaction was run as follows: activation of the DNA polymerase at 95°C for 5 min, followed by 35 cycles of initial denaturation at 95°C for 10 s, annealing at 50°C for 30 s, extension at 68°C for 15 s and a melting curve (95°C for 15 s and 55°C for 1 min). The standard curve and melt curve for these reactions are provided in Supplementary Figures 1, 2.

## Results

### Microbial community composition and abundance in Lake Cadagno sediments

The taxonomic composition of the microbial communities from Lake Cadagno sediments was assessed using the 16S rRNA gene and the ribosomal protein S3 (RpS3) predicted protein clusters at 90% amino acid sequence identity (RpS3_90%_) from targeted and shotgun metagenomic datasets, respectively. At a coarse taxonomic level, the taxonomic profiles reported in this study based on targeted and shotgun metagenomic sequencing data match previously published data, which shows a clear transition from surface sediments dominated by bacteria from groups like *Proteobacteria*, to deeper sediments dominated by *Ca*. Bathyarchaeia and other groups such as *Planctomycetes, Chloroflexi* and *Atribacteria* (Berg et al., 2022).

**Figure 1:**
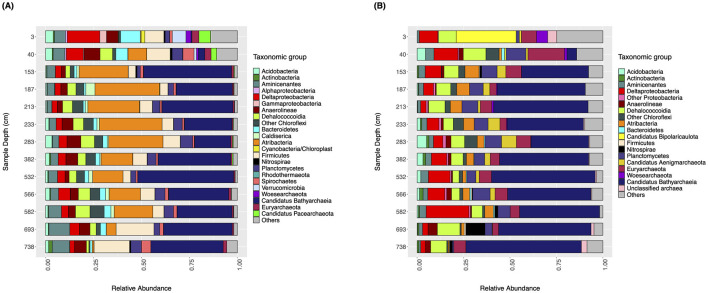
Taxonomic characterization of microbial communities from Lake Cadagno with sediment depth. The relative abundance (x-axis) of bacteria and archaea (bar colors) with respect to sediment depth (y-axis) is shown **(A)** for 16S rRNA gene sequences and **(B)** for ribosomal protein S3 (RpS3_90%_) sequence.

The microbial community composition profiles based on 16S rRNA amplicon and RpS3_90%_ datasets are generally consistent with the exception of the unique presence of *Candidatus* Atribacteria (formerly OP9) at relatively high abundances (12–33%) in the 16S rRNA gene dataset from 153 cmblf and 582 cmblf (Figure 1, Supplementary File 2) and the dominance (33%) of *Candidatus* Acetothermia (Bipolaricaulota) within the youngest (3 cmblf) sediment sample of the RpS3_90%_ dataset (Figure 1). Both datasets reveal bacteria-dominated communities composed of *Alphaproteobacteria, Gammaproteobacteria*, and *Deltaproteobacteria* with minor populations of *Euryarchaeota* in the two youngest sediment samples (3 cmblf and 40 cmblf). In deeper and older samples (>225 years, >40 cmblf), the bacteria-dominated communities are replaced by *Candidatus* Bathyarchaeia-dominated communities (Figure 1). 16S rRNA gene and RpS3_90%_ sequences related to *Ca*. Bathyarchaeia, *Ca. Aminicenantes, Deltaproteobacteria, Chloroflexi, Atribacteria, and Planctomycetes* are identified in all samples. Finally, higher alpha-diversity metrics [richness (*S*), Shannon-Wiener Index (*H'*)] are observed within the 16S rRNA gene dataset relative to the RpS3_90%_ dataset (Table 2). This discrepancy in dataset-derived *S* and *H*' can be explained by the differences in both the sequencing (targeted vs. untargeted) and bioinformatic approach (Amplicon Sequencing Variant vs. RpS3_90%_).

**Table 2:** General sequencing information and diversity metrics per sample depth for the 16S rRNA gene and shotgun metagenomic sequencing datasets.

	**16S rRNA targeted sequencing—General metrics**					**Metagenomic sequencing and RpS3**_**90%**_ **profile—General metrics**		
**Sample depth (cm)**	**Number of reads per sample**	**Richness (ASVs)**	**Shannon-Wiener Diversity Index (** ***H**′* **)**	**Richness – rarefied table (ASVs)**	**Shannon-Wiener Diversity Index—rarefied table (H**′**)**	**Number of reads per sample (millions)**	**Richness (RpS3**_90%_ **)**	**Shannon-Wiener Diversity Index (** ***H**′* **)**
3	34,343	1,267	6.16	1,138	1.05	52.5	71	3.24
40	39,158	1,407	6.07	1,151	1.04	65.5	109	4.31
153	12,879	230	3.11	230	0.29	49.2	85	3.41
187	57,496	579	3.66	467	0.49	50.6	64	3.02
213	61,388	522	3.36	423	0.45	44.7	73	2.93
233	56,713	717	4.09	564	0.58	54.5	101	3.57
283	59,019	821	4.57	642	0.65	53.9	130	4.15
382	65,587	732	4.12	537	0.56	51.7	72	3.36
532	65,302	512	3.42	397	0.43	38.9	61	2.83
566	39,951	465	4.16	416	0.47	38.4	64	3.35
582	59,423	559	4.05	445	0.48	40.9	41	2.89
693	62,189	480	4.09	392	0.44	63.7	73	3.25
738	39,709	380	4.19	343	0.4	47.9	54	3.12

The community composition profiles and diversity metrics calculations are based on 16S rRNA amplicon sequencing variants (ASVs) and the profile derived from ribosomal protein S3 sequences clustered at 90% amino acid identity (RpS3_90%_), respectively. The richness is expressed as the total number of ASVs or RpS3_90%_ per sample. The alpha diversity metrics for the 16S rRNA gene sequencing dataset (ASVs) presented in the table include calculations performed on both the rarefied and non-rarefied ASV tables. The number of reads per sample is expressed in millions for the shotgun metagenomic sequencing dataset. For further details, refer to Supplementary Table 1 (extended version).

**Figure 2:**
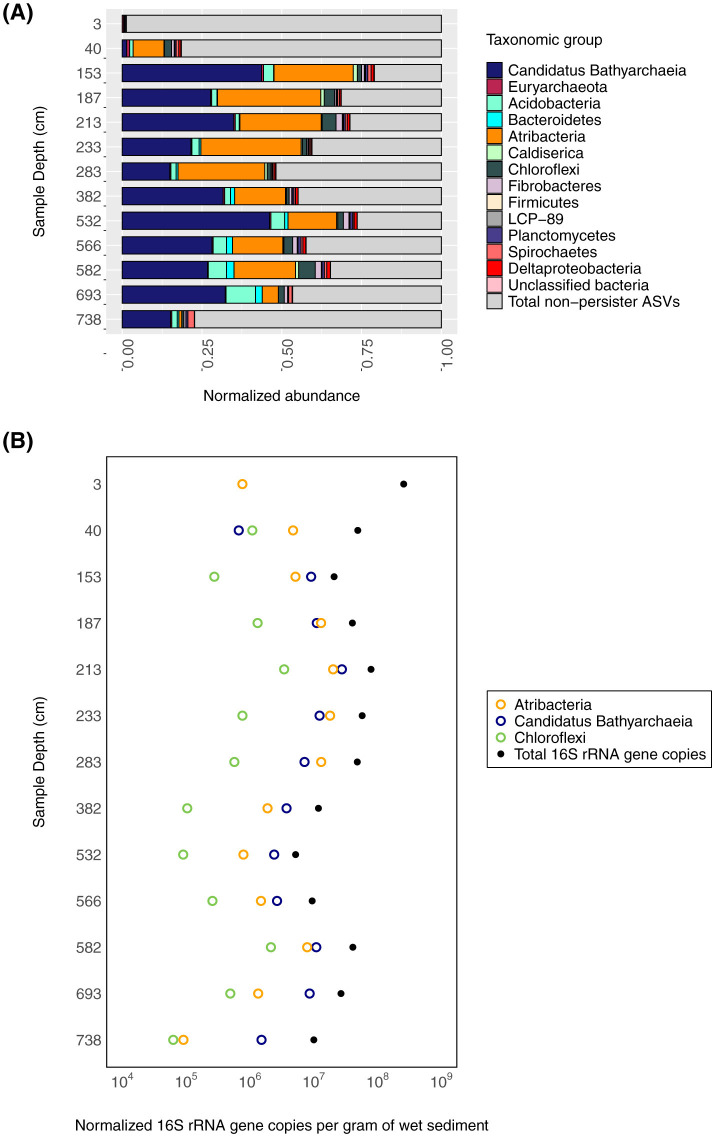
Persistent microbial lineages throughout the sedimentary sequence. **(A)** The relative abundance of persistent (present in more than 10 sediment samples) 16S rRNA ASVs and non-persistent ASVs (gray bars) are shown with respect to sediment depth. **(B)** The qPCR-estimated abundance of bacteria and archaea (black dots) relative to estimated persistent *Ca*. Bathyarchaeia (open blue circles), *Atribacteria* (open orange circles), and *Chloroflexi* (open green circles) ASV abundance (x-axis) with depth (y-axis) is shown.

In the first 153 cm of sediment, total 16S rRNA gene copy numbers decrease by more than an order of magnitude (2 × 10^8^ to 2 × 10^7^ gene copies per g of wet sediment), and below this depth, community abundances range from 5 × 10^6^ to 8 × 10^7^ 16S rRNA gene copies per g of wet sediment (Figure 2B). While almost all samples within the 16S rRNA gene dataset are bacteria-dominated, both bacteria- and archaea-specific abundances vary with depth. Two samples (153 cmblf and 532 cmblf) exhibit nearly equal bacteria-to-archaea ratios as reported by Berg et al. (2022) even though their only shared geochemical traits are similarities in sediment type (turbidite) and phosphate concentrations (30.3 μM) (Table 1).

### Persistent members of the sediment microbial community

In addition to the changes in Lake Cadagno community composition and abundance, persistent taxonomic groups are also observed throughout the sediment sequence. Persistent groups are generally defined as the members of the community that are present throughout the sediment column (Starnawski et al., 2017). In this study, we define as “persistent groups” the Amplicon Sequencing Variants that are present in more than ten sediment samples in our sedimentary sequence. Out of the 6,148 unique Lake Cadagno Amplicon Sequencing Variants (ASVs), 42 ASVs are observed in at least 11 of the 13 Lake Cadagno sediment sample depths. The persistent ASVs represent a small fraction of the surface sediment community at 3 cmblf (<0.01%) but represent between 33% and 67% of each sample's total 16S rRNA gene-based community below 153 cmblf. The most abundant persistent ASVs are related to *Ca*. Bathyarchaeia (16/42), *Atribacteria* (3/42), and *Chloroflexi* (3/42) (Figure 2B). Additionally, persistent ASVs related to *Acidobacteria* (5/42), *Plantomycetes* (2/42), *Deltaproteobacteria* (2/42), *Fibrobacterota* (1/42) and *Bacteroidota* (1/42) were also identified at >2% (SD = 3.51) average abundance below 153 cm. Among these, the most abundant ASV, at an average abundance of 21% of ASVs in sediments below 153 cm, is related to *Ca*. Bathyarchaeia (ASV_ID= Candidatus Bathyarchaeia ASV 1, Supplementary Figure 3).

### *Ca*. Bathyarchaeia profile based on the metagenomic dataset

**Figure 3:**
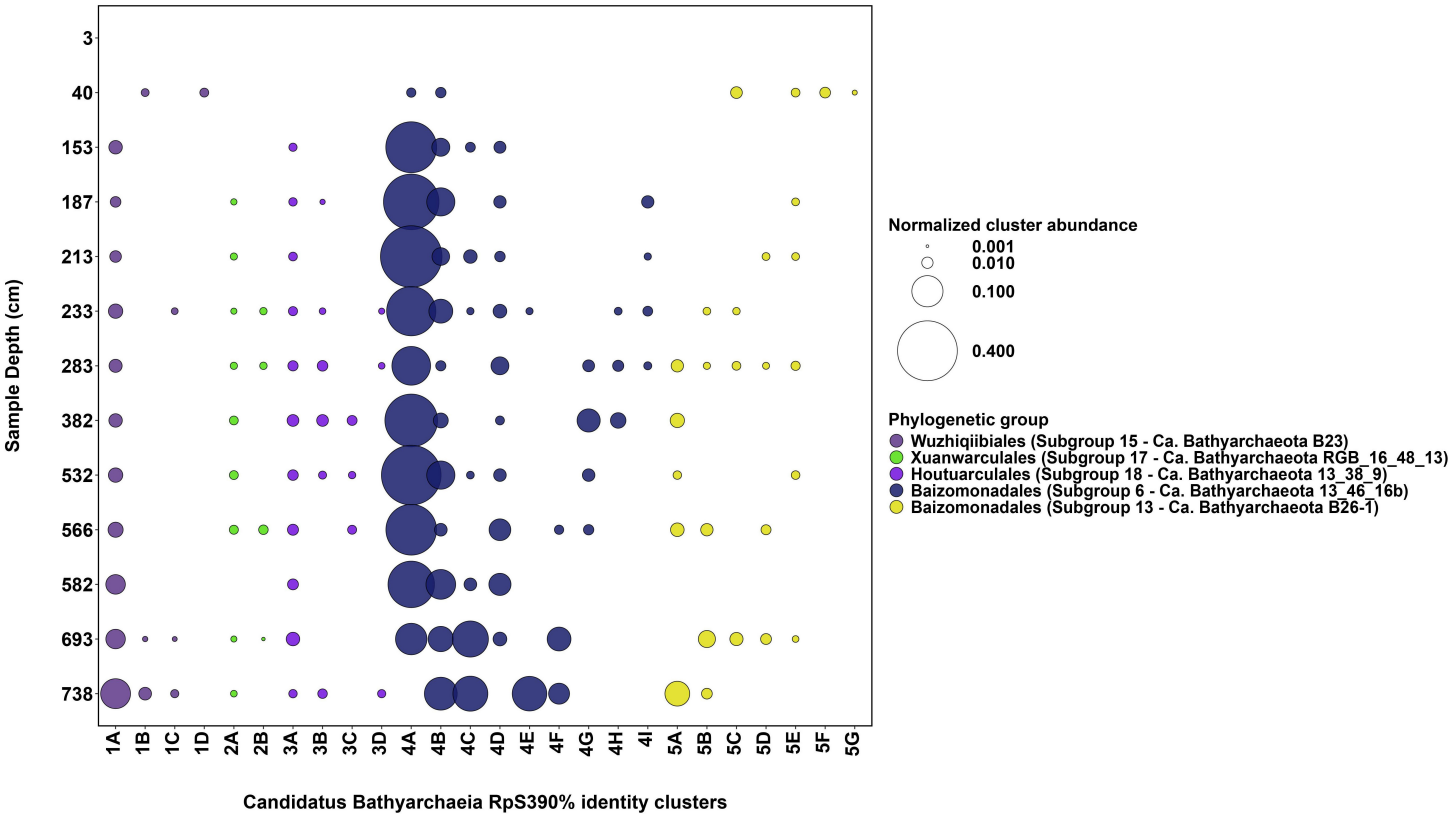
Diversity of *Ca*. Bathyarchaeia subgroups in Lake Cadagno sediments. The normalized RpS3_90%_ cluster abundance (circle size) of the 26 most abundant *Ca*. Bathyarchaeia RpS3_90%_ clusters (x-axis, 1A−5G) per sediment depth (y-axis) is shown. The digits (1–5) associated with each RpS3_90%_ cluster ID reflects each cluster's putative phylogenetic clade assignment (circle color).

Phylogenetic placement of *Ca. Bathyarchaeia* RpS3_90%_ sequences reveals that the Lake Cadagno sediment RpS3 sequence clusters fall within the newly assigned *Ca*. Bathyarchaeia candidate orders “*Baizomonadales*” (*n* = 19; GTDB o__ B26-1; This Study “Cluster 4” and “Cluster 5”), “*Houtuarculales*” (*n* = 4; GTDB o__40CM-2-53-6; This Study “Cluster 6”), “*Wuzhiqibiales*” (*n* = 4; GTDB o__TCS64; This Study “Cluster 1”), and “*Xuanwuarculales*” (*n* = 2; GTDB o__RBG_16_48_13; This Study “Cluster 2”). The majority of these Lake Cadagno-derived *Ca*. Bathyarchaeia RpS3 sequences are more closely related to other representatives within our sediments, than to other *Ca*. Bathyarchaeia subgroups reported in previous studies (Zhou et al., 2018; Hou et al., 2023). The most abundant persistent *Ca*. Bathyarchaeia ASV (ASV_ID= *Candidatus* Bathyarchaeia ASV 1, Supplementary Figure 3) is significantly correlated to *Ca*. Bathyarchaeia RpS3_90%_ sequence “Cluster 4A” (Figure 3) (*r* = 0.81, corrected *p*-value = 6 × 10^−4^), suggesting that these sequences are derived from the same *Ca*. Bathyarchaeia genome. A dominant and persistent RpS3_90%_ was also identified for each candidate order of *Ca*. Bathyarchaeia within the Lake Cadagno sedimentary sequence (Figure 3). Noteworthy exceptions appear when two relatively distant RpS3_90%_ sequence clusters replace the dominant “*Wuzhiqibiales*” sequence cluster at 40 cmblf and the dominant “*Xuanwuarculales*” RpS3 sequence cluster is absent from the 582 cmblf sample.

In our sediments, two *Ca*. Bathyarchaeia populations (expressed as RpS3_90%_ clusters) exhibit significant correlations (corrected *p* <0.01) with the δ^13^C-TOC ratios reported in this study (see Table 1) suggesting their potential involvement in carbon cycling in the sediments. These include negative correlations with δ^13^C-TOC ratios were observed for “*Baizomonadaceae*” RpS3_90%_ sequence cluster 4C (r = −0.84, corrected *p* = 3.4 × 10^−4^) and “*Wuzhiqibiales*” RpS3_90%_ sequence cluster 1A (r = −0.71, corrected *p* = 0.006). The significant negative correlations suggest a link between relatively ^13^C-depleted organic matter and the presence of these groups.

### Microbial functional potential in Lake Cadagno sediments

**Figure 4:**
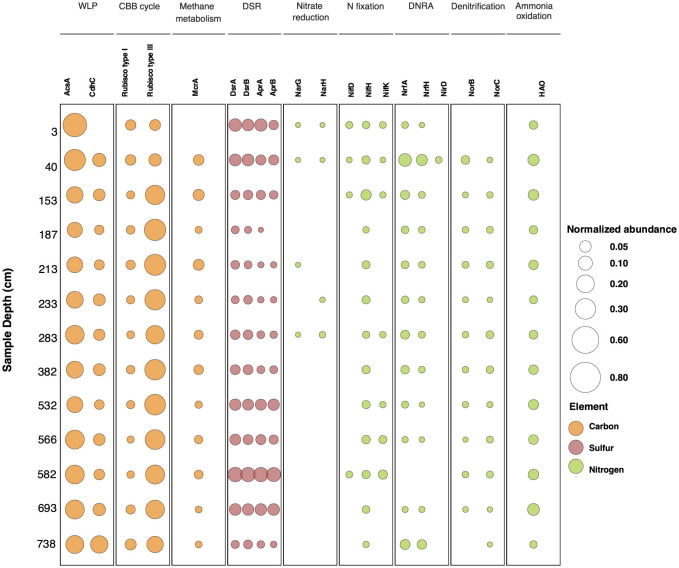
Normalized predicted protein abundance for biological carbon, sulfur and nitrogen cycling marker genes with sediment depth. The circles and their respective sizes indicate the normalized marker gene protein cluster relative abundance to the RpS3_90%_ total abundance at a given depth. The circle colors indicate the cycled elements. WLP, Wood Ljungdahl Pathway; CBB cycle, Calvin-Benson-Bassham Cycle; DSR, Canonical dissimilatory sulfate reduction; DNRA, dissimilatory nitrate reduction to ammonia.

To evaluate whether specific functional traits are enriched within the Lake Cadagno sediment microbial communities, a characterization of high-order functional categories proposed by the Clusters of Orthologous Genes (COGs) database (Galperin et al., 2021) was performed. Despite the differences in taxonomic composition between samples at 3 cmblf and 40 cmblf compared to sediments below 40 cmblf (Figure 1), the functional profile in terms of COGs remains stable throughout the sediment sequence (Supplementary Figure 4), indicating consistent patterns in the broad functional potential of the microbial community. As biogeochemically relevant gene abundances are more likely to change with changing environmental conditions, a functional profile based on a curated set of biogeochemical marker genes (Zhou et al., 2022) was additionally constructed and analyzed. Within this profile, the per sample carbon-, sulfur-, and nitrogen- cycling marker gene relative abundances are reported as the total coverage of all predicted proteins within a given marker gene protein family (protein_tot_) divided by the total coverage of all ribosomal protein S3 sequences (RpS3_tot_) in a sample (Figure 4).

The COG category for Carbohydrate Transport and Metabolism (COG category “G”) was the only category related to nutrient acquisition and metabolism with a significant, positive correlation to sample depth (corrected *p* = 0.039) (Supplementary Table 5). Ten of the “Carbohydrate transport and metabolism” (G COG category) protein clusters were positively enriched with sediment depth (*q*-value < 0.05, Supplementary File 3). Three of these ten protein clusters are related to *Ca*. Bathyarchaeia (Supplementary Figure 5) and correspond to a phosphoglucosamine mutase (Normalized enrichment score = 0.81, *q*-value = 0.04), a 6-phosphofructokinase (Normalized enrichment score = 0.41, *q*-value = 0.038), and a cofactor-independent phosphoglycerate mutase (Normalized enrichment score = 0.34, *q*-value = 0.03). The remaining seven protein clusters are related to other bacterial groups including *Planctomycetes, Deltaproteobacteria*, and *Bacteroidetes*. On the other hand, 30 protein clusters had negative, significant correlations with sample depth. From these, the 10 proteins with the highest enrichment scores are related to bacterial groups such as *Deltaproteobacteria, Planctomycetes*, and *Chloroflexi*, and archaeal groups such as *Helarchaeota* and *Methylomirabilis sp*. (Supplementary File 3). Three carbohydrate transport and metabolism predicted proteins derived from *Ca*. Bathyarchaeia were additionally found to be depleted with sample depth (Supplementary Figure 5). These proteins correspond to Major Facilitator Superfamily (MFS) transporters (Normalized enrichment score = −0.33, *q*-value = 0.04 and Normalized enrichment score = −0.41, *q*-value = 0.024) and to a Triose Phosphate Isomerase (TIM) barrel protein (Normalized enrichment score = −0.33, *q*-value = 0.04).

### Microbial potential for carbon fixation and methanogenesis

Predicted proteins of the archaeal carbon monoxide dehydrogenase (CdhC) and bacterial carbon monoxide dehydrogenase (AcsA) were used as markers for microbial carbon fixation via the Wood-Ljundahl pathway (WLP). The associated proteins are part of the five-subunit enzyme complex dehydrogenase/acetyl-CoA synthase (CODH/ACS complex) responsible for the formation of Coenzyme A (CoA) to form acetyl-CoA in the WLP (Adam et al., 2018). These genes have been shown to be also involved in the oxidation of CO to CO_2_ in methanogenesis and anaerobic oxidation of methane and acetate (Zhou et al., 2022; Bährle et al., 2023) as well as in the reverse direction of the canonical WLP during organo-heterotrophic growth (for a review, see Borrel et al., 2016). A total of 232 unique AcsA and 60 CdhC predicted protein sequence clusters (90% amino acid sequence identity) were observed within the sediments. While a significant anti-correlation between AcsA and CdhC normalized abundances was not observed, the trends indicate that AcsA gene protein cluster abundances decrease below 153 cm and then stabilize, whereas CdhC abundances increase with depth and are linked to a decrease in *Methanomicrobiales-*related CdhC and an increase in *Ca*. Bathyarchaeia-related CdhC (Figure 4; Supplementary Figure 6).

A significant correlation (r = 0.80, corrected *p* = 0.001) between methanogenic CdhC sequence clusters (sequence clusters belonging to *Methanomicrobiales*) and the methanogenesis marker gene methyl coenzyme M reductase (mcrA) protein cluster abundance was found. In our dataset, McrA protein sequences that are closely related to *Methanomicrobiales* account for 72% of the total protein sequence abundance in samples between 40 cm and 582 cm and between 693 cm and 738 cm, whereas in the depth interval between 532 cm and 566 cm protein sequences associated with *Methanosarcinale*s are dominant (40%-80% of the total protein sequence abundance) (Supplementary Figure 7). The results from the metabolic pathway completeness analysis suggest that methanogenesis via acetate may be the predominant metabolic pathway in the sediments, as the completeness values are > 0.99 at all depths except at 3 cm and 738 cm (Supplementary File 4). Methanogenesis via trimethylamine is another potentially preferred pathway in sediments between 40 cm and 532 cm (Supplementary File 4).

Archaeal RuBisCO Type III, mainly found in archaea, is the most abundant archaeal C-cycling marker gene observed within the Lake Cadagno sediment sequence. The presence of this protein is frequently associated to the Calvin-Benson-Bassham cycle (CBB cycle); however, it has also been shown to be involved in other processes such as the Reductive Hexulose-Phosphate Pathway (RHP) and the Pentose Phosphate Pathway (Sato et al., 2007; Kono et al., 2017) (Figure 4). Although calculated for the whole-metagenomic dataset rather than an individual genome, the potential prevalence of the CBB cycle in Lake Cadagno sediments is higher (completeness > 0.91) and therefore more likely than the reductive 3-hydroxypropionate (PBP) pathway (completeness <0.50, Supplementary File 4). A significant anticorrelation in the abundance of Type III RuBisCo and AcsA predicted proteins was also observed (*r* = −0.91, corrected *p*-value = 1.4 × 10^−5^) (Supplementary Table 4). In total, 89 Archaeal Type III RuBisCO sequence clusters (90% amino acid sequence identity) were identified with 30 out of 89 of these clusters belonging to *Ca*. Bathyarchaeia and accounting for 80% of the total Type III RuBisCO abundance. Of these sequences, three sequence clusters related to *Ca*. Bathyarchaeia are especially abundant within three distinct depth intervals (Supplementary Figure 8); however, a strong correlation between these Type III RuBisCO abundance patterns and a *Ca*. Bathyarchaeia RpS3_90%_ sequence cluster was not identified.

### Microbial potential for sulfur cycling

Dissimilatory sulfate reduction (DSR) is an energy-yielding process that reduces sulfate to sulfide (Jorgensen et al., 2019), and frequently used DSR marker genes include the dissimilatory sulfite reductase alpha subunit (*dsrA*), dissimilatory sulfite reductase beta subunit (*dsrB*), adenylyl-sulfate reductase alpha subunit (*aprA*), and adenylyl-sulfate reductase beta subunit (*aprB*) (Neukirchen et al., 2023). Throughout the sediment sequence, a strong correlation between the abundance of adenylyl sulfate reductase subunits (AprA and AprB, *r* = 0.92; corrected *p* = 4.6 x 10^−6^) and dissimilatory sulfite reduction subunits (DsrA and DsrB, *r* = 0.97; corrected *p* = 2.6 x 10^−8^) provides additional support for continuing DSR at depth (Supplementary Table 4), despite the rapid decrease in sulfate concentrations in the first 20 cm of the sediment core (Table 1, Berg et al., 2022). Dissimilatory sulfate reduction to APS and dissimilatory sulfate reduction to sulfide are the two pathways for sulfate reduction that show highest completeness in the sedimentary sequence (Supplementary File 4), this result is consistent with the presence of marker genes for DSR in the complete set of analyzed samples (Figure 4).

A compositional shift in marker genes for DSR is observed with sediment depth. Forty-seven unique DsrB predicted protein sequence clusters (90% amino acid sequence identity) and 42 unique DsrA protein sequence clusters (90% amino acid sequence identity) were identified, the majority of which are related to *Deltaproteobacteria* (25/47 and 23/42, respectively). In the youngest samples (3 cmblf and 40 cmblf), DsrB predicted protein sequences related to *Chloroflexi, Deltaproteobacteria*, and *Desulfobacca acetoxidans* are abundant but *Deltaproteobacteria*-related DsrB dominate depths > 40 cmblf and reach a maximal normalized abundance at 582 cmblf (Figure 4, Supplementary Figures 9, 10). *Nitrospirae*-related DsrB sequences also become prominent in the deepest (693 cm and 738 cm) samples.

Apr is an additional DSR protein that is responsible for catalyzing the oxidative binding of sulfate to AMP to generate APS which is subsequently converted to sulfate by the ATP sulfurylase (Meyer and Kuever, 2007). Although *Deltaproteobacteria-*related Apr are the dominant Apr subtype from 3 cmblf to 582 cmblf, at depths > 693 cmblf, *Nitrospirae*-related AprA and AprB protein sequence clusters represent >70% of the per sample sequence abundance for these genes (Supplementary Figures 11, 12).

Protein-encoding genes involved in the re-oxidation of reduced sulfur species were also identified in Lake Cadagno sediments; however, genes (*soxA, soxB*, and *soxC*) which are commonly used for sulfur oxidation (although not limited to this metabolic function) were far less prevalent with SoxA and SoxC predicted protein sequences identified in only 3/13 and 2/13 samples respectively, and SoxB predicted protein sequences identified in only the 3 cmblf sample (Supplementary Figures 13–15). Richness of the Sox predicted proteins was also less than the richness of predicted proteins involved in DSR and most of Sox sequences were related to *Chlorobi, Betaproteobacteria*, and *Gemmatimonadetes* (Supplementary Figures 13–15).

### Microbial potential for nitrogen cycling

The complete denitrification pathway involves the reduction of nitrate to nitrogen through a series of N-containing intermediates (${\text{NO}}_{3}^{-}$ to ${\text{NO}}_{2}^{-}$ to NO to N_2_O to N_2_). Despite the prevalence of nitric oxide reductase predicted proteins (NorB and NorC) in samples between 40 cm and 693 cm, genomic evidence for the canonical denitrification pathway is sparse (Figure 4). Predicted protein sequences of nitrate reductase subunit alpha (NarG) were identified at relatively low abundances in four of the 13 samples (Figure 4, average NarG_tot_/RpS3_tot_ = 0.00241 ± 0.00023) and the cytochrome-type dissimilatory nitrite reductase (NirS) was only observed in the 40 cmblf sample (NirS_tot_/RpS3_tot_= 0.00272). Instead, the enzyme reported to catalyze the reduction of nitrite to ammonia in a process known as denitrification to ammonia (DNRA, Mohan et al., 2004), cytochrome c nitrite reductase (Nrf), was found across all sample depths except 582 cmblf (Figure 4). In total, 50 NrfA predicted protein sequence clusters (90% amino acid sequence identity) and 25 small subunit NrfH predicted protein sequence clusters (90% amino acid sequence identity) were identified at similar abundances and depths (Figure 4). Both predicted proteins show a maximal abundance at 40 cmblf (NrfA/RpS3_tot_ = 0.15 and NrfH/RpS3_tot_ = 0.10) and are mainly related to *Planctomycetes, Chloroflexi, Deltaproteobacteria* and *Bacteroidetes* (Supplementary Figures 16, 17).

Microbial cycling of ammonia in the Lake Cadagno sediments may also occur through nitrogen fixation and ammonia oxidation. Although the nitrogenase electron donor subunit (NifH) was identified in the predicted protein pool of all Lake Cadagno sediment samples, the catalytic subunits of nitrogenase, NifD and NifK, were only detected with NifH in four of the thirteen samples (3–158 cmblf and 582 cmblf, Figure 4). The enzyme hydroxylamine oxidoreductase (HAO) which catalyzes the oxidation of NH_2_OH to ${\text{NO}}_{2}^{-}$ and is a marker gene for ammonia oxidation was detected in all samples with an average HAO_tot_/RpS3_tot_ of 0.063 ± 0.029. HAO is frequently linked to aerobic ammonia oxidation (Junier et al., 2010) but a variant of the enzyme is also involved in anaerobic ammonium oxidation (anammox) (Sui et al., 2020; Kartal and Keltjens, 2016). Taxonomic assignment of Lake Cadagno HAO predicted proteins revealed a close relationship to *Planctomycetes* and may indicate that anammox occurs within the sediments (Supplementary Figure 18).

## Discussion

Despite the drastic changes in sedimentary sources (lacustrine vs. mass-movement deposits) and a transition from bacteria-dominated communities in the top 40 cm to *Ca*. Bathyarchaeia-dominated communities in deeper layers of the 8,000-year sediment sequence of Lake Cadagno (Figure 1), dominant organic matter compositions (Berg et al., 2022; Gajendra et al., 2023) and the metabolic potential of the sediment's *in situ* microbial populations (Figure 4, Supplementary Figure 4) are conserved. Broadly speaking, Lake Cadagno's sedimentary microbial communities have the genomic potential to perform bacterial and archaeal carbon fixation via the Wood-Ljundahl pathway (WLP) and the Calvin-Benson-Bassham cycle (CBB cycle), canonical dissimilatory sulfate reduction (DSR), dissimilatory nitrate reduction to ammonia (DNRA) (Figure 4) and organic matter degradation; however, further studies are needed to confirm the expression and activity of these functions in Lake Cadagno. Interestingly, the taxonomic assignment of persistent marker genes shows strong vertical shifts (Supplementary Figures 6–22) that are not significantly correlated with sediment origin or any of the previously measured environmental parameters for this sedimentary sequence, originally reported in Berg et al. (2022) (Table 1, Supplementary Table 1). Given the absence of significant ecological correlations within this study, we expect that functional taxonomic replacement with depth is most likely driven by neutral processes (Liu et al., 2023), interactions between microbial populations (Martiny et al., 2023), or other non-measured geochemical and physicochemical parameters such as pH, salinity, and porewater content (Hoshino et al., 2020). As previous studies have suggested the preservation of functional traits increases ecosystem stability (Royalty and Steen, 2021), we hypothesize that the stability of functional traits with sediment depth throughout Lake Cadagno likely promotes ecosystem resilience by sustaining the core functions of the community after burial and under energy limitation; however, further activity-based analyses are needed to evaluate this hypothesis.

Although the metagenomic data indicate that a core set of metabolic functions is conserved in the Lake Cadagno sedimentary community, variation within specific metabolic pathways across taxonomic and geochemical gradients is also observed. In other environments, higher amounts of taxonomic variation within the intermediate steps of major metabolic pathways (e.g., hydrogenotrophic vs. acetoclastic methanogenesis) relative to the metabolic endpoint (e.g., methanogenesis) have also been reported (Louca et al., 2018). In Lake Cadagno, the marker gene for methanogenesis (*mcrA*) is present at relatively similar abundances throughout the sediment core (Figure 4) but a transition between metabolically distinct *Methanomicrobiales* and *Methanosarcinales* archaea is also observed (Supplementary Figure 7). *Methanomicrobiales* persist throughout the sedimentary sequence and typically perform hydrogenotrophic methanogenesis with CO_2_ (Zhang et al., 2020) while members of *Methanosarcinales* become the dominant methanogenic group within a local methane peak in Lake Cadagno's sediment (532–566 cmbss, Table 1) and exhibit the potential to perform methylotrophic, hydrogenotrophic and acetoclastic methanogenesis (Evans et al., 2019; Lyautey et al., 2021). Although the complete methanogenesis pathways of Lake Cadagno's methanogens were not recovered in this study, the prevalence of methanogenesis as a core metabolic pathway suggests that it is an essential part of the Lake Cadagno ecological network.

As with the aforementioned methanogens, the vast majority of microbial taxa in our sediments such as *Chloroflexi, Proteobacteria, Atribacteria, Aminicenantes, Ca*. Bathyarchaeia and *Firmicutes* are globally widespread in anoxic subsurface sediments of freshwater and marine environments (e.g., D'Hondt et al., 2004; Orsi, 2018; Yu et al., 2018; Orsi et al., 2020; Vuillemin et al., 2018; Suominen et al., 2021; Han et al., 2020). These similarities in microbial community composition can be partially explained by shared sedimentological properties (Hoshino et al., 2020), terminal electron acceptors, substrates, and metabolic products in deep sedimentary environments (D'Hondt et al., 2004; Vuillemin et al., 2018; Zhang et al., 2020; Dong et al., 2023; Jorgensen et al., 2012; He et al., 2022). Interestingly, a relatively small set of these taxa were identified as “persister populations” throughout the Lake Cadagno sedimentary sequence, appearing in ≥80% of the sampled sediment layers. The most abundant Lake Cadagno persister populations are closely related to the groups *Ca*. Bathyarchaeia, *Chloroflexi*, and *Atribacteria*. These groups dominate the taxonomic profiles of carbon fixation marker genes (Supplementary Figures 6, 8, 19–22), contain genes related to carbohydrate metabolism (Supplementary material 1), and have been identified as potentially versatile mixotrophs in sedimentary environments (Mardanov et al., 2020; Hou et al., 2023; Zhou et al., 2018; Nobu et al., 2016; Dodsworth et al., 2013; Fincker et al., 2020; Lee et al., 2018). Based on the prevalence of these groups throughout Lake Cadagno and other sedimentary environments (Orsi, 2018), we hypothesize that the metabolic versatility of these lineages makes them well-adapted for survival in deep marine and lacustrine sedimentary environments.

At the global scale, organic carbon availability is frequently reported as an important control on the diversity and abundance of sedimentary communities (for a review, see Orsi, 2018) and, therefore, it is also worth noting that changes in the relative functional contribution of persister populations to the organic matter pool are observed across the sediment sequence. In Lake Cadagno, the sedimentary carbohydrate content profile shows an increase in the relative contribution of levosugars with sediment depth and it is hypothesized these are potentially cellulose-derived, but their origin may differ depending on sample depth (surface vs. deep sediments) and sedimentary sources (lacustrine vs. mass-movement deposits) (Gajendra et al., 2023). An enrichment analysis of genes involved in carbohydrate cycling, transport, and metabolism shows that *Chloroflexi*-related gene abundances decrease with sediment depth while deeper sediments are enriched with genes related to *Ca*. Bathyarchaeia and other bacterial groups (Supplementary Figure 5). These results support previous reports that have identified organic matter as an important driver of sedimentary diversity patterns (e.g., Baker et al., 2015; Orsi, 2018) even though a significant correlation between Lake Cadagno's organic carbon pool and microbial diversity was not observed. Taken together, we interpret the observed persistence of microbial community functionality across the different sediment layers with different organic carbon sources (sediment layers of lacustrine vs. turbidite origin) as a sign of ecosystem resilience (Allison and Martiny, 2008) and the persistence of select taxonomic groups throughout sedimentary environments as an indication that specific niches within the sediment are uniquely filled by well-adapted subsurface lineages. However, it is worth noting that only a subset of the populations may be actively performing these specific functions at the time (Louca et al., 2018) and, therefore, single-cell or community-scale activity levels should be assessed by future studies.

## Conclusion

Our findings indicate that microbial diversity patterns within Lake Cadagno sediments follow global sediment biodiversity trends such as a decrease in bacterial diversity and abundance and an increase in the relative abundance of persistent archaeal groups with depth. These changes in microbial community composition align with decreases in total organic carbon availability in the sediment; however, energy-transducing functions related to carbon, sulfur, and nitrogen cycling were preserved independently of the taxonomic composition of the sediment samples.

While many studies have reported evidence for a convergence in metabolic features across larger spatial scales (Chen et al., 2022; Royalty and Steen, 2021; Dopheide et al., 2015; Louca et al., 2016; Nelson et al., 2016), this study indicates that shared functionality is also important at more local scales and across sharp geochemical gradients, irrespective of sediment age and organic matter sources. The absence of evidence supporting deterministic selection based on the geochemical parameters considered in this study suggests the taxonomic diversity of groups encoding the selected marker genes is most likely related to stochastic or ecologically neutral processes (Liu et al., 2023) or a more complex combination of ecological factors such as the interactions and feedbacks between individual microbial populations and/or the physiological acclimation of persisting microorganisms after burial (Martiny et al., 2023). Based on these findings we hypothesize that shared functionality across multiple microbial lineages enables Lake Cadagno sediment-hosted microbial communities to survive in energy limitation and preserve the connectivity of metabolic pathways required for biogeochemical cycling.

## Data Availability

The datasets presented in this study can be found in online repositories. The names of the repository/repositories and accession number(s) can be found in the article/Supplementary material.

## Supplementary material

The scripts used to create the figures in the manuscript, along with the metagenomic sequencing data table and the 16S rRNA gene targeted sequencing OTU table, and Supplementary Data 1 are stored in the GitHub repository https://github.com/PaulaCat/Lake_Cadagno_PFTGLC.git.
