# Supplementary material for: Persistent functional and taxonomic groups dominate an 8,000-year sedimentary sequence from Lake Cadagno, Switzerland
Source: Front Microbiol. 2025 Feb 3;16:1504355. doi: 10.3389/fmicb.2025.1504355 (PMC11843047; doi:10.3389/fmicb.2025.1504355)
Supplement: Supplementary file 1 [file Supplementary_file_1.docx]

**Supplementary Figures and Tables**


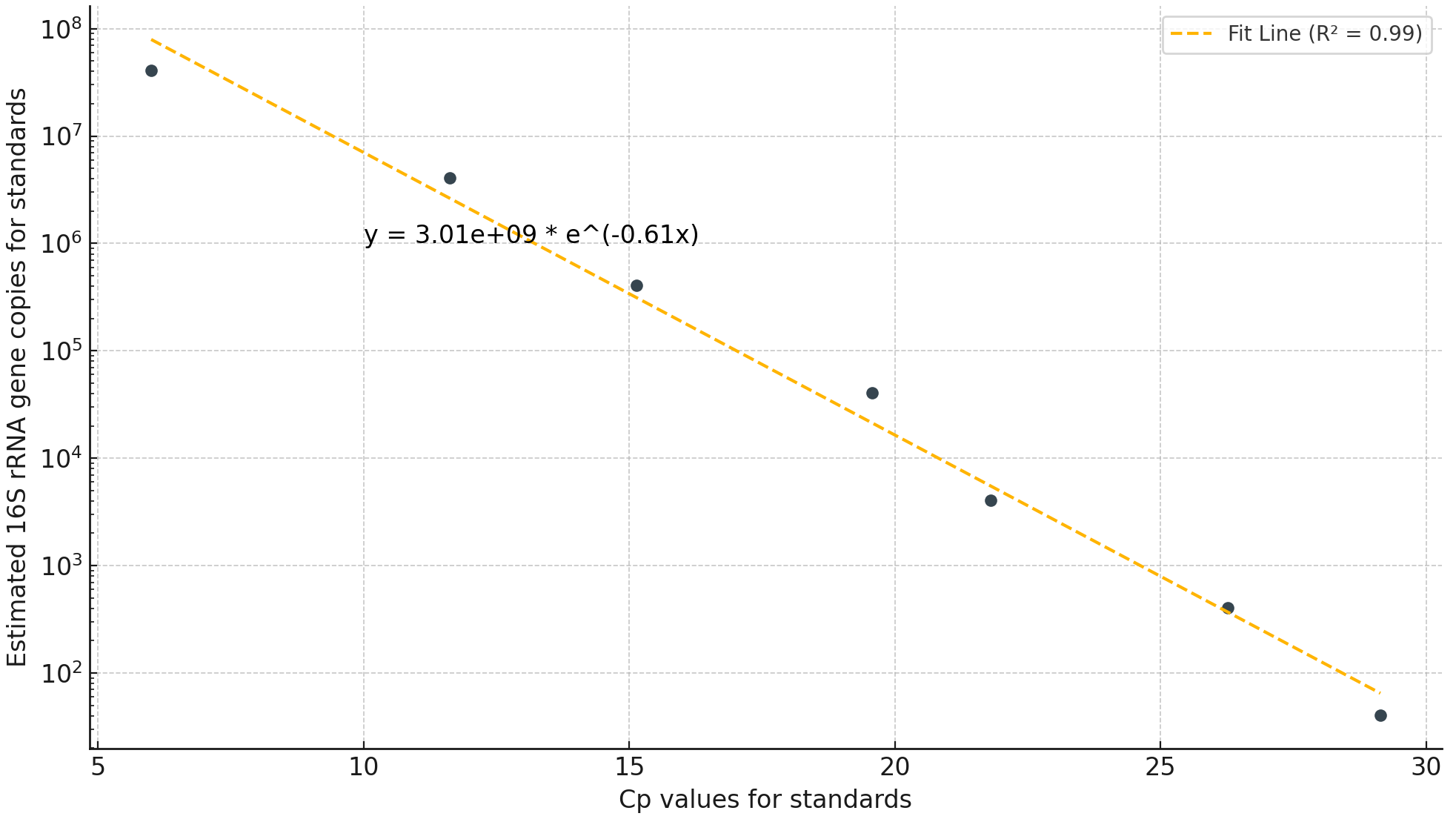


**Supplementary Figure 1.** Standard curve for prokaryotic 16S rRNA abundances using the universal primer pair 515 F (5′-GTG YCA GCM GCC GCG GTA A-3′) and 926 R (5′-CCG YCA ATT YMT TTR AGT TT-3′) by Quince et al. (2011) and Parada et al. (2016). Standards were plasmids containing equal proportions of 16S rRNA genes from *Thermoplasma acidophilum* and *Holophaga foetida* described in Han et al. (2020). The points represent the average Cp values from duplicate measurements of the standards. The equation and r^2^ values of the line are also included.

**Supplementary Figure 2.** Melting curve derivative (-dF/dT) for the qPCR standard curve.

**Supplementary Figure 3.** Relative abundance of individual *Ca*. Bathyarchaeia 16SrRNA ASVs with sediment depth.

**Supplementary Figure 4.** Relative abundance of proteins annotated based on the Clusters of Orthologous Genes (COG) categories across sample depths. Relative bacterial and archaeal gene abundances per sediment depth for the 90% identity protein clusters falling within the high-level functional categories proposed by the COGs database.

**
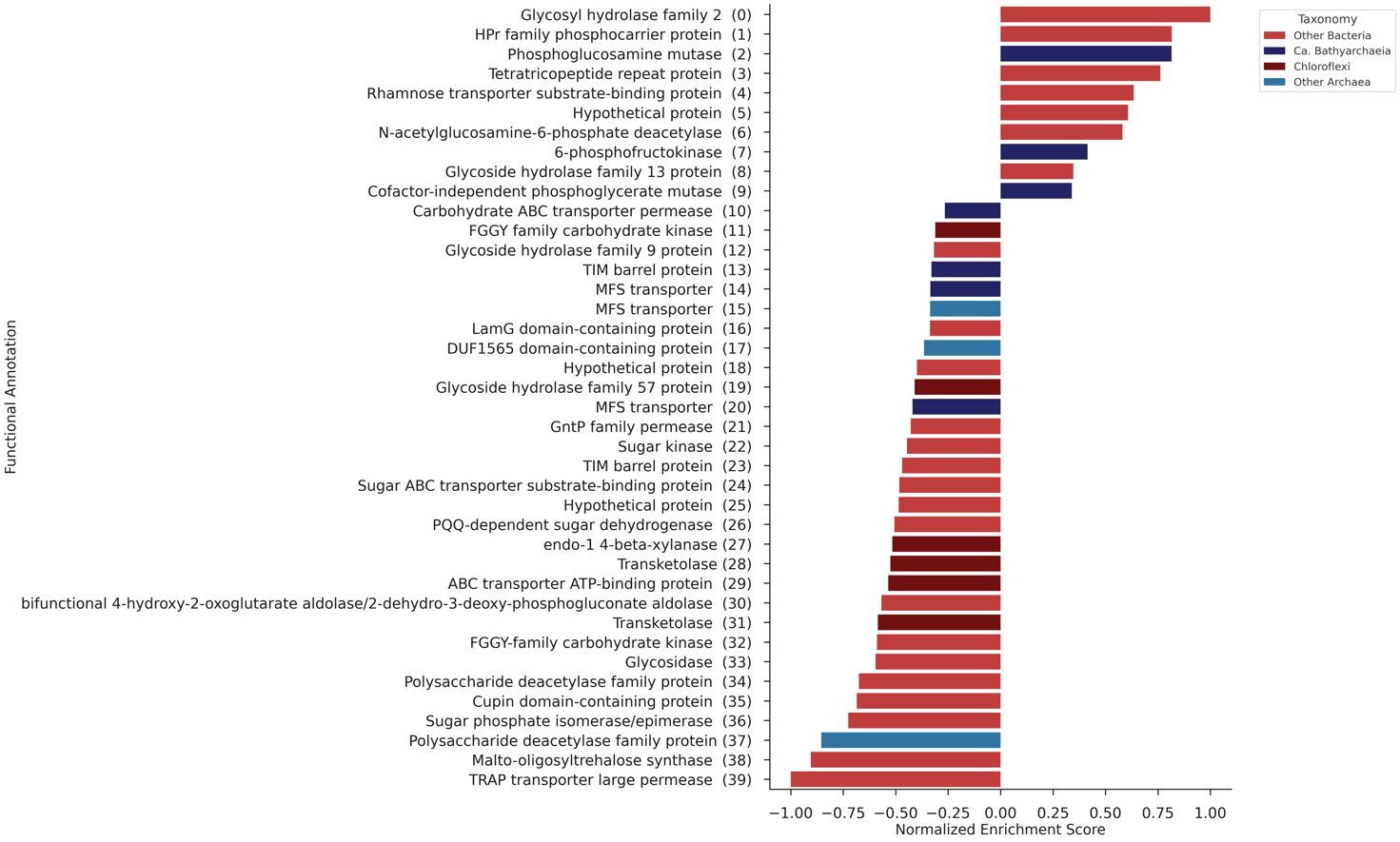
**

**Supplementary Figure 5.** Enrichment analysis for proteins classified within the Clusters of Orthologous Genes category “Carbohydrate Transport and Metabolism” (G Category) showing statistically significant normalized enrichment scores with sample depth. The colors indicate the taxonomic classification of the analyzed proteins.

**Supplementary Figure 6**. Total normalized CdhC protein sequence abundance with sediment depth.

**Supplementary Figure 7.** Total normalized McrA protein sequence abundance with sediment depth.

**Supplementary Figure 8.** Depth-related variation of 90% identity protein clusters related to Rubisco Type III.

**Supplementary Figure 9**. Total normalized DsrA protein sequence abundance with sediment depth.

**Supplementary Figure 10.** Total normalized DsrB protein sequence abundance with sediment depth.

**Supplementary Figure 11.** Total normalized AprA protein sequence abundance with sediment depth.

**Supplementary Figure 12.** Total normalized AprB protein sequence abundance with sediment depth.

**Supplementary Figure 13.** Total normalized SoxA protein sequence abundance with sediment depth.

**Supplementary Figure 14.** Total normalized SoxB protein sequence abundance with sediment depth.

**Supplementary Figure 15.** Total normalized SoxC protein sequence abundance with sediment depth

**Supplementary Figure 16.** Total normalized NrfA protein sequence abundance with sediment depth.

**Supplementary Figure 17.** Total normalized NrfH protein sequence abundance with sediment depth.

**Supplementary Figure 18.** Total normalized HAO protein sequence abundance with sediment depth.

**Supplementary Figure 19.** Total normalized AcsA protein sequence abundance with sediment depth.

**Supplementary Figure 20.** Total normalized Rubisco Type I protein sequence abundance with sediment depth.

**Supplementary Figure 21.** Total normalized Rubisco Type III protein sequence abundance with sediment depth.

**Supplementary Figure 22.** Total normalized CdhA protein sequence abundance with sediment depth.

**Section 3 – Correlations and Anticorrelations Across Sequencing Datasets, Geochemical Parameters, and Sample Depth**

| Environmental variable | *r* | *p*-value | Corrected *p*-value |
| --- | --- | --- | --- |
| TOC | 0.83 | 0.0004 | 0.0004 |
| δ^13^C-TOC | -0.17 | 0.5657 | 0.5657 |
| H_2_ | -0.59 | 0.0311 | 0.0311 |
| CH_4_ | -0.35 | 0.2396 | 0.2396 |
| CRS | -0.34 | 0.2468 | 0.2468 |
| δ^34^S-CRS | 0.21 | 0.4748 | 0.4748 |

**Table 2.** Pearson correlation coefficient (*r*) with *p*-values and corrected *p*-values for environmental variable meassured at all sample depths and 16S rRNA prokaryotic gene copies per gram of wet sediment.

| Ca. Bathyarcheia RpS3_90%_ | Environmental variable | *r* | *p*-value | Corrected *p*-value |
| --- | --- | --- | --- | --- |
| 4C | δ^13^C-TOC | -0.84 | 0.0003 | 0.0003 |
| 4C | Age mean (Cal BP) | 0.82 | 0.0005 | 0.0005 |
| 4B | Age mean (Cal BP) | 0.81 | 0.0007 | 0.0007 |
| 4B | δ^34^S-CRS | -0.75 | 0.003 | 0.003 |
| 1A | δ^13^C-TOC | -0.71 | 0.006 | 0.006 |

**Table 3.** Pearson correlation coefficient (*r*) with *p*-values and corrected *p*-values for RpS3_90%_ *Candidatus* Bathyarchaeia clusters present in more than half of the samples and environmental variables meassured for all sample depths in the sediment sequence. Only pairs with significant correlations are displayed.

| Marker gene 1 | Marker gene 2 | *r* | *p*-value | Corrected *p*-value |
| --- | --- | --- | --- | --- |
| CdhC | CdhA | 0.91 | 1.24e-05 | 1.24e-05 |
| DsrA | DsrB | 0.97 | 2.58e-08 | 2.58e-08 |
| DsrA | AprB | 0.91 | 1.34e-05 | 1.34e-05 |
| DsrA | AprA | 0.96 | 4.52e-08 | 4.52e-08 |
| DsrB | AprB | 0.93 | 2.86e-06 | 2.86e-06 |
| DsrB | AprA | 0.94 | 1.16e-06 | 1.16e-06 |
| AprB | AprA | 0.92 | 4.59e-06 | 4.59e-06 |
| Rubisco Type III | AcsA | -0.91 | 1.38e-05 | 1.38e-05 |

**Table 4**. Pearson correlation coefficient (*r*) with *p*-values and corrected *p*-values between marker gene protein clusters exhibiting significant correlations.

| COG category | *r* | *p*-Value | Bonferroni corrected *p*-value |
| --- | --- | --- | --- |
| Carbohydrate transport and metabolism | 0.375 | 0.005 | 0.0393 |
| Amino acid transport and metabolism | 0.372 | 0.008 | 0.0622 |
| Signal transduction mechanisms | 0.367 | 0.008 | 0.0622 |
| Secondary metabolites biosynthesis, transport and catabolism | 0.347 | 0.009 | 0.0697 |
| Coenzyme transport and metabolism | 0.317 | 0.008 | 0.0622 |
| Inorganic ion transport and metabolism | 0.026 | 0.37 | 0.9751 |
| Nucleotide transport and metabolism | 0.017 | 0.403 | 0.9838 |
| Lipid transport and metabolism | -0.060 | 0.565 | 0.9987 |

**Table 5**. Mantel test results for distance matrices between sample depth and COG categories related to nutrient acquisition and metabolism, showing correlation coefficients (*r*), p-values, and corrected *p*-values.
