# Supplementary material for: Persistent functional and taxonomic groups dominate an 8,000-year sedimentary sequence from Lake Cadagno, Switzerland
Source: Front Microbiol. 2025 Feb 3;16:1504355. doi: 10.3389/fmicb.2025.1504355 (PMC11843047; doi:10.3389/fmicb.2025.1504355)
Supplement: Supplementary file 2 [file Supplementary_file_2.docx]

**Supplementary File 2: Results 16SrRNA gene sequencing data**

The microbial community profile based on the 16S rRNA gene amplicons shows a decrease in the bacteria-archaea ratio with sediment depth. We recovered a total of 6147 16SrRNA gene amplicon sequencing variants ASVs (693628 reads) and 3480 97% identity operational taxonomic units (OTUs) distributed across 34 phyla and 17 candidate phyla, that were present in the sediment layers. Twenty bacterial and archaeal phyla, comprising both phyla and candidate phyla, were detected across all sample depths. The microbial community in terms of 16S rRNA gene ASVs shows highest in richness (expressed as number of ASV per sample) and alpha diversity values at 3 cmbss (Richness:1267, Shannon-Wiener index (H′): 6.16) and 40 cmbss (Richness:1407, Shannon-Wiener index (H′): 6.07) (see figure X). A decrease in both metrics was observed in the samples spanning depths ranging from 153 cmbss to 738 cmbss (Richness: 230-821, Shannon-Wiener index (H′): 4.57-3.11) (Supplementary Table 1).

Microbial diversity in samples at 3 cmbss and 40 cmbss differs from the microbial community composition observed in samples below 153 cmbss. The microbial community at 3 cmbss is composed by representatives of bacterial groups such as *Deltaproteobacteria* (17%), *Bacteroidetes* (10.26%), *Verrucomicrobia* (7%), and *Ignavibacteria* (7%). In sediments at 3 cmbss, most of the representatives from Deltaproteobacteria constitute sulfate reducers from the groups *Syntrophobacterales* and *Desulfobacterales* (40% and 47% of the total sequences from Deltaproteobacteria). The high abundance of these microorganisms is likely related high sulfate concentrations at this depth (1.3 mmol/L) (Berg et al. 2020). The archaeal groups *Pacearchaeota* (6%), *Woesearchaeota* (2.6%), and *Euryarchaeota* (3.5%) exhibit high relative abundance values at this depth. At 40 cmbss, there is a shift in the microbial community composition, which is likely related to a decrease in total organic carbon (TOC) concentrations from 17% at 3 cmbss to 1.7% at 40 cmbss (Berg et al., 2022). Here, the relative abundances of *Deltaproteobacteria* (9%), *Ignavibacteria* (0.4%), and *Verrucomicrobia* (1.2%) decrease, while the relative abundances of *Dehalococcoidia* (5%), *Spirochaetes* (6%), *Atribacteria* (10%), and *Bathyarchaeota* (3%) increase.

Members from *Atribacteria* and *Candidatus* Bathyarchaeia become dominant in sediments below 153 cmbss. In the analyzed sediment samples, the Atribacteria exhibited dominance at depths down to 582 cmbss (12%-33%), with their relative abundance declining to below 6% at 693 cmbss and 738 cmbss. The decline in the relative abundance of *Atribacteria* in the two deepest samples coincides with an increase in the relative abundance of Firmicutes to 21% at 693 cm and 18% at 738 cm. Members of the Bathyarchaeia group are also present in all samples located below 153 cmbss, comprising relative abundances ranging from 18% to 50% of the total 16S rRNA gene ASVs per sample.


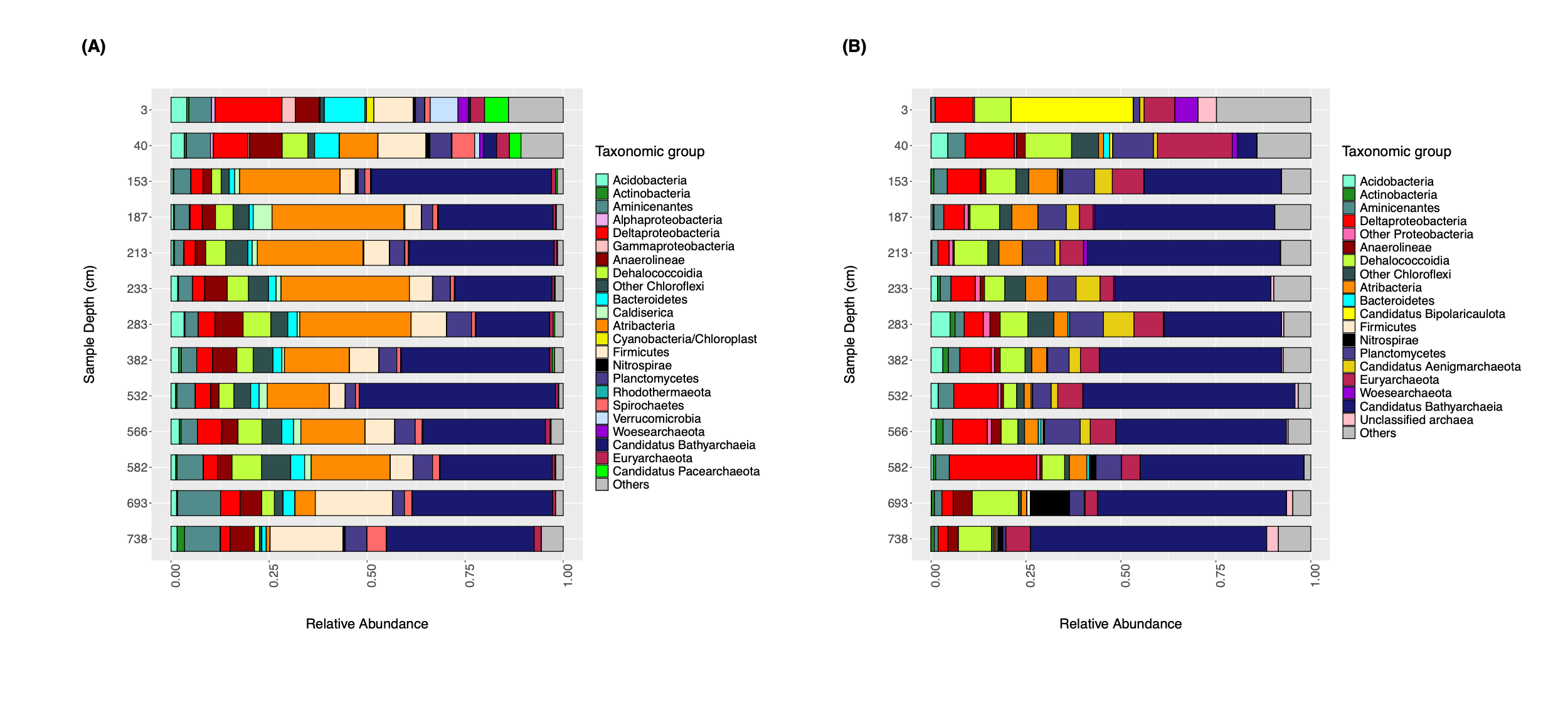


Figure 1. Relative abundance of 16S rRNA gene sequences with sediment depth.
