# Supplementary figures and images for: Persistent functional and taxonomic groups dominate an 8,000-year sedimentary sequence from Lake Cadagno, Switzerland
Source: Front Microbiol. 2025 Feb 3;16:1504355. doi: 10.3389/fmicb.2025.1504355 (PMC11843047; doi:10.3389/fmicb.2025.1504355)

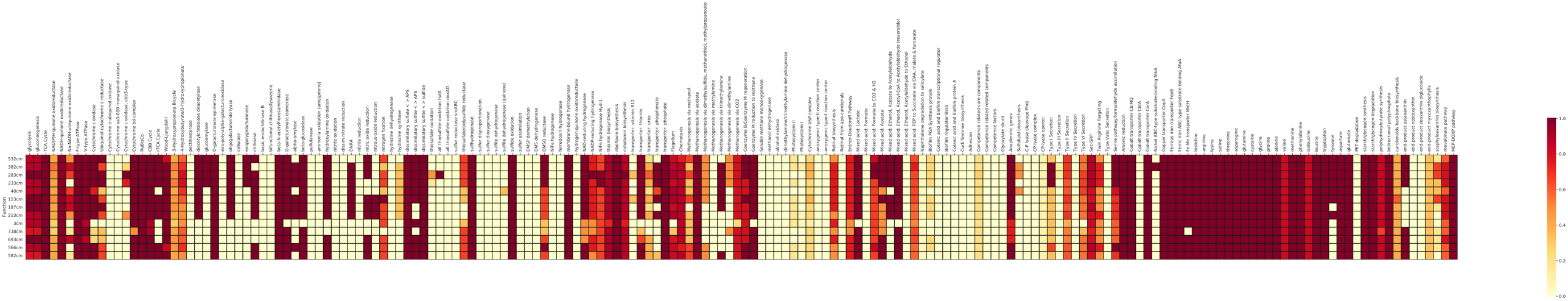

Supplement: Supplementary file 5 [file Data_Sheet_1.pdf]
