# Supplementary material for: Persistent functional and taxonomic groups dominate an 8,000-year sedimentary sequence from Lake Cadagno, Switzerland
Source: Front Microbiol. 2025 Feb 3;16:1504355. doi: 10.3389/fmicb.2025.1504355 (PMC11843047; doi:10.3389/fmicb.2025.1504355)
Supplement: Supplementary file 6 [file Data_Sheet_2.pdf]

>SOL_1_10_cov_8.224951_N_5124
MVTAKKLPRWRGFNLLSKFMVHHSGPFPEEDFRVMHDWGFDFARLPMDYRCWVQDGDPRRFNEAVLAE
VDEAVELGRRYGIHICINFHRAPGYTVARPQEALSVWTDPEAREICALHWRTFARRYAGIPSDQLSFN
LFNEPARVGDHGFTPEHYREVALLLTDAIWSEDADRLVICDGYLWGRIPVPQLADLGVAQSTRAYEPH
WLSHYKAQWVQGTADMPEPRWPGSSPHDPQDKWDIERLRTYWRPWKELADSGVGVHCGEGGPYHTVPH
PVALAWLEDCLTVLGEYGIGWALWQLRGAFGVLDSGRADVQYEEFEGHKLDRRMLEVLLRH*
>SOL_1_10_cov_8.303021_N_10633
MTISRTSTHSGRQAKAAALAAGLLFGALLLGGCADKAARARAEALRRPPVTQVLIDTTQKPAESAPIM
AALADQSVLRPAGTPPPLHTKGNQILRADGRSVRLRGVNIASLEWADEGDNVQRSLGVAIDNWGCNVI
RLPLSQDRWFGRAPTQRDGGARYRAIVDAMVAGAAERGAYVLLDLHWSDAGLWGEYIGQHSMPDTGSL
LFWRAVGQRYANHPAVLFGLYNEPHDVSWDVWLNGGELEEKVPSRTRHDPTQPLKRIRTTVRYRAVGH
RQLYDAVRAAGATQNLIVAGGLDWAYDLAGLLEGHAVRGQNIVYDSHVYPGKDWKPELSWDNAFITPS
RRLPVLVGEWGGTMAMDEQRQFVLKFVECLRENDQLSWTAWCFHPQAGPPLLRNWDYEPNETGQVILD
ELKGGDKQPEGAPER*
>SOL_1_10_cov_8.005268_N_12227
MQSVALVAILSTLAAAYAGATDSPEILHVGAVKPDVIGITVQAGRLEYGHQMPYERQPGDELRQDGKE
REVWREGRFVGWLVGKEGRLVYTEDVLVGEPLDTEWADRPGSYTVQSADDPGYAAGVHPTAVHRKSKP
SDFGRWMGWPYRAPLRHVIYLVLPTPLKPGAQYSIRFSGGKLADQAFLHDPAKTRSEAVHVSHTGFRP
DDPAKVAFLSCWLGSGGPVEYPEGLAFQVVNEADGSPAFSGRARRIHALGDPEDAYNKDYARTHVYEL
DFTPVSTPGTYRVCVDGIGCSYPFEISDAPWGKAFRVSAKGFYHQRSGIELGPPYTTYVRPRCFHPDD
GVEVYHSTCTLMDSGDGLNALGTDTGNFGNLVKGKTDQVVPNAWGGYMDAGDWDRRIQHLIVSRYLIE
LAELFPRYFGGVDLNIPESGNGLPDVVNEALFNLDCYRRMQTADGGIRGGIESGEHPRQGEASWQESL
DVMAYAPDVWSSYVYAGGAAQATLFLQSLKPDLAAAYRQSALRAMEWAEAHLPERKTYPTDVNDARNL
AAAELFRLTSEQRWHELFLKTTAFTRPDVDLYVWREHDQREAPWVYVRTDRPGMDERIRQNCHAAIIR
EAEDRLANTGRTSFRYAKYEWRPGAWGAFSDPDAVSLVRAHYLTGDTKYLRAAVLACQMGLGANPANT
CYTTGLGWDWPRHPLHIDSRVTHQDPPPGLTVFGPSDVEMHKDHWAQRIVAGYAYPDVQQWPTLEAFW
DVFWYPEISEFTVQGPMAPNAYVWGYLAGRGTH*
>SOL_1_10_cov_8.005268_N_12242
MERVLLTLACLSLAASLGRGEQEMVLRLDFDSPEQSGMMLGEVGRLAAGYQGSQSLLIEKPERTGSAV
RWLDLPADKVAGRIVTFSAVVRAEDISEKPKPWNGIKVMLILDFDGHKEYPQTNFGTGSFDWTPGQQT
VRVPAAVTGARLVLGLEEVSGRVWFDEVEVRVGRPQSGGRRSETMFKGHNLARLRGAMHRPVSNEEDI
PVLAREWGANHVRWQINWVPMKEAEQWARDLAAYDRWLDSLLPEIDKGVDLCEKYGLLVLLDLHTPPG
GRIEGGVCPLFTDRQCQDKLVEVWQRLARRYKGRACIWAYDLLNEPVEPPAGQGTVTWPELFARVTHA
IREIEPGKPVVFEPSPWGNPEGFDSITPLDLDGVIYSFHMYLPHQFTHQSLYGNPSGISYPGQVAGRY
WDKEALREAMRPAIDFQKEFNVQIYVGEFSAIRWAPGESAYNYLRDCIELFEEYGWDWAYHAYREWDG
WSVEHGPDPRDHTPTTAPTRRKQLLSDWFAKNQKPG*
>SOL_1_10_cov_8.219937_N_14538
MLSQDIARKLVPCVICIMLAGASAPATAASFVKWSPAGRGLDLGAGAGVTEVSAGGSPPVVYVIVDGV
GLSVSTDLGDSWRLLRGSAPCLAAPYAVAAHPADGKLVYATGRDEGSGLWMSGDAGATWTKCGGRDSG
MATDAVEWISIYAEDPDLIVVGHRAGRAVSVSRDGGRSWSASDMGAEVEAQVPFIVSAERWAVASRAQ
NGVRYTMDGGRTWAASQGNTDYFPGPLPVIHVGDRFFSSSHHGTNKSTDAGATWSYQMERHARVVGTM
GPILFREDREPIPGEKARLLTITMSDNYANTWEDVTGALIGLVPAALRANVTIQDAVDPYAHVRMVTA
WSSLPEQHLAFVGLGKAGLCRGLLMRTKQGPQIVNAAADPISVLAGDSATPVTVRAMVSPYSGRLKRV
YADLTTAEKGEVELLDDGAHNDGRPADRLYAGSFTLPDDLREGDKSIGIIAEDDSGRVQSVGLRLNVA
SGLERTTAWDGDMFAYGYGWCAPAGGFNFTKPQSEEFHSGTVALEFHCDVQGGWTGGGWNWHGWYPAD
AGTDIRTYRNLSFWAKVQGERVRGFDVQLLCSTYADDKSAGTAKANAADYALPAGTDLLDGRWHEVVI
PLADLYRGDSVKFDAAKAWQLNFNTWSPEPAKFSIFIDEIGFDNRRVRPHSVMVTRPEARTPSPLGPE
AADVAAEVDVAAEGTPISPYIYGAAMGDRAVAREMGLTMLRAGGNPVTPFNWKKGFSSKGADWFFQNE
GQETTPDRTWLATFHGENRRAGLATYLTIPMMGRVAKDGTSVAFDARRYPDQEMSAGVSQPTDRLPYA
GNGRQFVRGPDGEYLRDAQGTPVLRDIEPDPDDTSVEMSPAEQTDMLRFMVQQMGYGTADKGGIGVIA
LDNEPFLWHATHRGMRLKPCSYDELWERTRTYASVYKQVDPSVKVAAGTFFGWTAYFYSGLDARMVAD
GKATWEKPPDFAAHGGVPVTKWLLTKLRQHERQTGIRLADILDFHFYPQTGIYMAGTPNDPRVMEARV
QETRVMWDPTWKDPSWMGAETGKVIQLIRLMKSWVAECNPGMDVALGEYNFGGDSDVSGGVAQAELLG
IFGREGLDYAFYWFFPPMNSSPYFAYKMFRNPDGEHTAFGDRYLPSTVGSPEDVSVHAARDSKTGRLT

FVLVNKRAAKGARIALRLSKPVPRQPLVIYEYSAADKNSIGGWPEGTAEGDMIRVSLPAMSVLRFDLK
P*
>SOL_1_10_cov_8.219937_N_14554
MIRGTMIRLGLAGLALVLAVQLSALIFAQEEGPTLAHERNAKLGRGVNIIGYDPIWRDRSQARMKDEH
FRLIKEAGFDHVRINLHPFRHMGPEPDYALSGEWFATLGWAVERALANDLMVILDLHEFGSMGRRPDE
NKPRLLAAWRQLAHRYKDAPDTVLFELLNEPSRDLTPEMWNDYLAELLAMVRESNSGRTVIIGPGRFN
GIEALPVLALPEDDRNIIVTVHFYEPFRFTHQGAAWAGEAIRKLSGIEWTGTQDERDFIRGELARAQQ
WAKGHDRPILLGEFGAYDKADMSSRARWTACVARTAEEYGWSWSYWQFDSDFVVYDIDQGEWVQPIRD
ALVPAK*
>SOL_1_10_cov_8.150498_N_17976
MQGREASPRVPCRAGLLTLKLSAVSRNYWDDGEKAKMGEEVRVPPGAGGVRGDPFTAPGRFVVGCNYW
ASHAGTRMWSDWRADVVEDDLRRLSEAGLQVLRVFPLWPDFQPITLHRGFEGRPVEVRLREEPLQNAE
EGRAGIDPDAMARFDELVGMAARHGLLLNVALITGWMSGRLFVPPALEGRNVLTDALALQWQVRLVRC
FARRYRGCPTIVAWSLGNECNCMGHASRAEAWNWTAAISNALRAEDPDRPIVSGMHGLSPAPDADWSI
QDQGELLDVLTVHPYPLFTPHCGQEPLNTMRPCLHATAEARLYADVGGRPVLVDEIGTLGPMVSSEQV
AADYLKTCLFSLWAHDCHGALWWCAFDQVHLEHAPYDWTGLERELGLMRTDKTAKPVLRVLGEFRRLV
EGLPIRELPPRRTEAVCILTHGQDQWGVAYSSFILAKQAGFDVVFRYEDQALPDAAVYMLPSIAGMQV
ISRRNWLRLMERVQAGATLYVSHQSGLLQEFEKWSGLEVVARSARRGPACTRIALNGHAFSLTVQASG
AYRLDLRATRAEVLGQEDDGNPAFARARYGEGHVYFLSLPLEASLAREPGAFESQDAGAAWRVYRAFG
AAALAGRAVVESPPPVGITEHVLPGGSRVAVVINYGPSPTRARLGLAAGWRLEGVWHGGKPEAGPGWL
LQPLPANDAAILLLARA*
>SOL_1_10_cov_8.150498_N_17981
MDVPGRPALPARRPAAGARQGRRSSLAERATEAALLALVTAALCAAGEWGGPWMSSQLNAPGRPAPGR
RQPAESRADQGADPTQASFRPGEFYFRLAGRQAFLLGRNPTGWEVQQFAPLLQWAGGAGERMVRLHLT
AGMAPSSPAGEVDEQWARSWERVFDMAAENGLCVLPVFAVWADWNDGSQGEHWHYWHRNLYNAAQGGP
GRAPAELFEASECQELFLKWLAKLVARWQARGNIMAWEVFSELDLVTGSSEARAVEFVERTAAVVRNA
DARHRPITVSLSGLHDWPRLWQSNAVDLVQVHPYSADLDQAVISSVRRHLERYGKPVLIGECGLSAAP
PDPDSLTMAARASVGIRHAIWASMVSGAMNGRMLWWEDGYDQYHQLDLRTRYRDACMPVARFLEGVEF
EGFRPLEATISPGLKGAAIGNERCVLGWFRDASCTSPEWATPPVRGASVSIETAGKAQSWRVEFHETT
LGHALSAAAVSAPEGKVTVAFPPFEGSIAIKMRAEGP*
>SOL_1_10_cov_8.386450_N_20325
METVPQYGRWSKEFGLERKIDRVGFVTPEGLLETRPTFVHEPVELAYDRHGYEGLLPSGRQVAAVRFT
PVQPGRHRYRALSQGKVAEEGEFACERSDHPGYVEVSKKDPRYFALSNGAAFCPIGLNLAFPPSYPLP
RGTEHFLVSEKRATLGVREYRRWFQELARNGGNLARIYLSWDYFSPETEVAGQLEPARFARLDAVVEA
ARECGVRLKVCLEHFRTFEPAAPFAKVLKHPGDGRAPRDMDEWFRAKTWQDLWMRKVHAWFARYGDDP
TIMAWELWNELECCATQDWPMLREWARGMLRVMKEMAPRQLVTNTLGSLDRESQLCPYGDFHMDEMDF
QQVHRYLDQGADLGICHHDPVAFCADAIRVARRPDRPILMSETGGVNDCHTGPFPYYRMDARGSIFCD
VTFAPFFAGSAGTGQIWHWYEYVDQKGLWPAYRPFADLVSGIQLDAEDFKVADVSTDRAWFLCLRGDE
HLLAYVRNRQDSWYAVLRDGHAPEVLTDQTFDLSPLGVRSGKPALYRPWPEPDGEATLRGGRLRLPPF
WYGFLLKIRLGSG*
>SOL_1_10_cov_9.848021_N_20699
MAVRWSRKKAWEWYNEQPWIVGCNFTPSTAGNQIEMWQRETFDIETIMRELGWASDLGFNTARVYLHD
LVWEADPDGMKERMNILLDSAAHAGVCPVFVFFDDCWNREFSLGKQPEPRSGVHNSIWVQSPGSRVVT
DPSSWGRLKGYVGDILGSFGSDERILMWDLYNEPGNNKLGEASLGLLRSVFDWAREAGPSQPLSVGVW
FDNAALNEFQLGASDVVTFHNYNDAANLETQIKRLSGYGRPLICTEYMARTRGSRFETHLPIFKREHV
GCINWGFVSGRTQTIYPWGSTEGSPESQPWFHDVLRRDGTPFDAGEVSFIKDIIERNRSS*
>SOL_1_10_cov_6.190191_N_48077
MAIRIRASRAVVAPAGDPPSFKIQTDLPYSEVEVTIDPLLFNRILINRRRKENFYTSQELLIDKSHTA
SYTLDRNAWLLLRNYPYLYYRAIAYDAQLPANRKKVEVSVKDNDPQSAPSVYIVPSGTRPPPLKRRVS
GKLKWIRVVGNQIVNEGGDLVVLRGVNRSGMEYTDRRALDPNGVSRPTSREAAGITKDEIMEIVNYWK
ANIIRLPINQEWALTRADYLTNIDRIIEWAAAEGAYVLLSLHWFDTRREFGKTADGSINHVPPMPEEN
SVRLWSMLASRYRQEPAVLYDIFNEPHVPLADDKSYSFKPPVTEEGWLDMWHSWIRRIEATIHRENNR
ALIFVSGWDWGLNLRSFPVPFGGGRNLPNAVYSTHVYYFNTPGRSTATTADFEFWFGFTRLRSKHPIF
VGEWGGEETNLAWGKLLEQYLQNLHSFKNGIWQGLVGWTAWSWADKPLLVERGEGSRLAPDGSVLKWR

TFVMDGTHNKPTKFGEFVHSSLLKQPLPPFNVLSHIWQEFHNKASTTVYTGYTNDDRKVVENRDATGI
LQPKQRLHLCFQEKTVSTIDVMIRSGTYYACWQIIAANGNLIKEHKQFTQANYNNSTASARLQTGKHG
WLWDGRNNAANPIFVPPGIYRSRIIVKDDAGNQILQSDASIIVEGNPYLIFIMGLPKTNAELRAEFSR
PALNNRFLDPNGERTARDCWIIVYRGVENEGHVVFLGQGTEEATKAEGFPDFGAIATPHSRDFKGWIR
KNPKGVEANPDRIQIEDLSRTDELIQLFNIGGPPPNNPYCDSDPNTPYKDGVQAHGGNAKWTTNVLSV
GCTTVSPIIGSTAANPGSVWGRVRSLNSDFGNWGNPNLGENVATAGPKFLNKQTSDTTVDALISDEHA
APELKPPQHTAPLHMQPTMQQGVFGGFKGYEIPQNAAVDDDPNNQLRIRMRLQDYVNGSMYYQYHHAV
VFSHKQQQVGNGFRITVWIPRKVIRGWNGNLRNLLINGNIQIAWYIERRLIGGMVGPPELISGLYPIG
ASAGSYAALQPADIGKMERTWKFNAPLAPGRYVSVFKYKVQLRPYAAGDDTWVEEVAATDLFSVSGQP
ASIAAERIVTEGNQQFTEGVSELIITTIE*
>SOL_1_10_cov_6.190191_N_48079
MGFLERIEERRKKERVDLLAMEREEQRQEERRELEREQERQEERQRREQELHEAERKALEKTAAFRAR
YNAERRIEKSRAKRIMDYKGKLSAERMESAHSEAREAKLKELRNQELRDAQRLDVYKLEKTSELKAKL
AEERKEMAFLDTNRAEYQKRRRQEHRRAREYEIQYAKQSQENKTEYSESQKEIRRSEARQVKLKERRK
EENPKVREIKIGDTDQPRKRITRQSESRNKITPSEASKVKLKERHRPEKLEKRHEKRENSTRLGIQQN
ERSSGQRFPSGRLSGSLPWLHVHGKYLLDEMNQVVILRGVTAKGLERASPDGKLFNHPLDDVDFATLQ
DWGVTALVVTIAQDLALEGRDGAEGEDYLEALDATIQAAADVGIYTIIRLSLLSSVLPTGAGPENDVF
DPALPDQRSIDLWAVLARRYSNESAVIFDVFRTPHDPELSDSTSFLMPRFSWSVWRHWLLAMLGEVRR
EHPRALIIARGPNYDLSGFPLTYSDCSQVGNVIYATEIAPNNTQQVLVEITKINRAGCLCLLDVRTSS
FDGRFVEALSRILARDGINWISTDWKDGKTKLVERKRGHPIATPIGRAFQVALHVPPAPDANFEPNTL
WIPTL*
>SOL_1_10_cov_9.268629_N_49484
MINHLLKIFVVLVLCVGHSGAHGTVPCNDGEILYFGECAKECLKYIIQESSQGMDHGNALKLTPDTWH
SPVYRINCAEKSRRDFTRYNSLEFYFRTPSPDPGNPTFSLRTWNQRSREVKIRDYISGGVIDNTFRLV
SIPLSQLATEVWDLGNVESLVWNTDPERRIYYVDNIRLRQTEPPALITEGNWAPFPESNNVLRLTFSK
RWKQETVRDRNNYSISSLTDPSYASPVHPLNVGLHYRVESFSPSGVARNRFSVFLRLPKPIKNATSYT
LRVHGVTDEFCNLMEPTELVFNYNENSFLNTNIKVNHEGYLPRGPKVGYVGGYLGDLGGGAWAVGDNG
TIFFLDHETTDLQRVDPVVSTTLRGVSGIREDDIYAVGDDGVILNWNGTRWSRIDSPTTRDLLATHFG
PTGIGWSVGDGGVILMYEKGNWIPVPNHSSHTLRAVWAGPGDTAWAVGDGGTILRWDGKQWVSENRCT
ESDLHAIYGDQKDQLWAVGANGTVLFRRSGKWELFPSTPRTSATLRSIVTDPSGNVWIGGDDGLLWQK
SGFGSSEFLAGQSGTSQSIYGITRQHARQLWAVGAHCALLSFSSPALGWRPEPVDCPENLRAAFSIPY
GALRLPNPPPMVSLHDVTTCKTVITVPLKLETANWYLSGEDVYSFDFSALNTPGTYRAYVPGLGVSDT
FQIGNSVLDHSAYTVAHAFYYQRCGTSLTEPYAEKRFVRPRDHEYEANGRKLDAAFHESLPKTSLFAG
ETPGAMMDGHGGWHDAGDFGKYVPTAAAALWYLFTAYDMEPLKFLDGTWNIPESGNGAPDLLDEARWE
LDWLVRMQGSDGGVYHKLTSQKWFEGMPQEENAPRYFFEKTTHDTALVAAVLACAARLWKPYNKDLSD
LYLERAVKAWDFLKQHPEAIPNTGFRNPPGNTTGEYRDADDRDNRLWASAELYRTTGHTTYRKYFESW
WATNGNHTWGWNAWQHFYRCAYWAYLRSPWSDGNPGFKQEIQQGMIRNADEIVALTYSNPYRNGAKLN
VPEWIGWGSFSQSSEYAFRLLQAWSLVKDEKYLNAALLNVDTQLGANPLSLCFITGLGKRSPMDPLHH
PSIHDGVEAPFPGLPVFGVAAHLPNNQPYYIESQKDENSFPPSLNPLDPYPILRRYIDAHELVPMSEF
TIVDMAVCAAVLNLLAQGPERTR*
>SOL_1_10_cov_6.725062_N_50519
MKSNRRDFLKMMAGSAMAAALPTYAQQPKGKQAKLSKSVAQTAIPRWRGFNLLDMFTMRSKGEFPEDD
FRWIRDLGFNFVRLPACYRVWIKDGDDYKLNESMLEKLDRAVELGGKYGLHVDINFHRGPGYSVNREF
TEPFNLWKDKEALDAFCFHWQMLAKRYRGIAKEKLSFNLINEPPSVGERMSRADHERVVRTAAAAIRE
ASPDRLVVADGMSYGNEPAPELADLGIAQSTRAYQPMFISHYGASWVNSKDYPEPAWPGNGWDRKRLE
EHYQKWADLAKKGVGVHCGEGGTFNKTPHSVVLAWLRDVLEILTGHGIGLAMWNFRGSFGVIDSDRAD
VQYEDFNGHKLDRKLLELLKEFE*
>SOL_1_10_cov_10.160429_N_50913
MQIKRLLGCVVLFIAGAVLAASVNSGTAAASQVKAESASMPLPLKVVGTQILNSKNEPVRLRGVNCAC
LEWTSDGEGHIVESVRVAIDDWHVNHIRLPMSQYRWFGKAPEQNDQGKAYRALVDEIVKLCAPKGVYI
MLDLHWSNAGVWGEQIGQHSMPDEHSTEFWKDVAPIYANHPAVIYDLYNEPHDVSWDIWLNGGEITDK
PNRRNQTPITFKAVGMQQLLDIVRAAGAKNLVVIGGIDWAYDFSGILDGRQLKDPIGNGLIYANHCYN
NKNQAVETWIANMEKAAKKLPIIISEFGGAYYKPGEEPPRRRSGGMQRNDGDWLMRVLQAIEDHQWSY
TAWDFHPAAGPTLISGWDYKPTSGFGVYVKQMLDGKLPRYTPQAPAESTSEPDK*

>SOL_1_10_cov_7.780243_N_54368
MRPHTCWSISEHGYLEAPGASVLAFHDIYPEGKQGGVEIIHHGERVAACGDLRLELAPGQWGALPTAG
ERKVDRESLQVQVPLSYPKVGLSYTVRLQADGDAVRLTVDLDTPLDPAQQGPRGPEAAS*
>SOL_1_10_cov_12.077659_N_63734
MQTNRRDFLKTVAAGAAAMTVPGYAGEVKKEQKKGEYKVQKAIPRWRGFNLLEMFTMRSSGDWAEDDF
RWIRDMGFDFVRLPSCYRLWINDGDDYKINEAMLEKLDRAVELGGKYGLHVSINFHRGPGYSVNQEFT
EPHNLWKDAEPLKAFCFQWQMLAKRYKGVSKDKLSFDLINEPPSIGDRMSRADHERVVRATAAAIREA
SPDRLVVADGMSYGNEPAPELADLGIAQSTRAYQPMFISHYGASWVNSKDYPEPAWPGNGWDRKRLEE
HYAKWADLAKKGVGVHCGEGGAFNKTPHTVVLAWLRDVLEILTGHGIGLAMWNFRGSFGIIDSGRNDV
QYEDFHGHKLDRKLLSLLQDFK*
>SOL_1_10_cov_7.342617_N_73428
MRIDGACRATCRGLIALVLGLLLAGATGGPVPAERSEAGLPGSAAPPPEAAAPAVKAGDLVFAADFED
ADVLGAWSGSPRTDAGRQGGRSVVVTCADGASAAAVTVPLPAAHLRGCKIIFSAYVKAEKVSDRPQPW
NGVKFMAPIDSPSGRLWPQGAIPVGTFGWRRVVWQTFVPEDARAMTLVLGLERVTGTAWFDDVRVVVR
RPPIVRRPRPAAAGPVYTGRDLPRLRGTMVSCDPTREDLRVLGRLWNANLIRWQLLGWQPPAERTALD
AYDDFLESHLKRLDAALPWCEEYGLRVVVDLHAAPTGPPGSGRGLFNDAACQEKFIEVWRKMARRYKD
SKAVWAYDLLNEPVEDAVDEAVDDWQALAARAARAVREIDPGHAILVEPAQGGGPDGLRELVPIDVPG
VVYSVHMYLPGAFTHQGVFDEGARKWAYPGRIDGVDWDKARLEAALKPVADFQDAYGVHIYIGEFSAI
RWAPDGSAFRYLRDLIDLFEARGWDWSYHAFREWSGWSVEHGPDKADTEPAKTPTDRETLLRDWFARN
RKPAPSP*
>SOL_1_10_cov_10.274624_N_76351
MICRRKSQFSWLHLGTLIVFLLLIVAWLTTTWAIEKQGVNPFSQNRKLQRGVNIIGYDPIWGSRDKAH
FKTQYFRMIKDAGFSTVRINLHPFRHMKQDDKYTLDDSWWQVLNWAIEKALSSDLMVILDMHEFGAMG
NNPEENKDKFLAFWASGLRETPRRT*
>SOL_1_10_cov_9.126525_N_86061
MSETSGFNPKAKYAVTMWEFSWLVRRTGNEAEYADWDKVLDELAERGYNCIRLDAFPHLVAKGPDGEV
VKQFTILPQSGGFMWGNHRPVQVEPRSALVEFIGKAADRGIYVGLSSWYNSDTLGRVHMIQSPEDYAR
IWLETLDLLSDAGLHGRIVWVDICNEFPLSRWAPEPYANIFQSKRFGDLWMVLNLSRKWDEGVKQRMK
NYFDGAIMPLREKYPALKYTFSFQALGSSQMKEIDVSKFDLAEVHIWVSDYMKWVFRTGQILMLVGFP
KYPVNLRIHAKRMANLYPKHREEYIRMLEARIDFWAEWGKKNGLPLFTTEAWGPINYRDITPAGTGAE
WDWVKDIAEHGVRMASERGWQGICTSNFCQPHFVGMWADVGWHKRITDLILRG*
>SOL_1_10_cov_6.135705_N_97963
MSLFVLAMVVVPAQVALGADSSDASKGPPADDFSTIRGANYVPSYARNDVQLWLEYDPAVVDRELGYA
ARLKLNTVRVFLQYAVYERDPKLFLERLDNFLGLCDKHHIRMMPVLFDSCFGDFPDLVNYKDKNWMAN
PGQNRLGREHWPKLEQYVRDVVGSHKDDRRIVMWDVMNEPTCTSFTKPQDKELIWTFLRHMLDYVKEV
DPAHPRTVGVEHTSLIPKVLDKIEVVSTHNYRQDLREDLRAVKELARKHGKPVIINEVAGRPKQPYSY
VMPILAEEKVGWCFWELMIGRTQFTQGATPYQGVIYPDGTCFDATEVMHIVHPGRTGLDPRQVAADVG
LPQRPSPWSIERAWAWYKARPWTVGINYHPSNVVNTTELWSADTFDEETIDRELALAEKTGFNACRTN
LQYLVWKHDPQGMKKRMERFLAIAEKHGIAVIFVPFDDCAFGDPPTTEPYLGKQKEPVPGMIMSSWTP
SPGLKAVTDRAAWPDLEKYVKDILGTFAQDKRVLMWDLYNEPGNSGMGNRSLPLVEATFGWARQADPI
QPLSMGIWNDRLRDLNRAMAERSDIITFHAYTNYQGMRAAIARHKAHQRPVICTEWMTRHSGARWESD
LPLFRREAVGCFSWGLVNGRMQCQFTWWDKRGAPEPKLWFCDLYHKNGRSYDPKEIEAIRKTTADKRI
DFTAADYSRPQPEAGQILDTDRRIK*
>SOL_1_10_cov_6.405666_N_110214
MNLSALAALGLFLAMAGLAVAGPRVLIPTATDQPAAWRYTTTTPPTDWNQPGFDDKTWVVGKAGFGVT
DQVTAPSVIGTPWRTADIWLRNEIEAPSPLQFETAALTVRHDEDVEIHVGGKLVFAAPGFNTAPMAYD
VTKGLREVFKPGKNLVAVHVHQTTGGQYIDLGLVLDPKEKPALQVDPNDFLLVERLRASRWPPEKAWR
WYGEVGPISGCNYLPRTAVNTTEMWQAESFDPPTMDEELGWAEKVGMNSVRVFVQYIVYEADPDGLVK
RMDEFLRIADKHGIRTMVILFDDCFLPEPKLGKQPDPVPGVHNSQWTSSPGQTRKRRESWPALERYVK
GFVGHFARDRRVLAWDVYNEAAAQSRPLVAAAFAWARAAEPRQPLTSCWQAEDLSDVLTFHDYGAPNP
QILARLVAERPALCTECIARGGGSRFDNVLPAFAEKGIGWYMWGLVRGRIQTYYPWGSPRGAPEPDPW
HHDLLQPDGTPYRPAEIEAIQRFPKQFKPRK*
>SOL_1_10_cov_4.607485_N_126026
MDNRREFLKMAAAGAVAAALPGYAQEPKGKGKLGQPPAQTALPRWRGFNLLEMFTMRSKGDWAEDGFR

WIRDFGFDFVRLPTCYRLWIEDGDDYKIHEPMLEKLDRAVELGGKYGLHVSINFHRGPGYSVNREFTE
PHNLWKDKTALDAFCFHWQLMAKRYKGISKDKLSFDLINEPPSVGEQMSRADHERVVRTTVKAIREIN
PDRIVIADGMSWGNDPAPELADLGIGQSTRAYQPMFISHYGASWVDSKNYPAPAWPGNGWDRKRMEEH
YQRWADLAKKGIGVHCGEGGAYNKTPHKVVLAWLRDVLEILTAHGIGLSVWNLRGSFGIVDSDRKDIK
YEDFHGHKLDRKLLELLQEFK*
>SOL_1_10_cov_5.250449_N_136077
MIRIEGSHFKDELGRTLMVRGVNLGGSSKVPFNPNGATYICDGFFDHRKVSFVGRPFPLDEADEHFTR
LREWGFTFLRFLVTWEAIEHTGPGIYDEGYLDYVRAVVKKAGEYDMHIFIDPHQDVWSRFSGGDGAPG
WTTEVVGFDITRFAESGAAIVHATHGDPFPKMIWPTNGSKLAAATMFTLFFGGNDFAPKTKVYGEPVQ
EFLQRHYINAIQQVALRLKDLPNVIGYDTLNEPLSGYIGWQDLNATDGTLTLGTSPTPFQGMLLGAGV
PQEVAFWEIGLTGIKRTGRRLLNAAKVRAWQAGHDCIWRQNGVWDFDASGKPQLLRPDHFWRVKGCEV
DFTQDYYRPFANRYAPAIRTIDPKALIFVETGPDTQPLRWNTGDAPNVVYAPHWYDGYVLVKKEFNPF
LAVNAFTQKVVFGRGAIHKSIAEQLASLKRGARQELGDVPMVLGEFGIPFDLEDKKAYRTGNFNAQIK
ALDRSFQAVETNLLNCTLWNYTADNSNARGDQWNDEDLSIFSRDQQTNPEDINSGGRALQAAVRPYPR
ATAGVPLRMAFDIKRRVFTVEFRHDPQVTAPTEVFIPNLHYPQGYKVELSDGTYEINREKQTLIYRHS
VERGEHTVRIKP*
>SOL_1_10_cov_3.181384_N_139709
MRIRTDKEDNKIVYADRVGFSISPEAATSCVIRKDDAMSPIRPRHMKVLCALQFCWSIGAAAQNFSGG
FSFFLPPDDATSQKFMPDFPARPITGSDFVTTDADGHFSATGRRIRFFGTSLTFDGAFPDRAKAAFVA
GRLRKMGFNLVRFHHIDNSWSQGSLFV
>SOL_1_10_cov_3.676079_N_140230
MKLINNIKMIFIVLFGFSVIPLQAQRITVNGTRFAVDGKEIWLNGANTPWHRWNDFGTGNYDHAWWNS
HMQTLNDNGINCIRVWISCNGGGIKINPDGTVTGVTATFFSDLDQFFALARSHRIYIFATLLSFDHTK
NGNPNYQAFRACFTDNGRTGTLVDAYVVPFVNRYKENPYLFAIDACNEIEWDHENAEAGKIAWERLQY
LVARMAVGVHENSPVLFTLGSAAVKWNSDSPGEGNKWKNSALQAQLNDPKAFLDFYSPHWYGWVVRWF
GNPCVTSPAGYGIGDRPCVIGECPARGMFNQDAQGKDILVNPPADMYEMAHQKGWQGVFPWTSNGVDD
NGGIADFGEATRAFRDNHPGLVDPSETKVNSAPDRSPEPSRLSCYPNPLPSGVYLCCLNLGKDRLVSK
VLMVQ*
>SOL_1_10_cov_15.065815_N_144991
MTNVECRILNDEGMHTAARLWITFCLSTAIFVASAGGGDLEWVKVSQDKKGFVLEGSGERFVPLGFNY
DHDDEGRLIEDYWHPEWSTVEEDFLEMKRLGANVVRIHLQAGRFMREPNEPNEASLERLGRIVALAER
VGLYLDVTGLGCYHKKDVPQWYDGLTEQQRWDVQGRFWEAVAKRCAGSPAIFCYDLMNEPVVGGRDKR
TDWLGPAFAGKHFVQFIALEQGERERWAVAVQWIRQLSAAIRRRDKRHLITVGLVPWSLPGKGLTSGF
EPQRIAGELDFVSVHIYPEKDKTAEAMETLKGFCVGKPVVIEEIFPLKCSIGELERFFTESREYACGW
IGFYWGKTPEEYRHRGRIGDMLTLAWLQFFQTQANRTGGQTLNPKQSQDTNIQMIKTTMGTMN*
>SOL_1_10_cov_5.168147_N_161732
MRSRTIVLVLLAGIISLFTGYVPAQTNYSSAGVAKPAQLIFNSSFEENNNGDNLPDHWTGTNLSSQDG
WVCDQAHLGECSVHLVSSSTTGKKIMFTRNIDGNKDEKITFRLWNKTMNAGEDFRAQLTITYWDQTTQ
AWNIKPQSGSHGWQAAQVLVNPTKPYIRIDVQLISPANTGGQIWFDNVNLNVAAVPGGWTPPKTAGEW
KNHPVRGAVIEIGFGGHEEYEYPPIPNRNSLRLLRELGVHVVVLEFQFAWTIRPPYKPVEAQYALVTK
ALDNIRDAGLYAVLSVRNGPGTNAMIPGIADPNVITTLYTNTTAQQAYRNMLKDVVTRFKGRKEIIAW
EPLVEPCLDHFLYHEESPFPKASAKWNPLAASFIQTIRAVDPDRPIIIEPVNWGGMGGFIKLAKFNDN
NVIYSLHTYEPYDYTFQDPPYKSYPGIFSGEYVNKSRLDNWLAPVDNFQNKHDVPILAGEWGGTRWLP
GMNTYITHQTSLFDKRDWSWTIYAWYDCAWDEKGLILYLGSKRYQPVYNPFNPLFLPIVNSWASL
>SOL_1_10_cov_11.057440_N_169795
MFKYSKRFARWWFAATVTFLMWGVVVAACREDLEWVKVSQDKKGFVCEESGERFVPLGFNYDHDDEGR
LLEDYWHLEWRTVEEDFLEMKRLGANVVRIHLQVGRFMREPNEPNEASLERLGRIVALAERVGLYLDV
TGLGCYHKKDVPQWYDGLTEQQRWDVQGRFWEAVAKRCAGSPAIFCYDLMNEPVVGGRDKRTDWLGPA
FAGKHFVQFIALEQGERERWAVAVQWIAKLSAAIRKCDKRHLITAGLVPWSLPGKGLTSGFEPQRIAA
ELDFVSVHIYPEQDKAAEAMETLRGFCVGKPVVIEEIFPLKCSIGELERFFTESREYACGWIGFYWGK
TPEEYRHRGRIADMLTLGWLEFFQRQANRTGGQTLNPKH*
>SOL_1_10_cov_6.004515_N_174552
MTIENLQFGQVPSPFVKRALTIAVMFAAALLPDGGRAAERVKPSRSGPDRMQLIVVAPDGQGFVERDS
GRPFIAFGTNYYDPDTGWAPKIWREFNAQRVRQHFGVMSELGVNCARVFLTAGSFQPNPQRVEEQALE

KLDELVKIARETGIRLILTGPDHWEGVPSYWQPDRFAGKPALDALQRFWDIVGRRYKGEPAIFAWDLL
NEPEAPWFVEHWRPQWNAWLQKTYRSWDELKAAWGTELTEADRWDEVAVPKNQPDLRNPRLRDWQRFR
EHLADEWVRCQVEALRRADPTHLITIGYIQWSYPLIRSGPPGRYAAFNPRWQARWLDFVSIHFYPTMD
SPFSSEQSWQNNLAYLQAVLAYCRTSKPVVLGEYGWYGGGAPQQHPYLGETQQANWIQAEIEASRALA
DGWLSWPFADTPESTDISLYAGLVKSDLTVKNWGRKFKELAANLSELKSAKGGTLQLPAFDFPQAFTA
GSEDLDKMHQTYVSAIQRPLGRTFNRGTERD*
>SOL_1_10_cov_2.383327_N_181721
MLLMIGTMTIACKKKEATKPVDQGGTESPQALHCEGRWILNEDGAKVILRGVNIASLEWSVSGENMLR
SLDSAVVGWHANLVRIPLSQDRWFGKTSNQKDDGQSYQSLVDRLVQSSTEKGCYILLELHWNDAGIWG
QNIGQHKMPDSNSVIFHRSLAAKYGNVPAVLLGLYNEPHDISWDVWLNGGLVTEDSEQ
>SOL_1_10_cov_11.029379_N_183050
MKNARLIRTRFVKQGWWLSTAVVACVWAFNGTLAQAKDSNHAGTQFDIRRGTNISHWLSQSGRRGEDR
QSWFTEKDVKYIASLGFDHIRLPADEEQLWDERGNKEEEAFELLHSAINWCKDNKLRVVVDLHILRSH
HFNAKDKPLWTDPNAQERFLQLWRELSADLKKYPVALVAYELMNEPVADDAEDWNELVEKAIREIRKT
EPNRNIVVGSNKWQSVDTFDKLRIPQGDRNIILSFHFYTPMLITHYKASWTGVGKYTGPVNYPGQIVT
DKDMEGLSEQMAQLAKRNNGVYNREVFAKLIQKPLTLARKYDLPLYCGEWGALPTIPREARMRWYADV
RSVLEENNIAWANWDYKGGFGIIGSGGQGDEGFIKVLIGSSAAKDR*
>SOL_1_10_cov_4.590133_N_188182
MRSKKACEICFWLLLAGILASSPAVSQAADRQPDGQCFVRISPRDSRYFELTNGRSFVPVGLNMIAPP
GGNEEQALARMEQWIRSLSENRGNYIRLWLSNNFFDVEHERSGQYDAEKAKRIDAVLAMARRHNIRVK
ITIEHFRHFFGKTQSWAAKPLHHVSRGGPAEDVDDFFQGQRSREQFKKKLAWYANRYGSDPIIFAWEL
WNEMDTVRGKGYMEWTEEMLAELKRLFPNNLVTQSLGSFDNDGKRQRYRRLCLMADNDFANVHRYLDL
GASLDVCKGPVDIMAANAIQELQNFKPAKPILLAESGAVEPSHSGPFKLYQKDKAGIILHDVIFAPFF
AGAAGTGQTWHWDQYVAANNLWFQFNRFAEAVKDIDPAAEAFEPAFIDHPRLRIYTLKGRHTLLAWCR
DKQNTWQTELAESKPPTMVDSAVVSLPASNQAAVSFYDPWSNKWIQGEAKGNKITLPDFTRSLVIKMR
Y*
>SOL_1_10_cov_4.315242_N_190258
MPFPVRRGTNISHWLSQSKARGQERRAWFTRADVQRLAGVGLDHLRIPVDEEQMWDEAGHRDREAFDL
LDAALDWCKEANLRAIIDLHILRTHYFITETEPRLFTDPAEADRFAGLWRDLSAHLRRRETAWVAYEL
LNEPVATDPADWNRVARAAYDAVRADEPQRVIVLGSNRWNSTSTFPHLDVPAGDPRLLLTFHFYNPML
ITHYRAPWTPVGRYEGPVHYPGQPIKPEDWGGLAPDLKALLGAQNVFYNGSVMERDLAPALAVQRRTH
LVLYCGEFGVHHAAPDPVRIAWYRDFRGVLARHGIAWANWDYKGSFGLFDAKGNPTAVVEGLLK*
>SOL_1_10_cov_12.479375_N_196704
TTRKPVPLNKNRDTTMNHQMTHSTARYLTTAPSKLARLDSTRAATSLLLLCLLNLPCLAAATLPELKI
PDGFGVNIHFTGDPKDLDLIRDGGFKFIRMDLGWGGIEREKGNYNFERTGYDALTEGCSERGIRILYI
LDYSNHLYESDQSVRTEEGRKAFAAFAEAAATRYKGKGILWEIWNEPNIKQFWTPQPGADDYCRLVEA
AAPLVKQADPSGLVVAPATSTIPFDWLEACFKNGLLKWIDVLSVHPYRPQPPETVIKDYDRLRELIKR
YAPQGKDIPIISGEWGYSNINWDKARLSEEIQARFLAREFLINLYQKVPVSIWYDWKNDGTDPNEREH
HFGTVMHDLAPKAAYLAAKTLSSTLPGYSIDSRLDLAGDKDFAFKLINGNNQAIAIWTMADDHQVTLQ
TPSGKGALIDMLAKRQEISWQENALTLTISQSPQYLLIDAK*
>SOL_1_10_cov_4.782927_N_199120
MKKPVSAKHVSVAAACLSLICVLGAAPAKEAGFRISRGVNISHWLSQSSTRGQRRLDYFTEKDVAYIA
GLGYDHIRLPVDEEQLWDEGGNREAEAFVLLNNAVKWCSDHHLRVVVDLHILRSHHFNAKDKPLWTDP
KAQDKFFQLWRDLSAELRKYPVDLVAYELMNEPVADDPEDWNKLVEKAVKEIRKTEPQRRIVIGSNKW
QSVNTMDQLRIPQGDRNIILSFHFYIPMLITHYKASWTDVGKYTGPVNYPGRIVDPNQIERLPADVAR
IVKQNNGVYNRDALEALIRKAIATAARYDLPLYCGEWGCLSTTPRQARLQWYEDVRSILEANNIAWAN
WDYRGGFGIVGRDRQPDEGLIKALIPK*
>SOL_1_10_cov_3.333191_N_207146
MNRRDWLKAASVAGGAALLGVAPGGAEGAAAVQAAQGKAARDLARKLPRWRGFNLLEKFTQRGNKPFV
ESDFEWTAGWGFDFVRLPMDYRCWTDAADPYKYDEKVLKEIDQAVEFGRRHKVHVSLNLHRAPGFCIN
PPAEALNLWTDEESQKQFDAQWSMFAKRYKGRPAAEVSFDLLNEPARYPSLDRYVSVCRRAVGAIRRA
DPDRLIIADGGNVGQQPVLDLVDLGIAQSMHAYMPAQVTHYKASWVGGSDKWPEPTWPLKQKDKMLDR
QWVADTRIKPWKELADRGVGVNVGEWGCFNKTPHAVVLAWARDVGGLVKEAGWGWALWNLRGSFGIGD
SGRADVQYEDFKGRKLDRKFLDLLREC*

>SOL_1_10_cov_5.602482_N_207912
DYLQAVLEVAGRFGFLLFVDPHQDVWSRFSGGDGAPGWTLEAVGFDLSRLDQTGAAVTHQALQDRYPW
LVWPTNAEKLAASTMFTLFFAGNDFAPRTRIEGEPAQEYLQRHYIQSVCQVAARCRGMAHVLGYDTMN
EPGQGWIGAEDLRQVSGVLRKGASPSPWQSILLGAGFPQEVEEWAEGRSLPRRKGARRVNAEGVRVWR
EGFDCPWKANGVWDLDAVGRPRLLAPGYFARIGDRPVHFTEDYYKPFANRFAQAIRQVHPQALIFLET
FTEGRDELEWGPEDAPEVVFAPHWYDIAILVTQHFTSWAAIDIFKKRVVLGPWAVRRSIAGQLARYQE
TTRRQMGGIPVLFGEIGIPLSLDNGRAYASGCFRQQEQAADRSLRAVEDTLAHCTVWNYTADNRNQHG
DQWNEEDFSLFSLDQQKDPADLDSGGRALDAFIRPYPKAVAGEAISLRFDYRRHRLLFTFRHDPAVTS
PTEIFLPRRHFPSGALIQVSDGRWEEDREGQLLRYWPGREQMIHTLRVAPAVAR*
>SOL_1_10_cov_3.511566_N_215183
LYAEQENSAQRWSEEKAWQWSKEQGWLCGFNYVPANAISYTEMWMGYAFDPNLIDSELALAEKTGFNC
LRVVLPFVVWQGEPQAFKQRLDTFLGICSRHGIKAMIIFFDDCVFGPIEDPVFGKQPDVVPGWYANGW
TPSPGHSIVKDSSQWPKLEKYVKDILTAFKDDRRILCWDLYNEPTNSGMGEVSVPLVEDVFKWARQVN
PCQPLTTGRWNNYEKLNEVIFANADIISFHNYRPAKELKAEIEELKKHNRPIICTEWLNRPNGSVVSE
CLPVLFEENAGCMHWGLVNGKTQTNLPWGHRPGDPEPKVWQHDLYHSDLRPYDPNELKMFRKYLEAGK
SRTDKAAKEQCNEKPV*
>SOL_1_10_cov_3.752144_N_218034
EAVVGYDLMNEPFVGSLAPQSMFMIVAKFAEVMAARQGPDAPNAMELMSQWGTDEGRAKILKLLTDPQ
VYGQVVDAAQPLFQEFEKTRLTPMFQRVTNAIRREDKRHIIFLETSMSSNMGIRSGIEPVADPNGQRD
PQQAYAPHAYDLVTDTPGVDAPSNERVEFILARHAETAKRLGMPALVGEWGAYGGAKALAGAQFVCRQ
FEKLLFNDTYWTYIEHIEDTPAFPALVRPYPMAVCGTILEYGAEPQARKFTCRWKEDPAAKADSRVFI
PEAFGPSQDRVKISPPGKGFTIQPVRNGSKSVYVIIPPAGGESERRLSLE*
>SOL_1_10_cov_3.866511_N_225228
MKIAAGVLVCCLVCGYSAAQEQPKPQAAALTVGKDGVIVRDGKPYRGIGINYFSAFTRTLANPEDTSY
REGFAELAKRGIPFVRFSACGFWPSNWKLYREDKEKYFRLFDGVVKCAEEKGVGLIPSLFWHLPCVPD
LVDESCNQWGNPNSKTIAFMRTYTAEVVQRYKDSPAIWAWELGNEYSLAADLPNAADHRPPVYPNLGT
RAARSTADDMTHDMIVGACRLFAEEVRKYDKQRPITTGHSLPRASAHHQRVEKSWSADSKDELQANLI
DVTPDPMDLISIHVYPMDEKGRFNQEKTSYEELLTLCVQAAAKAGKALXXXXIRRERYREGRRP*
>SOL_1_10_cov_4.015031_N_225712
MKKSKGVSRRAFLCASGAAVAACAMAAASESEVTAPSSSKLPKWHGFNLLEKFMVHQNARXXXXNDFA
YMSELGFDFARLPMDYRCWTDPNDWTKLREDVLKEIDEAVSFGEKHRIHVCLNFHRAPGFTVAQPPEP
KELWSDDEALKVCAMHWARFAERYAGIPNDRLSFNLFNEPTLVSGQVHRRVVARVLEEMRKHDEKRLV
ICDGRFWGRMPPEELLGLGVAAATRGYDPMPLTHYKAEWVSGSDKYDVPAYPLKEGDTLWNKERLREK
MIVPWKALEAKGMGVIVGEFGAYNKTPHPVVLSWMRDVLDLWKEAGWGWSMWNYRGAFGIVNSDRADV
TYKDWHGYKLDSLMLTVLKSAM*
>SOL_1_10_cov_3.228459_N_235187
MKRIFPLFLSLALSVNAADAPFHKGVNLTGWLQTSGARQIQFTKFTRQDFVNIQSLGCDVVRLPINLH
FMTNGAPDYTIDPLFFTFLDPIVDWAEDLGIHLILDNHTFDPAANTSDNVGDALNPVWTQMASHFKDR
SVLVYYEILNEPHGITDAKWNAIQQRVIDAIRAVDTKHTIVVGPSGWNSYNNLALMPRYTDANLIYTF
HFYDPFLFTHQGASWPSPSLAPLAGVPFPYVAARMPACPAALKGTWIEGSLSGGYRTEGTVKHLKDLI
DIAVRFRTQRNVPLFCGEFGVYIPNSPAGDRVVWYDAVRSYLEEKGIAWTIWD
>SOL_1_10_cov_3.639041_N_237930
MNYSGLKIPLSNLLTLILICGFFSRTDAVLAYYTTRGQDIVDRKTGEKVILRGFGLGCWLLPEGYMWG
IRKLDRPRQFEKAIEDLIGKEKAAEFWRRYHENYVTEDDIRVMKSWGVNSVRIPLLASMLQPPEEQPD
RPPFRYSDEGFRFLDSLVAWCSRHRVGVIWDMHGAPGAQNAENISDSDGEARLWTEKGKYWPRCKDLW
FQIAKRYQG
>SOL_1_10_cov_2.828396_N_242768
MPFIHRNRILLLCCLFMLVVIQCRKKSPTQPAIQTPKYTTFKVEGRHLVDPYGEKVVLRGVNAMIIYW
DKLGSVTYPEIAKTGANVVRIFWMDDPVANAVDLDGNLLTCERNHMIPLPGVWMATGKWDQLGACVDY
WCRPDIAAVVKKHEKTVLVNIANEAGDGGVTDAQYRAGYEDAVSRIRAAGIRSPLVIDAAGWGRGEKY
ILNNAQYLIDRDPERNLIFSWHPWDPISWGGTKMRIRATIDSAVARNIPFIIGEFSRSEQADSRSATT
PIEWRTIMEYGHLNEIGWLPWVWWCCEEPNDGHSLTRDKIFGHWANAPWGEEVAVSGTYSIQKTSVRP
ESMQ*
>SOL_1_10_cov_4.789916_N_263326

MNSQSQVDKDGILVSGPWFKDNHGRNLLLRGVNLGGSSKIPCHPDGATYNSKSLLNHRQVSFVGRPFP
LDEADEHFNRLRKWGLTFLRFVVTWEAVEHAGPGIYDQDYLDYIHAIVEKAAKNCINLIIDFHQDVWS
RFSGGDGAPGWTLEAIGMNITNIHCTGAAFLHQNNTGPLPRLIWVTNSTKLAAATMFTLFFGGNYFAP
ETKIDGEPVQEYLQRHYIESIKQIAIRLKGLPNVVGYGTMNEPLCGYIGWRDLNKPRAVVEVGAIPSP
FQSMLLGAGIPQEIDVWERRITGPKL
>SOL_1_10_cov_3.829211_N_275150
MKKYFLLLRALNCLHLIDQKDCKHYGKCRCPVTSKALSVVILLSLLPFVPLDAGTKKAGPVPDNASAP
LKWLHAEGGAIVDESGETRILLGVNRSGLEYNKKGNKISEEEIRFICEDWKAQVIRLPFNEEWILTDC
AYNRFFDRVIGWINQNGAYAMLDLQWQDVKVRIPPIPDEKAAGMWAILAERYRNNPGVLYDIHNEAHD
TTWNAWRGRATAIITAVRSKHPKSLIFVSGLDWAYDLRGWAEAPLPFADIVYSTHPYPFKWEPWAWDK
YFGDFIGKVPVFAGEFGGGEKDLEWGRQLIDYFDRKQIGWAAWSWVDQPHLTQKDRRTPTPFGDLVKA
ALLKHAGMKPDSTQKR*
>SOL_1_10_cov_3.751807_N_280784
MRKSAFILLAFLIVNGAHAGTQASADIRVNQIGYYPNAPKVAAVIGALAGNFAVLNSATGDTVFRGAL
SAEKKWPHSDEQVKLADFSAFTSEGTFILSVPGLGDSHPFDVKPFVHQAVAAASLKAYYFARASMELT
ETFAGPWKRKMGHPDTKVRVHASAATASRPVNTLIASPWGWYDAGDYNKYIVNSGITMHTLLLLYEQF
PEYCRDLKVRIPADGYGLPDILSEALWNLRWMLTMQDPDDGGVYHKCTNENFDGFVMPAQATNPRYVV
QKSTAATFDFAAVMAQACR
>SOL_1_10_cov_4.292085_N_291676
YFSLAGQRFVPVGANYWPGSCGVEQWQRWPVDEMQRDLDLVVSLGFNSVRFFLRWQDFEPQAGQYEPV
MFERLRQFLTWCAERGLYAHPSLFVGWMSGGTFWPEWKGERNLFADPFMVECAAAFARQAAAVIAPFH
RHVLAIDQGNEICCLSDSPAAPPHAVIEWCRTVNEAVRSVYPHCLILSGNEQNQILNDTGWRFGDQPG
TDLYSMHGYPVPAWHSVGFDGMTDPLCQSLLPFYTQVARAFGPVMVQEFGTIVTFGPRQQDSYLRAVL
PACWEAGANGFLWWCLRDITAEVHPYRTQRFESTLGLVDARGKVKPGLEYWIEFAHMLRERPAPVSGG
EEIGLYWPRHFYPRENPQNPGNAPRLLSRWLTMANFVLRQLGYAVRIVRGDLPLDPGLHTIVIPGALL
DLGEVNALDAWVRSGHTLIWHGPDPVNWGHEYIALLGARPVDYRADRPATVELFGNVWTLDTFPRSMR
VELVPDGATTLAADQDGLPTVLAHRAGQGKVVYALPVVESSIADDAGDREARSRWQRWYAGMLQA*
>SOL_1_10_cov_3.466075_N_294185
MKRLPSLWIFLFVMALACQTITGKVTQSTQVLAPTRSMPPTQVPVATHISPPTKITASTSAPSTTPGE
ETTTLNKWSLWVDGPHLRGANIHQRRIYPELDGPTYMGPGPLGAPFTQVDFNRLAELGANYVNISHPG
LFSEKPPFTLDENVQQNLDRLLEMIAKADMFAVISFRTGPGRSEFTFYWGTQGDWFTEDYYNDTVWED
EPAQEAWVAMWRYTAERYKGNPVVVGYDLMVEPNSNDVWFNEWDPETFYAQHGSSSYDWNQLAQRISD
GIRQADAQTPILIGGNAYSAINWLPYVKVTGDARTVYLVHQYSPGQYTAQEPGEEGYTYPGEFDIDGN
GSPEAFNRVWLEKTLFSVIEGFTSQHAVPVAANEYGVRRWAPGASDYLRD
>SOL_1_10_cov_3.429487_N_297098
MKDLLRPVSWFMILFIALLISCSRVSPQQFNSARMDPISIAKDGRGFILTPSGKPFVPWGLNYGNRGR
LIEDYWESEWQTVVDDFREMKAVGANVVRVHLQFGKFTKSPEKADESALRQLGRLVELAEQTGLYLDL
TGLGCYRKADIPAWYDVLSERERWSTQARFWEAVATQCSHSPAIFCYDLMNEPLAPAGKRKPREWYSG
TLFGGYDFLQWIALDQGNRARDEIACRWIDTLTKAIRRHDLQHLITVGLLPSSPQLGHFSGFLPEKVA
PHLDFISVHIYPENGKVEQALKTLHEFSAGKPVVIEETFPLSCGRDELEVFLRASRGIACGWMGHYDG
QSPEELEQLKQQNKITIPQAIYLDWLQLFQKLKPEMLVK*
>SOL_1_10_cov_3.599612_N_299541
LTRGVKIFAGISILAAVCLLGFPAAAPAAAWEMRTTDDGGVEILHERATVVTATYVFWGANWSWVHFD
SRLGTPDAGGAVPLAARADALKLKVDGTVASPSPNTLVYTYQLDAAEERKGIIGGGVEFVLGLDSASF
ATPPGEPVLLEDNRGWKWPAAQGQAVTVAFDPPLASVYFERGQKRQIRAMMVGADLAKGTTKIVMTVT
LPEGGRVVKSAAERYGPADTPTWHPDAMVWNESPVDVSFLSAEDKPAGRHGFVKAQGDGLVYEDGTPA
RFWGGNLAAYALFVDKDDIRRQARRIARLGFNLMRIHHHDSMTWVSRPVIDKTRPDSQTLDAEVMDRL
DWWIKCLRDEGVYVWLDLHVGRQFKEGDALGEGAEEILRHKGEIKGFGYFSPRVQELMKEFNARYLGH
VNPYTKVAYKDDPAVMGLLVTNENDLTHHFGNLMLGDKGNPVFNRIFLEKVREFCAKTGLAEGETGRT
WVPGPSKIFLNHQEHLFNQAMLGHLATLGVKVPVATTNFWGDENLYALPALAESGIR
>SOL_1_10_cov_3.522078_N_300603
MGLRQVIVAALGIVLISGSLQAGDGPAGFVERHGQLSVKGTQLVDAKGKPVVLAGMSYGWHNWWPQYW
NAETVRWLRDDWKCTVLRAAMGVEPDNGYLRKPDSSKALLKKVVDACIQNGIYVIIDWHDHNAHNHPD
AAVEFFAEMARAYGKIPNVIYEIFNEPAETDWKTVKAYSEKVIAAIRAIDPDNLILVGSPHWDQDVHV

AADGPITGFSNLLYTLHFYAGTHKAWLRERGDYALKKGLPLFVSEYGGCEASGDGPLDLNEWNLWMDW
MDANGISWCNWCIADKRETCSVLKPGAAASGGWSTKDLKESGTIARDLIREWNVREFPPDGANRQ*
>SOL_1_10_cov_2.096932_N_320235
RFGCNMVRLHHMDADWATPNIFDTSCDDTQHFSAESLDRLDYLIAQLKRRGIYIYLDLLVHRKFKAGD
GVRDWQQVENGAKIVAHYNPRIIELQQKYAHDLYTRVNKYTGLRYCDDPAIAMSEVINESSLFWAAGY
AEVPPSYVAELDGLRYRQPEALESRXXXXGGRGPKRARPRCPSVSL*
>SOL_1_10_cov_2.096932_N_320236
MVEGLSARDLDVLQFLYETQVRYFTSMRDYLRSIGVKVPIAGSNHWEAVPLDLQSNLAMDYLDRHGYW
DHPQNGYGPGARFENQPMVKSGSWDLVSWLAPQRAADKPFIVSEWNCCWINEYIAEGPLLMAAYGAFQ
GWDGILQFDYSGADWGERIDGNFDVGNKPHVFALWPAAARMFLRGDVRPGRALVQARWRTEDVGPVGD
QLPARAGLRRRLAYTIVKAGEPERGPRLTGPVEPAVTDTEELAWDAGAGLVTVNAPSSVARIGFASGP
VEVGPVTFEVTPDFAAVTVTALDRKPIRQSGRLLIAATARAENTGMTYGPGRKTAGSFR
>SOL_1_10_cov_2.886139_N_324398
MENNRRDFLKIVAAGAASAAVPGFAQEPQKNEKAGNTPVQKMLPRWRGFNLLDMFTMQSKGDFREDDF
RWMRDWGFDFVRLPMCYRLWIEDGDDYKLHEPMLEKLDRAVDLAGKYGLHVSLNFHRGPGYSVNREFD
EPHNLWKDAEPLKAFCFHWQMMAKRYRGISKDKLSFDLINEPPSIGDLMSRADHERVVRATVAAIREI
NPDRIVIADGMSYGNETAPELADLRIGQSTRAYQPMFISHYGASWVDSKDYPEPAWPGHGWDRNRMEQ
HYQKWADLAKMGVGVHCGEGGAYNRTPHDVVLAWLRDVLEILTAHGIGLSVWNLRGSFGIVDSDRKDV
KYEDFNGHKLDRKLLELLQEFA*
>SOL_1_10_cov_3.091619_N_325598
MRCLTQHCVVLMIALMLLIAADVTAVAEEQSFVRVSPRDARYFEYSDGHPYIPIGLNMCWPPWPGKEM
GVMEDWMKNLAANHGNFVRVWLSMTYFDIENTRSGQYEESQARHIDEFLAMARRHKIRVKLCLEHFRT
FEGKSSTTTKPLHLAANGGPTQTHRPQTGS
>SOL_1_10_cov_4.431670_N_330773
LVRDTVAAIRQVDGVHTLVISGTQWGNMQGLAFLEIPEGEENVICTFHYYEPFPFTHQGAGWVGEQYW
TIGVEWPGPPKTPLVPKLAAQEDPLVRRWFQDYNTQPAESNPCSPGEIAKQLDVAVAWGQKLGKPLWL
GEFGAYRTGDMQSRVNWTAAVRQAAEARGFSWAYWEFGDSFGVYDRALEEWHEGLLRALIPQ*
>SOL_1_10_cov_3.528426_N_333267
MFARRCKGIPSSEVSIDLVNEPDNKVAFEGYAKVVRRVAAAIRREDPDRLIIADGRSWGREPVFELVD
DRIAQSTRGYEPMQITHYKASWVGGGNWAEPAWPLKQKDKVLDRKWLYEDRIVPWKKLEARGVGIHVG
EWGAFNKTPHGAVLAWMTDCLSLWKDAGWGWALWNFRGAFGVLDSGRADVTYEDFRGHKLDRKMLDLL
RQS*
>SOL_1_10_cov_4.615328_N_336379
MLDTRSTVFSFKFLVLSFIVFAATSPLYVLRAEEPRMQLIIVAPGNQGFVERDSGRPYIPFGTNYYDP
HTGWAPKIWRQFDSGKVQEHFRIMRELGVNCARVFLAAAGFQPSVETVDEQYLKKLDALVEIARKSGI
RLMLTGPDHWEGWPSYWKPDRFAGETSLQALERFWDVVGRRYRDEPAIFAWDLLNEPHLPWFVEQWHP
LWNSWLKETYGSRESLKAAWGRELTDVEQWGQVAAAEDKPDINSPRLRDWQRFREHLADRWVRRQVEA
LRKADPTHLITVGYIQWSYPLVRHGSPSRYAAFNPHRQATLLDFVTIHFYPTLGDPFGSQNNWSGNLD
YLRAVLAYCHTGKPVVLGEYGWYGGGAARGQPYLTEGQQADWISAEIEASRGLAVGWLSWPLADTPES
TDMSIFGGLVKADLSVKIWGQKFKTFAADLPQLMQPAPK
>SOL_1_10_cov_3.474486_N_346412
MSASVLPRVLTMAVVLVGAAFAVCRADEAQIPFPWPKQMRGVNVILGRVTEGDLEHLANDWKANSIRL
MTGSFLANEPPYAINAERLKTLEEVVGWCRKHGLYCVINLGQPQQNRDAGWFWQTAAAQQKFVELWQT
IARTYAASGPGVAYDLMNEPHGSGSAEAWSRLVPELTSAIRQVDQVHPIVVEAPAWGNAEGFVDLKPL
STDANTVYSFHFYHPFDFTHQRGAAGTLQANPEDHPLGLRYPGRIKAFWKDAPIEDWNKATIRERVEP
VVKFRDTYKVPVWCGEFGCTRWAEGAPQYITDCLDTWEELGIGWAWYSYREWYAMDLEMGTEDRSRQA
PRYESDLVKLIKPYFARNLEVPGSLLTVVSGLFLTMRLINTPAMGNGWMVAMQALGMVGA
>SOL_1_10_cov_3.239329_N_346656
SSGMVGNRPSVLAAMCYPAYSGWPKAEGYFDTRYVWAHTEFTPQQTMRGKQAIYGYLYGLTKVDTLAF
NFDGLAAFCDSWLKVPGDAGYDSRANLYDDPAGIIDFYDFAVFANGW*
>SOL_1_10_cov_3.060079_N_358384
IGRKSENMDDKKRMDMVDKNNSSIFQTPKLVLSKFFPVMILTLTMVCAIQAKPADAFEQNNKLGRGVN
IIGYDPIWMSKDEGRFKEKHFKIIKEGGFNTVRINLHPFRHMDKNAPYRLSSFLFEITDWSVENALKN
GLMVIVDMHEYNSIGNDPEGNKERFLSFWRQIAQHFKDTPDNVIFEILNEPCRNLTPELWNQYYREAL

AIIRQSNPTRTVIIGPPFWNSVKHLNELKLPEDDRNIIVTVHYYLPMDFTHQGAPWADRKDKVGVKWL
GTEEEKQAIVGDFETVQNWAKKHNRPIFLGEFGAYDKGEMDSRVRYTAFVARTAERLGFSWAYWQFDS
DFIVYDVNREQWVEPIHNALIPQKQ*
>SOL_1_10_cov_3.344382_N_365520
MQNDRNSFSAGKKSFITLVFLCTYTLVLLCAAALPCLAGQLPAPVIPSGFGVNIHFTGEPKDLDLIAE
AGFKFIRMDFSWSGIEREKGVYNFQSTGYDALTEACIKRGISMLYILDYSNRLYESEQSVRTPEGRLA
FAAFAEAAAKRYAGKNILWEIWNEPNIKQFWQPQ
>SOL_1_10_cov_3.601467_N_368204
RSWNGVKFMVCVDTPGGKLWPNATTETGTFDWRRATLVARIPPDATAVTLFLGLEAVTGKAWFDDVKL
YVGKPPPAATARPVVTTGLARDVPRLRGAMISPDINEAGLRTLGKEWNANVLRWQLIRYVGQGKPTPP
PAEYDAWLEGELKRLDAALPLCEQYGLYVVVDLHSPPGGSAKRTGYLGSDDRLFTEKASQDKFVAVWE
KMARRYKGARPVWGYDLANEPVEEAVGDDCLDWRDLAERAARAIRAIDPDRAVIVEPADWGGPDGLRD
FALLDVPNVVYSVHMYIPSQFTHQGVFAPSKPVPYPGEIEGRMWDRARLEAALKPVVDFQKACGARIY
IGEFSAIRWAPDAGAQRYLKDVIDIFEANGWDWTYHAFREWQGWSVEHGTEKDDTKPAAAPTDRQKLL
TDWFAKNRKPAWMSGP*
>SOL_1_10_cov_3.433805_N_375465
RRRAWSDLLGMINTRTFIITLTALLLAPLAASALAEGGPTNVVIRDEQGREIVPGGYVTITEDRQGTI
QYIPDDYRRMVRMGANFQVIRTALGRLGGWPGKEADSTYFGQLDAMVRMGREAGLKTVFKLVVYDLRP
FGNEQWDALWRNSGGSQDALMAAWNKVWIRYKDEPSVFGYDLLNEPQRGLDADYERCCREHLLPTLRR
LADAMHAVSPGKWALYQPLFLNQEDRVKGVNPFVPMKEPFGRDRVIYVPHLYHMDLALMARTLDAYAR
EAALSRAPLLIGEWGPATPLAADASPERQARYTKVYQATANALDQRGIGAIKAWFCGTRTPLTSKAQP
APFTWAIFSDTDAVGHVERKYITDALARPRPLAVAGRIERYGYDFAARTLEVSLKPDAGLGGTVIFVP
MDRFYPRSEE
>SOL_1_10_cov_3.432981_N_395531
WAKMLRYYPVDASEIVTTEGEGGDLRCTIVYHYLGAFLNSKGCIAPVPMLASFGLQHKMPGLAIEKAK
DTGYRSPYAPYLVVENTNTVTYRAPAIDRTKVLKGVGELFHSRREEDWRRMADWGFDHCRYAWAFHAD
WDLPLVKYVGGPLIENNDATWKKLDAEVEKCNKAGLQMMLTWFFNEDQPQRDTGGAVRNSTRYWRARP
EAQGNAFALWRRLAERYKDKPEWAVAYDFFNEPAYMNADHWNAVMKELTKVIRSVDKKHWIVWEPGDG
WAQPQWCLWMEPVKDDKVLYSFHHYGKHWGYAYDEYYPSYKATAERTQVDPWLEAILFSIRHHVPIHC
GEFGISMIQPNGDGEAWLNDYLAMFERFGIGWNWWNYSGRDIYRTGLCAGDRTSPY
>SOL_1_10_cov_2.266014_N_398855
MLIFVEMLILVILGTLLLWGLSVVINFIARLQAFPTKIYYFKDEHGRICIYRGVXXXXRGIYNDDYIK
GILEDFKELAVEDIKVIVDIHQDLYGQKFNGNGFPEWTLQDDGRPFVAQKRWDRNYLQPAVRRCFRNF
WRSEDLKKRYREMISYLNRSFKDINSILGIDIINEPFPSTLNALSFERGTLSKFYESISVDHYTKELS
LFFEPWIGTSAGLPTYIRYSIPTHNLIYSTHYYDPLGGKYGSLGKWFMRRTFRMRAAESQTFRSPIIF
GEFGTPVGTE
>SOL_1_10_cov_3.919858_N_399010
RPAATKIAVAAGDQAISADLALDICDAKSGKAVWSLKDHAGALKPFKNGQKDGESGDFTAHLDFTDLK
VPGRYYVAVKGGGGTERSYLFSIAEDVYRAPGLAAWKALYYNRADTEKPEKYAGPWNHKADHRGPNQA
TEARVYRWVKNAHWDPVGTEIADPAPHDVSGAWWDAGNFDKYMGNTTLCHNELLLGVELFGAAPKDGD
LNIPESGNGIPDVLDEIRCATEWFLRMGDATGAAWGRVYEKTGCPPEADATPVMLTQQTSGATMNRAA
ALAWASTVWQERKLDPAFAKKCMDESMKSWQLLEARPHPWPADAKDPKKPAYTGEWFFADFQKCRALA
AACYFRATGKPEYEKIVQESASKWSIPPGENVEVWPAI*
>SOL_1_10_cov_6.217352_N_408272
AGTDPERVFFEVMNEPVIPDAARWNAVQKQVLAAMRASAPRHTLIATGPRWSSVDELGKVEPVADPNV
IYNFHLYEPHNFTHQGATWGADFWRHFKNVPYPADPEAVEKILPGVADERAKQILRRYGEERWNADKI
REMVVRAADWGKKHGVRLTCNEFGVYRKVAPTEARLRWIRDVRTALEKHHIGWAMWDYAGGFSVVVEK
DGRRAPDPATLDALGLKTPV*
>SOL_1_10_cov_6.652612_N_409636
MRWMRSVAFLAIVCVGLWVAAKANGASPSEGVSDARFARLARGINLSHWFAQSPRGDYGLQHLRTYNT
ERDLDLIARMGFSHVRLTLNPKAVSDAPEGLPFNAERLALLDAAIAGFLARNVAVVVDLHPDDDFKTP
LAKDDAAVERFVAFWRALAAHLAGTDPERVFFEVMNEPVIADAARWNAVQKQVLAAMRASAPRHTLIA
GGPLWSGPDQLVRMEVVADRNVVYNFHCYEPFRFTHQGASWAGDWVKGLKNAPYPSSPEAVAGVLADL
PDEKARENMIQYGKENWNAEKIDAVIARAAAWGKKKGVPLTCNEFGVYRTAPAADRNRCIEDMRKALE

KYNIGWAMWDYAGGFSVVVEKDGRRAPDPA
>SOL_1_10_cov_4.816557_N_419815
LCLHRAPGYCVNAPKEPLDLWADGSSGQEARRQFAAQWRMFAARYRGIPSAELSFNLVNEPPNVTADQ
YVRVAAAAVSAIWQGDLDRLIIADGADYGMRPVPELAPLRIAQSTRGYTPMLVTHYRASWVDGSDAWP
VPTWPILASISQYLYGDEKPELKSPLVLKTDLIRATEMTIKVHQVSHQARLMVQADGVTVLEQWLQPG
PGRGEWQESTFKPEWGIYQAVYDKAYTATLPAHTREIRFEVTTGDWLTFSEIRLSPYPGAPGGQLVLR
PGEAGWGLTQEAFTVDAQGKLAPVSGRMRYSRETLWTDWVEPWKAFAATGVGVHVGEWGAYSYTPHIV
VLAWMSDCLRNWQQAGMGWALWNLRGGFGLLD
>SOL_1_10_cov_2.703598_N_422553
RGVNLGGSSKVPFIPNGATHLGGGFLDHRGISFVGRPFPLDEADEHLARLHEWGFTFLRLLVPWEAVE
HAGPGIYDEEYLDYLRAVVGKANAFGMSICVDPHQDMWSRFSGGDGAPGWTLEAAGFDLARLDETGAA
ITHQVHGDPFPRMTWPTNGGKLAAATMFTLFFGGDDFAPRALIHGERAQGFLQRHYIGAIARVAERLA
DMPNVIGYGTMNEPLTGYIGCTDLNRAWGQITLGDGPTPFQGMSLGAGVPQEVGVWRLGRASMRRTGS
RLLNGARVRVWRAGEDCPWRRNGVWDFGNSGEPVLLRPDHFARVNGRGIDFARDYFRPFVNRFAREVR
SVDPRAVIFLEAEAGRWPPTWGAGDAPGVV
>SOL_1_10_cov_1.770669_N_437897
RILSIIRKTNPTRIVIFQGHNWGGSDELRTAAIPDDDYVIGSFHSYDPYLFGLEAQGTWGTSNDINTL
KNKFATVKQWSDNNDIPVFLGEFGSHRSCDYNSRMKHYKYYVGFAQAYGFAYCAWDDGGNFRILERAA
KRWDEVKDILLHTSDKSPAIPKLSLFQXXXXTCFPEG*
>SOL_1_10_cov_2.128999_N_457688
FEDGHDYYYTNFLRPIVDYCALKGIYAIIDWHYVGDNTYDRMTETTAFWEYMAPRFAGDSHVLFELFN
EPLNTSEGSESANWARLKTDMQTWIDLIRPYAPNNLILVGGPSWSQQIGPAASNPFSTSNPNNTNLVM
VIHIYPGHWLVYGQDYYKNQVTTCITRYPVFATEWGFWGTSEELLDGTITNFGQPLMDFYEPLKISHS
AWCAAYAWNPPMFTSSWTLRVGEAEMGGFVKDMLYAKRNSDQPGGGDTNAPAAPAGLSATGGEQMVSL
NWNNNTEPDLAGYNVYRSTTSGSGYVKLNVSLLTSSDYIDNDVDGYVTYYYVVTAVDTSLNESADSSE
>SOL_1_10_cov_1.786307_N_459931
PFLTQECRGPALQAHAAAEPADGFTNPDGICTGEYTDPGGDSDDRAWAAAELYRTTGAAEYHTAFETY
WAQNSPLWGWNDWQHHQQKASWAYVNTGWPDVNPTWQQQIQDAFVSDAETYLSRTQTNAYHNGARLDV
PVWIGWGAFTQSTRYAFRLLQAYALTGDNRYWDAALTNLNTQLGANPLSLSFVTGIGFRYPQDPLHNP
TMYDGIAEPVPGIPVFGVMAAMSKANPYYAAVQDDANSYPYAADERDPYPILRRYVDTNEIVPNSEFT
IQEMAWTAGVLGLFACSLNGDINDDGQVDVTDVMAVAADWHSPDFDPAHDLDDDGDVDIVDIMLVAV
>SOL_1_10_cov_2.060127_N_467370
MKKYLILFLSINLAGCGTIMKNFKRPHMDQMTYIRDHQNRICIYHGVNICNYSKHSPDFLPWHTEEDI
ARMNKWGFNLVRLLVFWQAIEPAKGQYNEAYLDSIVTRIDILKKYDIDVFIDVHQDVYGPKFDGNGFP
SWTLDGVKLPEFKKQQPWNLNYTQPAVIGAFQHFWKSPDLKQRYVEMLKKLMSRIDP
>SOL_1_10_cov_1.936709_N_467384
HKPIWISEMNWNVAPEDVEPRYGRVTLEQQARYAVLAYQRAQEEWPWVGVINFWYFKRADDTWERNRQ
PEAYFRMVGSDFEPLPVYAAMQAYIHSQR*
>SOL_1_150_cov_11.195680_N_5292
MVESLPIWYGFNVLEKFYRNDPFDEVDFKRAAEWGFNFFRLPMDYHCWILNEDPYKIDEKTLVEIDDA
VALGKKYGIHLNLCFHRAPGFSVNQSVKEPFDLWRDEEAQAACEFHWRLFAKRYKGLSNKVVSFNLVN
EPYGATMPVYKKFVERMLEGIWEEDEKRLVIADGLIVPEGHYKPVTGIDNPLFGQSFHVYEPHWITHI
MAGWAGVWGTYDEQPEYPCEAPNLDKYIERFPPDSSQRRDLQRWKGAFVDRKWLENWMKPWFEFRKET
GSLIHCGELGTYAHKVPRQTQLSWFRDVLGILKENKVGWAQWNFRGPFGVINTGREEFHSEILPNGDR
LDRQMLEILRKNIAH*
>SOL_1_150_cov_20.427587_N_8651
MSQNPLHIDGCWFKDEQGRVVILRGVNVAGNSKVPPFIPFTDAALLDPLKEWGMNVIRLVLIWEAIEP
EPGKYNEGYIDAMETLVNAAGERGIYVILDMHQDMFSRYVNGGCGDGAPSWAIDPSIPQDEPSNDERC
IDWINGLNDKNVLRAFDSLYANANSIRDHYISMWAHIARRFGDHPAVIGYDLMNEPIGDEVSQLALLY
EDAGAAIRKVDPDGILFVEPSILTSFGAIYSQLPPLSLGNYAYAPHFYSASLLVTDIFSTSEADKSFA
DFNSKVKEFGGVPLLLGEFGMYPEKTKVSEYIADIYRRLDDCFYGGTQWDYCPGWSPVALDGWNRENY
SIIDDKGNIRRNFKVRGYAQRIAGIPQKLEVSDNRIYLEWENQPEVTAATLLYVPIDVMFKGARFDIV
EGPSVHCKLDVEHRCLACTASGRGTRTVEVKAR*
>SOL_1_150_cov_11.610046_N_12881

MKETRWKINELEYLEAQGLSILVFHNFYPEGKQGGIEIIQHGERVATNGDLRLEPAPGQWSRVPKAGK
REVDVDKGIIKVPLSFPEHDYSFVVRVEAEGDSILVSVDLDRPLPSEIEGKASFNLELFPPAYFGKTF
HLGSTFSVFPRQANGPMVTVPDGGLEPAPMARGSRLTVAAEDPLRRMVIEQVGGGEMTLLDGRNTVQN
GWFVVGSVLPPNRTKGALQWRITPNSVPEWRRTPVIAVSQVGYHPSQSKRAVIELDAGTQDFGEATLL
RVDSEKGLSKVFSAQPKKWGKFLHYNYAVFDFSHVHEEGMYVVQFESEFTPPFRISRDVYSNDVWQPT
LETYFPVQMCHMKVYDRYQVWHGACHLDDALQAPTGQAHFDGYKQGQTTDTPYSADQHIPGLNKGGWH
DAGDYDLAAGSQAQTTFVLALAGEEFNVETDQTTVNKDERLVLLHTPDGVPDIVQQVAHGVENLLSGY
RAAGHSFSGIIEREIEQYVHLGDGSLITDNKVYDPSLSPGEVSGERSGKLDDRWAFTNHDTAIEYKVI
TALAASSRILRGHEDDLAQECLETAIKSWEHEQTHSPVVQRSAYSYQHETQEIFAAVELLITTGEDKY
RQRLLELSSRITGNIERVGWAVARALPLIKDKKFAGDVKEAIKKFKSNLDETLAKNPFHVPAAESRFG
RVWGAGWEILEHAMEQYYLNKAYPELFDPENLFAAVHYVLGCHPGSNVSLVSGVGARSLTAAYGTNRA
DWSYIPGGVVSGTALIQPDFPELKDNFPFLWQQSENVIGGSATYIFCVLATQKLLKTKEN*
>SOL_1_150_cov_10.768442_N_13150
MGNSEIFRENQLLGRGVNILGYDPIWKKRSKARMKDEHFRLIKEAGFSNVRIPLHPFRDSGINEKYRV
TKSWFETLDWAIEQSLSEGLLPILDFHEFGAMGQDPLGNKKRFLATWKQIGERYKDYPDKVVFEILNE
PNKELTPELWNQFHGEALAVIRRTNPTRTVIIGPGYWNNIDYLSKLELLEDDRDIIVTVHYYKPMDFT
HQGASWAGLQDKVGVEWKATPEEKQAVINDFEKAQAWAKKHERPLFLGEFGAYDKADMDSRVRYISFV
ARQAEKMGWSWAYWQFDSDFIVYDIPNNRWIEPILNALISHKK*
>SOL_1_150_cov_38.760455_N_14809
MILMPFLLLAPFLAQSKGLEWVSVSQDNRAFVFMESGRRFVPWGFNYDHDRDGRLLEDYWEREWDTIE
QDFREMKQLGANVVRIHLQAGKFMNGPSEPNQAALDKLAQLVEVAEGLQLYLDVTGLGCYHKKDVPPW
YDQLSEQERWNVQAHCWEAIAQRCADSPAVFCYDLMNEPVVAGGKRTDWLGPAFAGKHFVQFIAIEQD
SRDRPAIAVQWIKELSSAIRKHDRRHLITVGLVPWSLEGKGLTSGFVPEKIIQDLDFISVHIYPEQSK
LPEAMATLAGFSVGKPVVIEEMFPLYCSMDELGRFIEESKKYAAGWIGFYWGKTPNEYRASSQIADAL
TLGWLEFFKRTRPGN*
>SOL_1_150_cov_10.628398_N_16191
MVKDYRNSHSKSSIILVAFMAIVIIASSALIYQIIMGGSRTNSSISVFGFSQGIVPLYGAIEVNINVT
AVIDNPFDPSEANVSVVFTAPSSQTIEVPAFYYQEYDRNLTGNREILTPAGAPFWKTRFTPTEVGEYS
FYAKLKKEAQTETTDVFTFDVASSSSRGFVRVSGVDRRFFRFDDGSSFFFVGHDVCWSGSRGTFDYDE
WFSSMNQSGETITRIWMAPWVFGIEWTELGNYNLAEAWRLDYVLKKAQEKGIYVLLCFMNHGQLQAAE
STAQWKDNPYNKANGGPLEKPEDFWTNGEAVELFKKRLKYIVSRWGYSTNVLAWELWNEVELTDNYDF
VKVSEWHNDMAEFVRNNDPYGHLITTSSDSRFGSLQSLDLLTVHRYGPTGFLDISGAAHDLISDLIQQ
YQKPVILAEFGADWRWSDDSYTTKDAEGVQIHNGIWSSVHSGSASSAMLWWWDTYIHPNNLYYHFEAL
SRYLEGIKPDEAELKALEVQFVQPAQINVEDLCNLTIYPSLGWSRPEANVFEVDLYGNVGNASQLSGY
IHGVFHPDLRNNSTFIVNFTYGGEVVVHVNSVANSGAALKIFVDGSLMKTVNLTDIDGKNDGFVNEYN
LDVSVPVPAGRHEVKLDNGGNDWFTVDYVTFTKAVLKCSKARVMGLCNDSFAMVWVQNKEHTWWNVVN
QMPIEPLESVELELLGFQDGTYTVEWWDTYTGEIVRTEFIQAVGGKIPLHIETLEKDVAIKLTILTIV
SETSMG*
>SOL_1_150_cov_38.258538_N_19797
MTSRLRFSQVSNNTTNRYTKELCTFLLWAFVCSGVVAEDFVPFVIPAKPNPDSAIAATSAKPIDTDSD
RLIADGHFYRGGQRVRLWGVNLSFGANFPKHEDAPYVAARLAAAGVNAVRCHHMDTARWPRGIWNAED
GKTISAEALDRLDFFIDQLARRGIYVNVNLHVGRAHSEYLGLPKTNRQYDKVCNIFTPALIEAQKQYA
RELLTHINKYRKVRYADDPAVAIVEITNENSFFMWDGDKALRALQPYYADVLQGKFNAWLRRQYGADA
NNVALFPDGQATEQVLDRMRFLAETEKAHFDDMRKFIKNELGCKALVTGTIVFGPLGLYAQSDMDFID
THAYWQHPRFPGRPWDSNNWIVEQRAMTDYPQEATLFRLAAQRLAGKPFTVSEYNHPAPLDSQAECVP
MIASFAAAQDWDGVWLYAYSHGTDSWDREVLSGYFDVDTNPAKWGFMRAGAAIFRDVDIAPLGGFIIR
SWTGPAGILPTLAKLHLKYDGDMLRALGGQPETTIREMLKIQLVPVIGNRDGHRDVFGPAAKLEWSVE
DGKGLFCASGRGAQVYVGHAGRFKADTQGKIVVIGPSFVALTVTALDEKWLDKSQKILVTACGRCENT
GMKFSEDRRTVGTNWGSPPVQIEAVEGSLRLDGRWKCRALGPDGLPKHDVVVSTEAGQSLLKLSPEYG
TMWYLLTRAANTDEQR*
>SOL_1_150_cov_40.634373_N_22005
MAERDFSRIKNSIQNIATFALALLWLAATPAQAALPELRVPDGFGVNIHFTGQPRDLDLIAEAGFKFI
RMDLSWSGIERQKGVYNFERTGYDALTEGCTKRGIRILYILDYSNNLYESDSSVRTEQGRKAFAAFAE
AAAKRYAGKGILWEIWNEPNLRQFWNPQPGADDYCKLVAETAPLVKRADPTGLIVAPATSGIPLGWLE

QCFKQGLLSHIDVLSVHPYRSKPPETVIADYTALRELIGRYAPAGKQIPVISGEWGYSNINWDKNPLS
NNLQAQYLARMFLTNLYQGIAVSIWYDWKNDGTDPNEREHNFGTVGHDLQPKAAYLAAQTLASTLAGY
TIKDRIDLGSGDDFAFRLVKDNAEAIAIWTAGQEHQLTIKAESDKGTLISIVGEKTAIDWTSNELKLA
VSPSPQYLLLGVKTPR*
>SOL_1_150_cov_12.727916_N_30146
MFNTRTRGRIIIGVLIVILGVQMVSSVHGSQVANSMDERGFLRTQGTWIADQLGNVLVLRGVQFDGYQ
SGHWDWHGMKDYERIASWGFNVVRLPVAWDFIEPQPGKYDNSYFSKYVDRDVAWAKKYGLYIVLTMFQ
YGWSSHFKYYDQHVSCGVPSWSVSGYPDTADGEARAKADFYNGLGPNGTPPSSTNPSMRDRFMAMWKY
VASRYAGKTTIAAYDLLNEPTVFSSDHKVSIYDPGSFYSETEVAFLTGALDAIRTVDGNHMIIWEPTM
EQRPPTSRVDRPNVVYSPHYPGSTGSASASHLSFYHYDGNKIWLEDFLEKKVIAVSQQWNQPVFIGEW
GICVEATNATQFIRDFLDLMDKYYLGGTCFGYGKAPWGMYLLDKSGNMRTALVENLVRPYVGVSSAPF
SSSFDTDEKKLIISAKGGTILGVYLPSSYSSYTLTNDIGEASWTVDGTILKVSVFTELSQVVLKFS*
>SOL_1_150_cov_26.611228_N_35450
MKQNRGVFVMMVLAALALPAAWAADPASAFVRVSPRDARYLELTDGTPYIPIGLNMISPPRVKAGEAE
ALGGMASWLESLSSNGGNYIRVWVSSPFWDIEHEKSGVYDQAQARRIDRLLELCEQHGIRVKLTLEHF
RSIGGGSQKWADKPLHHVSNGGPAQSIADFFDGEASRAQFRQKIRWYGERYGPRPIIYGWELWNEVNA
VRGGDYMTWTGLMLAELHKTFPQNGEKPEVLRGLSVDLRPALGGRNPRRVRVYDPWPGNWSNGKMRRG
ILQLPPFSRSIVIQAM*
>SOL_1_150_cov_16.567801_N_40972
MTRNFRWRPGVLLVVIGLVVAFLLGRPLGALPWPAASGPAGSEQPAVMSPPAAAAANLAADDTPTPTL
TPETPTITPTATPTPTHTVEASTPTPTATVTPTQVLAYLPVLMHDYPTTMPAYGMHLEVFLTDTQTIT
YAQAAGMHWMHTRIMWADVEPANTTPEHYNWADYDARLQAAGHAGFQTIAILLYNPSWAATLPGGNLY
EGMMSEFTEFVAATVSRYKDPPYNVKYWEIYNEPDNTWLKYAQLDWWGNWGHDAAGYVEILQAAYTTI
KAIDPQAQVLLGGLAYDWFTDDSTPGPFARSFLADVLALGGGDYFDILSFHYYPCFRWRWDSQGPGLR
GKANELRETMRAHGVVKPLVVTETGWPYDSMHWGITDPEHLLQPRYVAKPFIEALMMGDMPFVIWFPF
RDHDPGYTNGFMDYYGNVRQSYQALLTLTGQLAGMSYERLLLPEEGEADDVEGYLFAGVGGQPKLYTV
WTLTDTPRQVSVPAQAVQKTDHLGATTTLHDGDDGAVDGYVRLTIGPDPVFLKVIH*
>SOL_1_150_cov_7.487856_N_52097
MDLLRVKGNQIVDRQDDSVSLRGVCVGGWMNMEDFINGYPGPESGIRDALAQVLGAGKAEFFFDRWQD
YFFAEEDVKFIKSLGCTVVRLPLNYRHFESDLAPFQYLEKGFARLNQAVEWCGRHGLYVILDLHSVQG
WQNTDWHCDNASRHSLFWTQKQYQDRFVALWEEFSRRFMGNPVIAGYNVMNEPVANATRGLFDWQNYE
PGYAALNQIYQRVVRAIRAIDPDHIIFLEGDWFSKCFDGLDAPDTLGGNIVYSSHNYNEAGFGPGIYP
SAEKGWGKAWQEKIFSELSGTRFTRKHNVPLWVGEFGSAYNGPEREIPDRLCALDDQIAVFNENRAHW
TMWVYKDIHVMGWLQVSPESAYFRKVVPILKGKQAVFADFWMGWMPGTPVKNSVDALAFQIQETLADV
GVDVTVSPRYVEQSVLSGYTAVLMQPAFARLFAGMSETRLDEILKSFSFANCRPHQGLLDVVRRNILS
G*
>SOL_1_150_cov_18.219570_N_54267
MKLLTQMRMVAGMAIVLLLVGSLLASLEATAMNMGSHEGSCLVAVPTVPFGVQLQGEENNPALLAKAA
AMGAKWARVTLSWASVQSTDTTPPTYQWSGYDTVLSNIANAGLTPVVTIRENPSWAAPTVCGPFYAGV
MDKFADFLTTAVNRYKGPPYNVTYWELYNEPDNIESSPYPWVGGCWGDRGARYAAMLQSARSAIKAAD
PTAQVILGGLAYDWFTGSPDWGVFYRYFLDDVLASGGGTQLDLFNFHYYYYWGLKRWSVYGTDILGKF
NYLKSQYGTSKPWMCSELGVPSTGGNSDPDITYTEEMQANYVVQGYVRAMSAGISPIIWYSFAEYVDG
GGRSFGLLRSDGSEKPGYRSYQVLTAQLAQATVQGALALGSGLEGYRYTVQGQERSVLWATDGDSHAV
AFTGSQVRTVTRDGTESLITDGGSGDLDGQVNGLVTIAVGPDPLYVHSVTAPRFVCYLPLILRSTEGV
AGYALPASSGNGAICGGIIPWSLLRELKQKLLPWP*
>SOL_1_150_cov_6.555125_N_60555
MESQDTNFLHVHGEKIVNGRGETVLLRGFCLGGWMNMENFITGYPGHESGLRAAVAQVLGDEKARFFF
ERFLHYFIQEDDLQFIKNLGCNVIRIALNYRHFESDDQPFGYKAEGFALLDKVIGWARDLQLYVILDL
HAVQGWQNRGWHCDNSGGEPRFFGQKVFEDRAVALWQEFARRYRDEAFVAGYNVMNEPDADEVARLNH
YYRRVAAAIREIDFHHILFFEGNKYSQQFESFDPLFDGNAVYSSHNYVEPMDSVYPGTVNGEAFDRAR
LERDYRSRASFMLRHKVPNWVGEFGCIYENPARSESNLRVMNDMIDIIEGHGHHWSIWTYKDIGMMGA
VLVNPESEWMRRTLPVRQSKTRLRCDSWIERQTQPIDNLVGQIAEYAAEMIPSASESEILFKLYYGIQ
DGILAQVLLPAFAEQFRDMSETEIDDMMQSFAFKNCIPRTGLVRLLRMKLQG*
>SOL_1_150_cov_7.443673_N_61079

MSETSGFNPEAKYAVTMWDFSWLVRRTGNEAEYADWDKVLDELAERGYNCIRLEAFPHLVAKGPDGKL
VERFTVLPQEDYFMWGNHEPVQVEPRPALVEFISKAAERGIYVGLSSWYNRDTLGRVYMIQSPQDYAR
IWLETLDLLNEAGLHDRIVWVDVCNEFPVNRWAPRAYADIFQSKRAGDLWMLLNLSKRWDDGVKQRMK
SYFDGAITPLREKYPDLKYTFSFQALGSRQMQEIDTSAFDVAEVHIWVSDYMKWLFGTGQVLAQFGFP
KYPKNIKIHARRMAKLYPKHREEYVKMLEARIDFWAEWGKKNSLPLFTTEAWGPINYDDVTPGGIGGE
WDWVKDISEQGVRMASERGWQGISTNNFCQPHFEGMWADVGWHKRITDLILRG*
>SOL_1_150_cov_5.678139_N_66434
MAHPGRRERTVVLVATALAFAAFLVGLGVSLPLSASPPDMASSQSLLGPEEEALITLINAYRQENGVS
PLSVSPTLTQAARWMSQDMAEKGYMNHADSLGRNFSQRLTDFGYDYNTYIGEDLARADDNYQTTFLLW
QSSPGHEANMLSPNYRVIGVSRTHDGISSYGWYWVADFGGYDDSDAPWPTPTPTPTPTPTPTPTPTPT
PTPTPTATPQAQGRALSWATGPTSSQMQDGILHNCPQPGSWAISAWNGADSTETGQALGTCGEGNVDF
AYYIDPDTQDWLHYFVGLREFNDLPALDNGQAVITHSAPDAPPPTQTGTDAGPVADKWSLWTEGTRLR
GANIYQRRVYPELDDSSMGTGPVGPPYTQEDFDRLAALGANYVNISHPGLFSETPPYTLDQGIQDNLD
SLLEMIAEADMFAVISFRTGPGRSEFTFFHDEVGTWFDDSYLNDSVWQDQAAQDAWVAMWRHTAERYR
NNPIVAGYDLIVEPNANDVWLDIWEPEEFYSTYANTLYDWNQLHPRITAAIREVDADTPILIGGMSYS
AVDWLPYLQPNADSRTVYTVHQYGPHEYTHQTPPLERTYPGLFDIDWDGVDDEFNRAWLENLLSAVDT
FASTHSAPMAANEFGVMRWEPGADDFMDDQMDLFEDLGMNHALWLWEAAWEPRAAMDDGFNFLHGPDP
DQHTDMPSSDLIDVIVQHWGRNTVRPSNFSEEAAAQGTLHNCPQPGKWALSVWDGADGTDISEVLAGC
TGATVAAAYCIDPQTQEWQHYCSALPEITTLNTLDWMQAVITLGKE*
>SOL_1_150_cov_17.495853_N_69303
MGRRFRTYPLVTRHSSLVTGVVVLLCLLGVAVWAAYPWTGRQAADPAEIFGVEMLGSVGPEQGLAQAV
AAGVRWVRVILYWSEIEPVRQVPPVYNWRRYDTLFSAAAAAGLTPIVIITGNPAWAAATPCGPLHQER
MGDFRAFLQALVARYGSARSGVRYWELYNEPDNVDLADPGSLGGCWGEDGGAYAQMLRVAYRAIKEAD
PQAQVLSGGLAHDLFTYDGGPFREAFLDEFLAEGGTSFDILSFHYYRPFHERWDPYGPDLLGKVAYLR
GKLADLGLEKTLMATEIGQPTACPPGEECSDDLAARYVVQGFVRGLAAGLRAVIWFTMVDYDDPRAYG
LLRQDLTPKPAYQAYRVLTQQLKGAKFSRTLSPGQGGWPDVEGYAFTSRDGGELYVLWAMGFVDQEIA
VPGKRLRLVTKEGTENSVSDGYAGDLGGVADGQIVVKVSADPIYLWLDTRDR*
>SOL_1_150_cov_16.341086_N_72899
MQRLAQERPWLTSLLLLASAVLVVLAGWLVVAIVQDDPMALLESGELRNRLLGIYTGITARPTQTADL
APIAHTDGPPVGVNTFLEQEVEEAKLRRTLALVRDAGIVWIRQEFPWDDIEIHGQGDFQDRRTQPPKS
AWDKYDRLVTLAGEYGLRIVARLDTVPAWARPPGSTFTHPPTNLDDYGDFVAAVVSRYRGKIRYYQIW
NEPNLAFEWGEQDVNAAAYARLLQVAYTRAKAADPDCVIIAGALAPTIDPGPRNRSDVLFLEEMYRDG
AAGYFDVLSTMAYGLRSGPDDRRIADQDVNFSRPILLREIMVRHGDAAKPIWLSEMGWNALPQGFPET
PRYGRVSEAQQARYTVRGLQRIQEEWPWVGVTFLWYLRQPGNWRDSQQEYYFRLVDPDFTLRPVYQAV
QAYATRPPLLLPGYHPASHWAIQAQGTWCSLPDAQADPALLSGRSFGAYRLGEVGAALAFTFQGSDLD
LVAWGDGRPGVLQVAIDGSAAGVSLPRDAAGRAVLDLASAGPGVGRFPLARGLPDGEHRVAITVADGT
VAISGFIVPRTPGFPAGLLWAGVGVALLWAVLWRLVGAWGGRET*
>SOL_1_150_cov_5.785638_N_74629
MIRQLHHNNRNAGPVQPACPEACPAGRTPDPRPQTQDFRPLSLVSWVLCLACSVAALVSAKARAVAGQ
DFVPFVIPAKPDANSPMAFTSYRPIETGSDRLVAQSGHFHRGGQRVRLWGVNLSFGANLPEHADAPQV
AARMAVAGVNAVRCHHMDSARWPRGLWNAQDGKTITPEALDRLDYFIDHLARHGIFVDINLHVGRVHS
QYLGLPETNRHYDKISNIFTPELIDAQKKYARDLLTHVNPYRNVRYADDPAVAIVEITNENSFFMWDA
EETLRTLPPYYAEILQSKFNTWLTGRYGSDDNLRTAWSQGAGPLGENLLSNGSFQIFPQGGGVPQNWH
LEQHSGCIASVSPHRRFRDALRVEISKADETQWHLQLNQGGLPVTAGQYYTVIFEAFSDMPRLIGCNV
GQAHSPWSNLGLSRQVELGQEWQTYRFGFVATDDDDNARVNFAFSGSTIPFYLANVQFAPGGRVALYE
GESIQAGTVALFVESETSARSLDRMCFLAETEKAYFDQMREFIRNDIGCRALVTGTVVFGPLGLYAQS
DMDFIDSHAYWQHPRFPGKPWDSNNWFVEQKPMTDYPAEATLFQLAARRLAGKPFTVSEYNHPAPLDS
QAECVPMIASFAAAQDWDGVWLYTYSHTSDSWDREYLNSYFDIDTNPSKWGFMRAGAAIFRCAGLDPL
NDSVVVSLTDTLDMAATLLSLAKLHLKHDTDMFAVSSELAGISRDDVLKARLFARFGSEVEPWPPAGE
PQKLFGPSARLDWSVENGKGIYIARGQCASVYIGHAGRFEEFPAAPGLISPAFGGGRQIVIVGPELVS
VTVTALDEKQVAQSQKILITACGRCENTGMGFSRDRRTVGTNWGRSPVRIQAVEGTLVLPEGQWICHA
LGTDGLVKQQIAVSYRDGQAELQLSPQYGTMWYLLRRDTGGE*
>SOL_1_150_cov_4.751269_N_76272
MKALMLILSLLLVSCTCLTKTKYLYDEEGRIKIYHGINVANSSKTSVDNLPWTKKEDLAKLNVWGFNL

VRYLVLWSALEPSKDSVNVDYINKTISRLNCLKKLNIDVIIDIHQDVYGPYFKGVGCGFPDWTVDDDS
IPFVLQQPWNANYLEPAVVAAYRNFWNNAEMQKQYVEMVSLVFSFAETLSNVVGIDVMNEPFPTMTDN
FVKALKEKKIQNMEPEDLIELATFELDHLSKLYTSILTEIKNKNYKKQLYFEPVIYTSAGIPTFLYFE
TENSVYFPHYYDPFCHEGKPYQYSNYELMKESMKIKVLEADKFKSPMLFGEWGIGQNVQYHQKYLVDF
LDFADQYTYGWTYYCYDPGHPFSIIDTAKVETPILGILCRPYPQRIAGINPKYKVGNDTFELTYINNG
CKAQTVIYVPFKANVTTAGSFTTEGNLIYYTNTKERKQHIYITPK*
>SOL_1_150_cov_7.020680_N_80649
MTLTPVSMTGNYFIKEERRFLPIGAHWVPAKTGLQWPLQWDPADLEADFVKMCDLGFNTVRFDLFWAW
FEPRPGDYNLEAFRQLDTFVRLAHQYGIYLHPTLFIGGEVGEAFWDLSWRQGRHPHADPEMLRLQTDH
AAEFARRYRSEPAILAWDLTDEPPFWIVGSQTTDAMAINWTRLIAGALRRYDPAHLLCVGTSMEDISH
GPFRPDNLSAEVDFFSAHPYSIYAADRFPDPMLSERGTYAGAFQVTLSSGAGRPTMIHEFGASSAQYS
PERIAAFDRVTLYSGLAAGACGFLPWCYTDAAPDLFKRVPYLRAPHETQFGLTTWDRQDRPRGEVLRE
FSHLLGQMDLTDLQPASGEAALIVPHEWAKPHGDFSRFGLSGPEAIPYTSTQDGSETGDANLWLMRSL
LNAFILARRAGWKVDMPREYSDWQSHPLVLLPSPLTSTERNLVHVHTAFWEQVRGYLQAGGAVYASLC
ADAAIPEMGDLFGATLADHAMEDEVILTLVQPFADLAVATTFHFHADSSSPRGWPATLDLCGGQVIAT
DQQGRPALITHACGKGKTLLCAYPIENYLAVRPAAFEGEQDTQRIYQAFGQWAGITPLFHTDQPSVEI
AALSGPERGYAVLANHSPEVRNVTVTSHLTIQSIRQILPNGSQTFPIKKRSWEMSLKGYDGAIIEWNL
*
>SOL_1_150_cov_17.135928_N_86583
MLNPRLLRRLLLTTLALALLTAACGPSPTPLPLPTRTPRPGAATITPTTGPVANPTPTQPVVTPGKPT
ATLAPGKPTPTATPVPGKPTPTPGSGSQQGRMQSPEYGVQVFLWWQPETTDRDLQLAKDAGFTWIKQN
FSWIDIEGAKKGALDWNEADRIVEKAQEYGLDIVARVDKEPEWARKGASKGPVADYNDYGDFLFQMAT
RYRGKIHAYEVWNEPNKAEEWGGKQADAVEYVKMLKVAYQRIKQADPKAIVVSAGLTPTTAGEPHVPD
DLFLKRMYVAGAKPYFDVLGAHGPGFKAPPETDPAEIASGKLKQYCPWGDLKLCARVWCFRHVEDLRK
IMVDNGDSAKQIMILEFGWDTDARQDSPYKWHAVTEQEQADYIVRAYQYAKEHWAPWIGVMSLIYIAK
PDWTENDEQYWWSITYPDGRVRPAYTRFKQWHQGQ*
>SOL_1_150_cov_5.010032_N_87313
MDSSRRDFLKRAVVGSAAMVVPAYAQQGQGQSGQAKAQTAIPRWRGFNLLEMFTMRSRGDFVEDDFKW
MRDWGFDFVRFPTVYRLWIEDGDDYKIKESMLAKLDRGIELANQSGLHVSLNFHRAPGYSVNAEFKEP
KNLWKDKEPLDAFCFHWQLMAKRYKGIGRDKLSFDLVNEPPSLGKVMSRADHERVVRTTVAAIRAVSP
DRLVVADGISYGNETAPELADLKIGQSTRAYQPMFISHYGASWVDSGNYPQPAWPGNGWDRRRLEEHY
KKWADLARMGVGVHCGEGGAFNKTPHGVVLAWLRDVLEILTGHGIGLALWNFRGSFGILDSGRTDVAY
EDFHGHKLDRKLLALLQEFA*
>SOL_1_150_cov_5.740289_N_96161
MNTGLPNNSIRVAAGLIIVCVLAVLLPAVQAQQSVTSTPATAYHVYFPLVGAIAPRNRLFLPLLASGA
VAEPGQSSGRAVASSASQATATSASGFVTRRGEELLLNGEPYTFVGTNVSYLTGPFFPESNVEGIIAY
LSASGVQVIRVWVLPWNDLDRVERMLDLGRKYNIRFILTLQDFYGNLDGSCFRRYETADLPHIHNVVP
RFAQRPEVLLWELMNEPTCPPSDSGLDCWDALYKWAEVTSREIKRLDPNHLVAVGTQNAGFDDQAGGV
FRRVHALDTIDLVSVHRPAGGLPEKESAIAHELGKPIYFGEVYRRGLGKNCQPLPDNELQRRAQAI
>SOL_1_150_cov_7.143639_N_101177
MENNRQDFHAKHKHREGPHFVENQIDNRLNRRSLLKIVAAGTAAAALPGYAQQPDKKQKMSEPAAQTV
LPRWRGFNLQNMFTMRSEGDWAEDDFQWIRDLGFNFVRLPLCYRLWITDGDDYKLHEPMLEKLDRAVE
LGDKYGLHVSLNFHRGPGYSVNREFTEPHNLWKDAEPLKAFCFHWQMLAKRYHGISKEKLSFDLVNEP
PSIGERMSRSDHERVVRTAVAAIREISPQRIVIADGMSWGNEPAPELADLGIAQSCRAYQPMFISHYM
ASWVRGEQFPEPAWPGHDWDRKRLEQHYQGWAELAKKGVGVHCGEGGAYNRTQHKIVLAWLRDVLEIL
TGHGIGLALWNFRGSFGILDSDRKDVEYEDFHGHKLDRKLLALLQKF*
>SOL_1_150_cov_7.027387_N_105157
MPSHHAFLKANQSHIQNQDGEDVTLHGVGLGGWMNMENYITGFPANENAFRQVVYRALGKEKADYLFE
RYLEYFFTDEDASFIRSMGLNLVRLPFNYRHFEDDMNPTVIRESGLKHLDRVIKICADHEIYTILDLH
AAPGYQNQDWHSDNPSRQAFFWQHKHFQDRAIWLWEILAERYKDNPWVAGYDPLNEPSDPSGVVLGSF
YRQVVTAIRKIDPEHIVFLEGNRYSQDFSVLGPPLPNAVYTLHNYAVPGFIDGGAYPGVSRGEYFDRT
VLQDKLILTCQYMLENQIPIWVGEFGPVYTGNPENDAMRYNLLEDQLSFYRELGASWCLWTYKDLGLQ
GLVSLRPDSKWIRKIQPVLEKKALLGVDSWGGVDKNVRHILEPIEQTFAEFFPNYKPFPFDAQWQINR
TVRHILLAEPLIEDFYPLLQGLECDEIDALMASFLYQNCSPRQELIQIVKKEDQIAQNS*

>SOL_1_150_cov_4.403069_N_110687
MDYSQIKGFNYQPSYGSSGFELWQKFDSTIIEKELGLGKKYFPGMNAIRLWLSWDSFIRNPRRFEADF
ETVLATTSRYDLLVMPVLFNRWHDAFLDYGGIYIDHFLPRVSWVQHERMFEPYLEVIVGKHASDPRIF
AWDLCNEPFSYSCAPEEIPDIVKAEYDWLKGLYNACKALGAAAPITVGIHSGHGLKGIQQIEPISDLL
SIHPYWTADSPPHSKADFVRLLDDYIQFASKTGKPLLATETCWGALDDMKRVDIIKYTLTELWKRQIG
WLVYLLHHSLIADAHRPEYGPIGPAGNLSFIEADGSLRPGHTIFKDF*
>SOL_1_150_cov_4.696903_N_111141
MMTDSAQTVLPRWRGFNLLEAFTTRSDGSFQEDDFRWIRDWGFDFVRIPACYTLWIVGEDVYKFYEPM
LEKIDRVIRLGQQYGIHVCLNFHRAPGFSVNRERQEPFNLWKDADALAAFCFHWEMFTRRYRGIPSAQ
LSFNLVNEPVRPSPDGMTRVDHERVVRAVAAIRRLDPERLIIADGVSWATGPCPELADLGIAQSCRA
YAPMGISHYKASWVGGEAWPEPSWPGALHDGLPWDRQKLEAFYQPWIELARQGVGVHCGEGGAFNYTP
HAAFLAWFRDVLEILTPQNIGYALWNFRGSFGVLDSDRQDVEYEDWYGHKLDRKLLTLLQEY*
>SOL_1_150_cov_4.567072_N_111827
MMMKRMWIAALVWGMVQSVAPAQPEPTARELPRWRGFNLLEMFYRGSSTGPFKEEDFKLISELGFNFV
RLPMDYRVWIKSGDWTQFNETAFQWVDQAIEYGRRYGVHVCLNFHRAPGYTVADPPEPTSLWTDVVTQ
KVCAMHWARFARRYKGIPNSRLSFNLLNEPSGVDAASYAKVVGLLVKAIHDEDPNRLVIADGLDYAVT
PCWDLIPMGVAQATRGYQPFTLTHYRASWATGSDQWAVPVWPEPLGFAGYLYGLGKKDMQSPMIIEAS
LPEPFTFRVRVGTVSASSRLEVSVDRPAGPIWSQDFKPGPGLGPWKQVVYMPQWGIYQNIYDQEFIIL
LSACRRQIRLNNTAGDWMTITEIGLQMADGRVFSLRVNPRWGESNRALRFDPSNPAGAFQSDVAIDRQ
WLWDKYVLPWVQLKEAGVGVMVGEWGAYNQTPHDVTLRWMEDCLKNWQQAGLGWALWNFRGSFGVLDS
GRADVPYEDFRGHKLDHHMLDLLQRY*
>SOL_1_150_cov_5.042314_N_113580
MYVPFQGATFVPVSFDAGTYTQGSISLPRLDAIAAKDASGKLWLEVMNLDPENPVEIEADLVGVAIKS
AAGETLTAPNVDSVNTVEGVKDETGLLMWDIMNEPFTSSYYERATGDEKKQREAEIMEFVRYNLTYVK
KLDPVNAITVGYTFSRELEPTADLVDVLTFHDYTMTRSSVEEVYRIAEEVSKKFGKPMLNTETGCIAR
GNPYDMVLEIAQEHKTGWYLFNLMIQGYWGEIHGLFYPDGTIRDPAIIAAVMGFYRNRDLNTMIKPVP
NREGHAERALKAIEAALKDNQSAFGASKTPTDKILDAAEAAANLLEAAEMVPMYVPPTAQIRFWRQQQ
PEKRDRDAIRAFAYDLGLTLKKYCLLY*
>SOL_1_150_cov_28.967232_N_119260
MLILKNHVRWAGLAFLLMILTMGCMRPAQAADAVDPFDQVKRLGRGVNILGYDPLWDNFEKGRFQERH
FKLIRDGGFQTVRINLHALQRLNAANGYRLEDAWLKTLDWAVKNALANNLMVILDLHNYTDIAKDPLG
YKPCFLAYWKQIAAHFQDAPDAVLFEILNEPNGKLTPQLWNEFLSEALAIIRATNPTRTVIVGPPFWN
SIDHLDDLVLPEEDRNLIVTVHYYKPMEFTHQGASWSKETAHLAGVTWGSAEEKKRVEDDFARVEKWS
LAHRRPILLGEFGAYEKGPMESRARYTAYVARTAEFLGWAWTYWQFDADFIVYDVDKDQWVEPIWKAL
IP*
>SOL_1_150_cov_5.302255_N_129552
MRSSVLALCAALFIMGAVASGMCMAHDQITLSTPQKQVGKYERVELLIEVGRQYRNPFDPCEVELNVL
VTGPSGRSLVLPAFYGQDYERQDVPQGGRTTAWYYPQGTGSWKARFAPIEVGAYVARASLRDRQGEVT
SAPLAFTCVASSRKGFVRTSTKDPRFLEFTEGEPFFVIGQNVAFVGETQHVTPVKAEQVFGKLAADGA
NFVRLWTCCQDWALAIEARKSVWTRSWTRESPVVPMPGAESDPNARKCVRLKGDNGASLTASPSHPVG
LRPSTRYVFAGKFRAEGCKAVRLQVGNNNWEVPASSSGSPDWQIFRQEFVTGGNERWLGRVALGLVGP
GTIWLDALSLKEAPGGAELLWEADVNRPIRGYYNPLDCFMLDQLIEAAERNGIYLMLCAITRDLYMNT
LSKVDSPEYRSATIDAKKFMRYAVARWGYSTSVAAWEYWNEMDPGKPTDRFYAEVGNYLAQTDIYNHL
RTTSTWTPSARDCRHPQLDIAQEHHYMRPDDDDFKDEVESIIRQTRFLRDNAPSKPALIGEFGLADAK
WGLSEYMKQDPEGIHFHNCLWASAFAGSSGTA
>SOL_1_150_cov_8.154934_N_143236
MSNSLKLVAILFVVSLLTACALPGVMETPALKEESAPPAGPALTTLPPAFNDKWSLWTSGTQLRGANI
YQRRVFPEFDGTEFIGPGPFGPPYTQADFNNLADLGANYVNISGPGLFTVRPPYVVDEEAVANLDRLL
EMATQADMFAVITFRTGPGRSEFAIIGGGDWLPYGYIIETVWQDVEARAAWAEMWHYAAERYRDNPIV
IGYDLMCEPNSNASLDIWDPETFYAHYAGTGYDWNAWYPDLVSAIREVDADTPILVSGNSYSGVEWLP
YLQPLDDPHVVYTVHQYSPHEYTHQEPPELTCTYPGYFDTDYDDSPETFDRAWLENLMSIAAGFQSTH
HAVLAANEYGLKRWEPGGADYIRDEMTLFEQYGWNYAVWQWHASWPPLAEGDNSFNILFGPDPANLTE
VSNALLDAYVAAWVRNTVRPSNFNP*
>SOL_1_150_cov_9.919986_N_145097

MMNAHKLSSKSIAALTLVLSAAIVFVSLGCSQTHHTDGTLKLNDLDYFETRGLNVLAFSNWYDVNFFS
DSKLSGIEIIHHEVRTATNGDVRLNPTPEQWDPIPEFVERKVNKQNNSIEASLTYPDYDFDYTIKTEA
HNQGILLSVILEKPLPQALAGRAGFNLEFLPAAYFEKAYLMDGKSGVFPLYPSGPMAVDTSGATEPKP
IATGKTLVLAPEDPARRITIKATNGELLLFDGRNKAQNGWFVVRTLVPEAKTGQVIEWLLTANTIPDW
TRPPVIAHSQVGYHPNQNKVAVIELDKNDTPLTSARLLKITETGKFIEIYKSRIKNRGKFLRYNYSTF
DFTPVKESGLYTIEYGNMRTKPFRIAPDIYENAWYPTLDVYFPVQMDHMFVNEAYRVWHGAAHLDDAL
QAPVNHKHFDLYAQGPTTDSPYQPGEHIPGLNIGGWFDAGDFDIRTQSHYYVVLNLVQTWETFGPKRD
ETTIDQKIRFVDIHHPDGIPDLLQQIEHGTLALIAQHRAIGHAISGIIEPTLSQYTHLGDGSTITDNL
IYNPNLQPHESDGFSSGTFDDRWAFTTKSTPLNYGSIAALAAASRALRGYNDPLADESLATAKKVWDE
EHTHEPDLFSFGNTTGGQLEAEELAAAVELLICTKDTQYAKRINELLPTIEKQFDRLAPIAVRAMPYM
DKSFSKKLEVLVRTYKDQLDKFYQENPFGVPVSTRGWAGSGLVIYSAITNYFLHKAFPEIIDPEYTFR
GLNYIYGCHPGSDISFVSAVGTHSKKIAYGNNRADFSFIAGGIVPGALIIKPDFPENKEDWPFIWAEN
EYTIGNAAAYIFLVNAVNDLLKETEL*
>SOL_1_150_cov_5.631499_N_156583
NAVPEHGSIPRALFVHDHQSIRLAKLSDDKGDSVTQTRLPDDSSTQSNRNGAILWAGMALLLIGAMGL
IWLLFLNKSAAPVAVVPTRTLRPTFTAVGPAVQPTSTPAPPAATLTSVPPTATPVVPSATPVPPTPIP
PTSVPLPTAPTATPLPPVPAARPLQMNSPEYGMQAFLWWRPEVASRDMGVIKDAGFGWVKQNVSWNLI
EGAGKGIYDWSRLDWIVSECNKLGLDLLVRVDCPPTWAGGTKDCLPPHTPPRNYADYGDFVYAVATRY
KGRIRAYEIWNEPNLASEWGGQPPNPKRYVDLLKVAYRRLKEADSNAMVVSAGLAPTTASGAIAMPDM
DFLRQMYAAGAKSYFDVLGAHGAGYKAPPEMSPDEVAYDSRYNHKEPGVGRIYCFRHVEDFRQIMVEN
GDSDKQVVVLEFGWTIDPRPDSDYYWHSLLDEQLQADYFVRAYQWAKAHWSPWIGLMSLIYIADPDWT
EANEQYWWAISLPGYPEFRPRPAYLKLKAMPK*
>SOL_1_150_cov_5.676929_N_157076
MLRLQKMMKQVLWLNCVLLSGVAVGDEKVVLRCDFDGAMAPGSSAGRLVVGFEGSQSLLIEQADRGTS
SRQFAVAAERLDDRFAMLRAVVKAENVSEPPKPWNGLKVMLVLELADGAKQYPQIQMPVGTFDWKQMT
QTIRLPKEIKKATLVVGLEEVSGRAWFDNLEVVVGRPERKGHRNETVFKGHDLPRLRGVMYGPRFKES
DIEDLAVKWNANLIRWQLNWVPMKEAEEWATDVDAYDRWLDGALAECDKALAACEKYRIKVLVDLHTP
PGGRTNGGVCRMFQEKRYQDKLIAVWDRIAKRYKGREVVWAYDLLNEAVEGTVADGLWNWRGLATKAA
EAIRAVDPGKPVVFEPSPWGGADGFDVLEPLDVDRVIYSFHMYQPHSFTHQGVYENKTAIEYPGRIAG
ELWNKERLREEMLPALEFQKAFNVQVYVGEFSVARWAPGDSGYCYLRDLIELFEEYGWDWSYHAFREW
DGWSVEHGSDRNDHSPTKMPTKRMELLLSWLAKNQRP*
>SOL_1_150_cov_5.525714_N_157324
MSTTPQPRVSVNRAACAALVVFAVLILGLVVVFVLASMIGLLPVPGRPEATPTAPPSSPTSPASVETP
PVNTPVAPAVQTPSPTPPGTGFDYGIQVHLFHLDTGYVAQLVNNLQFRWIKHQIEWKEFEPTKGQYLW
EPIDQIVNPAHSSGLNVLLSVVKAPDWARGGHTTEDGPPVDYSHYGDFMAALAQRYCGKVQAYEVWNE
QNLSREWNTGRPLSAAEYVDLLRVAHEHIKAACRTAIIVSGGPTPVGYTSPTAIDDFEYLRQMYEAGL
PNYCDAVGVHPSGFNNPPDWLYPPAYSEAGDSESFRGNRQFYFLNTIEGYHDIMMNFGDTEHKLWATE
FGWASIENLTERPSERYEYAADNTEMEQAEYLVTALQIARNKGYMGVMFVWNLNFAPAGGAADERAAF
GILREDWSQRAAYQTLAAARATGELP*
>SOL_1_150_cov_6.119821_N_164382
MRAPVAVMLAAVAVVCACGGARAAAGTEVPFHRGVNLTSWLQAGSVRQIQFTQFTKQDFVNIKSLGCD
VIRLPINLHYMTGGAPDYTVDPLFFYFLDQIVDWCEELELHLILDNHTFDVDANTDPDVDKVLVPVWS
QMAEHYKSRSTYLCYEILNEPHGIADARWAQIQQQVISAIRAVDRVHTIVVTGAGWGSYNNLKFLPTY
RDNNLIYSFHFYDPFMFTHQGASWTNPSMVPLAGVPFPYGAGPMPACPPELKGTWVESSLNAYNRDGT
VKRVKELIDVAVKFRDERDVPVFCGELGVYRLNSDNDQRVYWYEVVRSYLQGKKIAWTVWDYEGGFGL
FQKGTNEQFQHDLNIPLVEALGLTPPLQTPFILTPDRTGFDLYTDYMAETVRNASYTGHGILDLYCET
APEQGAYCIYCTGFDRYNAIGFDFRPDKDLTLLVQMGYLLDFWVRGDTPGARFDVRFLDTKTTAPQDH
PWRMTDTIDQRYGAWDGQWRHVQIPLKSFVDRGSWDGVWLTPRGLFDWCAVDRFEIVSEYHDFAGMQF
WFDDIRVTKPPGAR*
>SOL_1_150_cov_3.533831_N_173575
MRTMKKIIREKSGILMRSKVLLIAFISFSITSDYAYGQKLVINDQGYFETRGVNVLVYSNQYTGMFFD
EKTAGIEIIHHGVRTSTGGAIRLQNTPEQWDLVPSVVDRKVDAATNTINAELNYKDFNFNSKISVTAK
NNGVEISVYLDKPLPKELEGNAGFNLEFLPSSYFEKTYLFDGKPGSFPRYPSGNTKVEPISKKITQFA
GHTTFDDRGRGEFIVPYPMATGKTLILAPEDPACFVKIQGVDAELMLFDGRNLAQNGWFIVRSLLPAN

KTGRVLTWYLEPNAIQNWIRKPVVGFSQAGYVPGQEKVAVIEMDMNDTPLKTAQVFQVTPESKFVENF
KGEVKVWGKYLRYNYAKFDFSSVKEPGVYFIQYGDQKTNTFLIAANVYDDVWHPTLDVWFPVQMDHMQ
VNEAYRVWHGAPYLDDCLQAPLNHQHFDGYRMGATTDTKYKPLERIPNMAVGGWFDAGDFDIQTGSHN
SVVLRFVEAWENLKVDRDETYIDQKTRYVDIHRPDGKPDILQQIEHGTLNLVAQCENIGHPVQGIIVP
NLHQYHHLGDAMTETDNLPYNPNLKPYETDGKSSGTLDDRWAFTTRQPFLDYSTAATLAAASRALRGF
NDDLADRSLVCAKRLLEEANEASKNANTNSNNPMMMRRGSDMDVVLQLYITTKDKKYADRFQEQIWAS
LEGPSGGGVAAGQGFFAGRALGSALKALPYMDEAFKVKLRNHVIKYKESLAEMDKQNPYGVPISLGTW
GGSGTVVSWAITNYYAYKAF
>SOL_1_150_cov_4.204799_N_182873
MKSSRAIERWAVLAVLGGSLLMVPRPAVAQSMQFVRVSGDRSGFVRDDSSQFVAWGFNYDHDETGRLL
EDYWETEWTKVQEDFREMKDLGANVVRLHLQLGKFMKQPDEPNEVALARLARVVTLAEHTHLYLDLTG
LACYHKKDVPGWYDGLSERDRWAVQARFWEAIAGRCADSPAIFCYDLMNEPILPGQKKETEWLTGELG
GKFFVQRLALDLAGRTPQQVAKAWVDRLVAAVRKQDRRHSITVGVIPWALVFPKAKPLFYSKEVSENL
DFASVHFYPEKGKIDQALAALAVYDVGKPLVVEEMFPLKCSAEELGAFIDGSRKIAEGWIGFYWGKTP
EEYRRSNTIGDAMTLSWLELFQKKAAAIQGQKP*
>SOL_1_150_cov_3.715108_N_189528
MKNNRRDFLEMAAGSAMAAALPAYAQRAEKKQEKTAKPVPSAVEGSVAQTAIPRWRGFNLLDMFTMRS
KGEFAEDDFRWIRDLGFNFVRLPACYRVWIKDGDDYKINESMLEKLDRAVELGGKYELHVNINFHRGP
GYSVNREFTEPFNLWKDKEALDAFCFHWQMLAKRYRGISKEKLSFNLINEPPSIGERMSRADHERVVR
AAVAAIRQASPDRIIIADGLSWAREPMPELADLRIAQSCRAYEPMFISHYKASWVDSKNYPEPAWPGN
GWDRKRLEEHYQKWADLAKKGVGVHCGEGGTFNKTPHKVVLAWLRDVLEILTGHGIGLALWNFRGSFG
IIDSDRADVQYEDFHGHKLDRKLLQLLREFP*
>SOL_1_150_cov_5.205312_N_192412
MTREGKLSSHVYAMVVFVLCLASASLAVDPFVQNQKLGRGVNIIGYDPLWKSRDEARFKVGYLGMLKE
AGFSTVRINLHPFRRMDQSNDYALPASWWETLDWAVTNALQSGLMVILDLHEFGAMGTDPETNKLKFL
AFWRQVSERFRDAPDNVLFEILNEPSRKLTAEMWNQYYREALAIIRRTNPVRTVVIGPAGYNSIGQLG
EFKLPEEDRNIIVTVHYYSPMDFTHQGASWAGRANKLGVEWKGTDQEKAAVEKDFARAQSWAKEHNRP
IFLGEFGVYDKAPMESRVRYLAFLVGTMEKLGWSWAYWQFDSDFVLYDVKSDKWIEPVRDALVGRAKP
RNK*
>SOL_1_150_cov_5.312567_N_197992
PWFKDEHGRTLILRGVNLGGSSKVPYCPDGATSIRQGFYDHRNVSFVGRPFPLEEADEHLARLRSWGL
TFLRLLVPWEAVEHGGPGVYDLEYLDYLREVVRRAGEHGLHLFIDPHQDVWSRMSGGDGAPGWTCEAV
GMDVTRFSETGAAIVHAIHGDPFPRMIWPSNYAKLAAATLFTLFFGGNDFAPRTLVDGEPVQEYLQRH
YINSMVQVAQRLRGLPAVIGYDTLNEPSPGYIGWADLNEAGPLLLGESPTAFQSMLLGAGFPQDIGVW
EQRLSGPKLLERRPINPQGVRIWQDGRDCIWRGHGVWDVGAGGQPRLLRPDYFAQVHGRPVDFNDYLR
PFLKRYAREIRSVEPDAIIFLEGVPSAGHPRWGVEDAPNVVNGAHWYDGLTLFTKNFSPWVTLDFFSR
KIVAGPWRVRPCFVGQIARLKAEAEEKMGGIPTLIGEFGIPFDMQGKKAYRTGDFSLQARAMDASFQA
MEANLVSCTLWNYTADNNNLRGDQWNDEDLSIFSRDQQGEASRRDPMAYLDEGGRALPAVVRPYARAT
AGEPLRMRFDVRTRGFEFEFHHDAAVSAPTEIFLPTYQYPHGCRVEVSDGTYQVDPATRTLVYRHSTE
RQVHRIQVKPH*
>SOL_1_150_cov_3.962919_N_213941
MRRSLHTVVLLAIMLFTLAEPLRAQFKDFVTRSGDKLMDGDRELRFISFNIPNLHYLEDYLPFSGINP
WRLPDEFEIRDALTSIKQLGGKVTRMYVISVRRQDDAPGIIRHVEGEGKFNEDAFRALDKVLQIANEV
GVRVIIPFVDNWKWWGGPADYAAFRGKEREAFWTDPVLIADIKKTIEFVINRNNTYTGILYKDDKAIL
AWETGNELVAPFSWTKEIAAYVKRLDTNHLLIEGTLAKELSQEAINDPNIDILSTHHYGDPKVSLQHI
VSNQMLAKGKKPYVIGEYGIVPTQDIRAITDTIIHQGIAGGMLWSLRYRNKEGGFYNHYEYNKVSAYR
WPGFSNGEFYDERMVLSIVREKAYEIDGDTQPRLPVPEKPTLLTINDVSSISWQGSVGAQSYIVERKE
EDSTEWRIIAEDVDESRYQYRPLFCDESATFGKKYSYRVRAKNESGASDYSNVGGPIEVATKKLVDEM
ESFDKVYQKDGDLKLLTFQDIRKAKEDRSRLTGGKNSYVIYKVPATVSAIKVDAYRVSEGGEVRVAAD
TSPNAFADLPTRTDVFRFGKNDYGFYDAVSCVCDEIPPGTKFIKIFLNEGIQIGRVEISYRSSF*
>SOL_1_150_cov_4.999362_N_225508
MNYPGRLVNPEEIAKLDPEVARIVNGNNGVYNRERLESLLAKPLELAKKLDLPLYCGEWGALPTTPRE
MRMQWYRDMRANLDKHGIAWANWDYKGGFGIVGRGGQPDQEFIKILLGR*
>SOL_1_150_cov_4.999362_N_225509

MLRYVITAAVLSMSLSAEAAQKDDGFTIHRGTNISHWLSQSTRRGEERLRWFTEKDVELIASLGFDHI
RLPVDEEQLWDEAGQKEPEAFTLLHNAIGWCQKKNLRVIIDLHILRSHHFNAKEKPLWTDPKAQEKFF
ALWRDLSAELKKYPVSLVAYELMNEPVADDAEQWNKLVETLMRQIRQDEPHRKIVIGSNKWQSVDTFD
QLRIPPGDRDIILSFHFLHPDVDHALQGFLDRRGQVQGAGELSGPAREPGRNRQA*
>SOL_1_150_cov_4.640285_N_228401
MKTHKPYFKNKSVIKFFLSATIAFSLLGCNQVHYTSYGLKLNDLGYFETRGLNVLVFSNWYNENFSDS
KMSGIEIIHHEVRTATNGDVRLNPTPEQWDPIPKFIECKVNEDNNSIEAFLAYPDYDFNFMIKAKANK
GGILLSVNLPKPLPQALEGRAGFNLEFLPSAYFKKTYLMDDKGGIFPLYPGGPMALDKMGSVEPKPIA
TGKILVLAPEDPSRRVTIQAKDCELLLFDGRNKAQNGWFVVRSLIPAGKSGKVIEWFLSANTIPNWTR
PPMIAHSQVGYHPNQKKVAVIELDKNDNPLTSARLLKVTKDGKFVEKYKDRLKKWGKYLRYNYSTFDF
TSVKESGLYVIEYGDIHTEPFRIAADVYEDAWHPTLDVFFPVQMDHVFVNEAYRVWHGAAHLDDALQA
PVNHKHFDLYAQGPTTDTPYKPGEHIPGLNVGGWFDAGDFDLRTQTHYSTLLSMVQAWETFHIERDET
AIDQKTCFVDIHHPDGIPDLLQQIEHGTLALIAQHRVVGHAICGLIVPELSQYTHLGDASTITDNLIN
NPKLGNHESDGLTSGTFDDRWAFTSKSTPLNYGSIAALAAASRALRGYNDGLADECLATAKRVWDQEH
SHEPNLFYQGNTTGGQLQQEELKAAVELLICTKEAQYATKINELWPTIERGFGFNAALAVRAMPYMDE
SYSKKIEALVRAYKEQLDKFYEQNPFGVPIPTGGWAGSGWVVNFAMTNYILHKAFPEIIDPEYTYRGL
NYIYGCHPGSNISFVSGVGVDSKTIAYGNNRADYSFIAGGVVPGVLILKPDFPENKEDWPFLWGENEY
VISVCASYIFVVNAVNDLLNGVE*
>SOL_1_150_cov_3.578492_N_239004
MVVAALSLGWPVLGTGRADGRLPMLSARGTSWVDASGKIVALRGCNLGNWLLQEMWMHSIRTEGIPDQ
YTLEQVLSQRFGQGAKDRLMETYRRNYITPRDFRIIKSFGMNVVRLPILYTLLEDDDHPFQLKPDAWV
HIDRSISMAESEGIYTILDLHGAPGGQNPWHHCGRADQDKLWGNDENKRRTVWLWQQLAKRYRGRNAV
AGYDLLNEPYTAPKDELRELALALYRAIREVDPDHIVIFPALGDGFGFYGDPRELGLKNIAFTPHFYP
GFFGWGEPTVETHTKWLTEGVQEWQRQAQAAGVPLLVGEFNPVLRAAGGAEMTARAFDTYTSLGWAAT
MWSYKVFSSEGGIAGGSWGMVTNPPAGPAPGSQVDLNTSTLEQIESYFASLSTMEYVVYEELGDRLTG
VVPQAAAVEAP*
>SOL_1_150_cov_3.304638_N_241641
MKAAFVVCVSVLFSLTCFASESVMTKEQVNEWYAKTGWVIGCNFIPSTAINQLEMWQKETYDPNTIDR
ELGWAEQIGFNTVRVYLHYLVWQEDPNGFKSRMDDFLTITARHKIRVMFVLFDDCWNGNARLGKQPAP
KPGVHNSGWVQCPRYAEVNNVSMYPVFEKYVDDLLRHFGKDDRVLMWDLYNEPGASHRPEVIFPFLKK
VVEWARTANPTQPMTIGVWKWDKNFSGLNEFQL
>SOL_1_150_cov_3.021529_N_251474
MNYRFVTVLFLILLTPVYTVGQGFLKVKGIEIVNEKGENVLLRGIGLGGWMLQEPYMLQLSKVAGTQT
EIRTKTSGLIGEENTKKFYKYYRRNMITRRDIDSLKV
>SOL_1_150_cov_3.743218_N_251647
LLPCLSSSLLLCYATVIRRQQSHYRVSSTAFCWGRQHTLPHPPEDSVCRCWKHQLGDEACRRPVHHSE
GVDTLTIAVVALVLLVLRVLPASAAELPGPVVPDGLGVNIHFTGEPARDLDMIQTAGFRFVRMDFIWE
LVEQEKGRYDFRAYDELTEGLKKRGIRILYILDYEHRFYESDRSVRTEEGRAAFARFAAAAASRYRGR
GILWELWNEPNIEVFWKPEPNANDYMALAKAVFPAIREADPAALRVAPATAGVDFGFLEACFKQGLLD
LVDGVTVHPYRQDLPETAGADYGKLRALIRKYRPNRPDVPILSGEWGYSAAWEGYDERRQGDYLPREF
LTNLSLGIALSIWYDWHDDGPDPEEPEHHFGTVALDYREKPAYQEMRRLVTALKGKRFVRRLPSDPDD
YLLVFSEGAGESTLAAWTIGSPHEVSAAPGLTVPLTGTPAYLPMPASGPAVER*
>SOL_1_150_cov_3.330588_N_253127
MKSLKIITAAFLLISGLSTENAFADQPLKDYSFIRGVCYPGGWSGDQKIIERDMGYAKKLQLNSTRIW
LSYTRYNQDPEGYTEKLRNYIRTACSFGITTMPILFNGNSLNPETLTKEFKEKTGDKYVKAIIDAVKD
EGGLLMWDIMNEPSYCDYLLKSPPEEKEKRKDEVKNFVRYYCSYVKKLDPRNAITTGHTFAADIQMDA
DLVDVISFHDYLATRKNIENSYTEAEKYAKQYNKPLINSELGCIGRANSYDMALEICNNHKVGWYVFE
LMIQGYWRDIHGLVYPDGTIRDPAIIAAIFGFYRNRDLKTGIKPNPNKEGHADKALAMLEKALKDETT
VFGNKRTSSDDLLEAAEYCANLLEAAEMVPMYEPPTAKIQTWRAMPEQERDLEAISEFAYELGLTLKR
YCRIL*
>SOL_1_150_cov_3.369780_N_255408
MNELGRRGLATPPYVQVGMGLQAHPIPEHRAKVSPRFFLALVGLLCAVLLSSSREGVMAEGKAVQFRG
VNGALQLTEEDISHLAQDWRANSLRILLSHGRAIQTKPPYEYVEEDLKQLDAIVGWCEKFGVNCIINI
HEAPGYVYVSGQDNSLWTSPEHQERLVRAWEMLARRYRNRSPLVWYDLLNEPHGTDEKPGAVGSWNRL

AKELTAAVRKIDPDHTIIVEGTGWAFPRGFGALEPTGDANTVYDFHWYLPKGYSHAKPEDKVTYPGAL
ANWTGGPPSQWDKARMLKELEPVFAFEKKFGVPVRCGEFGVVRWAGGAAQYLKDFVDICEERGYDWNY
YSYREWQAMNLEYGPDPNDRTRQETDRLGVLRRYFALDRFAPTHE*
>SOL_1_150_cov_3.874046_N_256341
MIKLIGHHFKDESGRTLILRGVNLGGRSKIPVQPNGATWNREGFYEHHAVSFVGRPFPLNDADEHFQR
LRAWGFTFLRFLITWEAVEHVGPGIYDQEYLNYLYQVVKKAGEFGLDVFIDPHQDVWSRFTGGDGAPG
WTLESAGMDLSKLHTAGAALLHQEHGANYPPMIWPTNINRLAAATMFTLFFGGSDFAPRTKVGGLSIQ
EYLQSHYINAVKQVALKLNDLPNVIGFETLNEPFAGYIGATNVADRAISPLLKGESPTIFQAMLLGAG
FPQQVDIYDLGLTRFVKKGARVVNAGKQSIWREGYEDIWKQNGVWGLDSSGLPQILVPDYFCKVGSRK
VDFYRDYFKPFANLFAQEIRSISPNAIIFVEGVPQQGELNWNMHDAPDIVHVAHWYDGLTLMNKSFNS
WFTVDPRNLKIHLGNQRVRRCFADQVAGIHRQSDEQMSHAPTLIGEVGVPFDLQKKRAYRTGDFSMQI
KALDATMSALEKNLACFTLWNYTADNTNEHGDQWNDEDFSIFSRDQQLRTGDVYDGGRGIRAVVRPYA
ASIPGEPLSMSFDIKTRCFHFTFRCDPEIRAPLVVFIPAYHYPKGTKVQVTKGRIEMDLEKQRLEYFP
DVSEFIHTMTISPS*
>SOL_1_150_cov_3.244538_N_257127
MFNRSEMKKQSARNLRRAGELLLLLALIVIVPSSLPNVSAQVNNLPWLHVEGGRIVDESGSVVVLRGA
AVEDPYYLKYIEKRFDGRDFMELSQNWHVNVIKVPVCPDLWEHNPNYTQELLDPVVDWGRQYGTYILL
GWHADGNPITGEVPIPDWGSTPPWSGNPYNPNMTLATRFWNQIAERYKDMPWVIYEIFNEPTYISWQQ
WRPVAEQLVDVIQSHNPRATIFVPGVGWGYDLRGVGSDPVQRQNIVYETHPYPGQGAYVGPWETFFGY
LAGSYPLFAGEWGFVPGSSNWNLNATAENYGTALLEYMANLGMSWAGWSWSASWSPRMLQNWNYQPTT
FGQLIKDVLAAGLPTSAGKIVSVSVSPGSVSGKMTVTFAVTVENTGNIAWSSATVLIKIYRPDGSLAD
TASHRQIHLENSQIQPGNQYTYETSWKTPSKGSKGVWHYEVFVNSGSVLIASSTDIANTITVN*
>SOL_1_150_cov_4.741125_N_264603
MKRWCDGALRWVLAGMGVAVLLVGATALAGATETNPALGLMAVRPDGQGFVEKPSGRPFTPFGVNYYD
PNTGWAPKIWRQFHTNRVTRHFEVMRDLGVNCARVFLTAATFQPDVNTVDEQALTKLDTLVRIARQSG
IRLILTGPDHWEGSPAYWKPDRFAGERALKALDNFWRAVGQRYRGEPAIFAWDLLNEPHLPWFAETWR
PLWNKWLQAKYGDWGRLQAAWAGELKEGESWDNVTVPENKASKGNPRLLDWQMFRESLADEWVRRQVE
AIRQADPTHLVTVGYIQWSYPLVRGGNPSQYAAFNPQRQSRWLDFMCMHFYPLLGRPLESKEAWDRNL
AYLQSVLAYCHVGKPVVLEEFGWYGGGAPRGQPSLTEDEQDRWIVAEIEATRRLSDGWLSWPFADTPT
STDMSMYGGLVRSDLTRKQWAKSFAKYASNLAALPQPTPQLPTFDRRCPGCR*
>SOL_1_150_cov_4.214114_N_265466
LSYDNGDTRANIGHNIGWNTGGIYGWNNYLKKMHTAGENWVRHWMCRYGSDWGTILEWKSGGYFQGAG
KLSMQIALRLDRYIEIAEQNDVAIQLALQHHGQFSTTTNPDWTDNPYNTAAGGFLNTPDEFFTNAEAR
RLTKNKYRYIVARWGYSPAIFAWELFNEVQFTDGWGSNQASVVNWHNEMSAYIRSIDPFKHPVTTSSH
GSGFENIWNLPDINLIEVHYYGTDTINTFEQTARGLAGFNKPVIMGEFGTGTGGQTDPEGLELHNGIW
SSFLVKSSAHLWWWDNFIDPCNLYGVFTPLAIYAANENLANYNLSRAQRAASGNEAYYASPVLSDFWA
VTTQFDFYLQEDYFPGMENLSRWLHGSDKSAWKSDPNFHLNMLTAGALKIHVQSTADWCDNSLKVLVN
GGQVFSHTYPKDSNNFTITVPLSAGQQSVHIVNTGADWFNISSYEFAPNDISFLDSIGLSSNERAYIW
IYDVNSQIGLTNHGVINNEPVIVKGLDDGSYTVEVYATRGAGGVIDSGGADSVSGVLTYTLPDFEKDI
AVKVKPLCILVGFDDLAAFCEQWLQEGPGLGADLDDSNNVGLKDYSIFAGYWLDCRPADWPSM*
>SOL_1_150_cov_3.825279_N_271329
MKKVIRLVTIIFAVLFCLQNTNAQQTTQEKEYHKWWNEGYGRRPQDNPLAKKLPLIQVKGNKFIDSQG
KTILFRGLSISDPDKIENQGHWNKKHFEKVKEMGALIVRIPGHPSGWRERSPSKYLELLDQAGEWCTE
LEMYILIDWH
>SOL_1_150_cov_6.820652_N_273735
TLLLRGVNLGGSSKVPYTPNGATYIREGFFDHRQVSFVGRPFPLEEADEHFARLRHWGLTFLRFLVTW
EAVEHAGPGVYDQEYLDYLHAVVAKAAQHGIRLFIDPHQDMWSRFSGGDGAPGWTFAAVGMDLTKFQP
TGAAFLHPVHGDPLPRMIWPTNATKLAAASLFTLFFAGKDFAPQTKIEGEPAQEFLQRHYIEAIRQIA
MRLRDLPNVVGYDTLNEPLRGYVGWRDLTLPHAVVELGAIPSPFQSMLLGAGIPQEVDVWQRRLTGPK
RIERRLMNPDGQRAWLDGCDCIWRTNGVWDLDPAGRPRLLRPDHFTQVGGRTVDFNRDYYVPFNNRFA
RAIREVHPGAMIFVEKEFELAAPDWGPGSAGNVVYAPHWYDGFVLFTKTFSRFLGSDNLNHKLLVGPG
RIRRAYKQELAAFKEHAAQNMGDVPVLIGEMGIAFDLNAKRAYRTGDFRTQAAAMDRTLRAMDDNLLS
YTLWNYTADNTNARGDLWNDEDLSIFSRDQQAEPENVHSGGRALKAIVRPYTLATAGQPLRMSFDIRS
RVFEFAFRHDPQVTESSLIFVPDYQYPKGYRVEVTDGSYEARSDEQILSYRHSPEQELHTIRVKPR*

>SOL_1_150_cov_3.485776_N_279726
MRLVFKITILVMAGAVSVIAAEGAISETQAKSSSMPLPLKVKGTQILNSKNKPVTLRGVNAACLEWTS
DGEGHILETVRVAIEDWNVNIIRLPLSQDRWFGKEPNQSDEGRAYRALVKEIVDLCGSKGCYILLDLH
WSDVNEWGENIGQHSMPDRNSVIFWKDFAPVYANHPAVLFDLYNEPHNVTWDVWLNGGMITDRPNRPG
AQAKKYEAVGMQEMLDTVRGTGAKNVVVAGGLEWAYDFNGILDGRELKDPTGNGVIYANHAYDNKRES
VFTWIAKMEEASAILPVIVSEFGGSGGPNRSRVWWGQCPSTAM
>SOL_1_150_cov_4.149847_N_282432
MKVQVESSPDGINPRTFDTPHEKRSRPPMPQTLVSLKGNYLATQGRRFVPIGAHWVPARAALQWPVRW
DEKDIEADFIKMRDLGFNTVRFDLFWAWFEPRPGDYNPQAFHQLDFLIRLAHEHGIYLHPTLFIGGEV
GEAYWDVPWRHGRHPHADPAMLRLQTDHAAEFARRYAGETAILAWDLTDEPPYWIVSTRTTDDMAINW
TRLISGALRRHDPQHLLCVGTSMEDLEHGPFRPDNLASEVDFFSAHPYSIYSLGLFPDPMLSERGTYC
GAFQVALSSGAGRPTMIHELGASAAQYSPERIAAFDRVTMYSSLAAGANGFLLWCFTDAAPETFRRVP
YLRAPHETQFGLTTWDRQDRPAGQELRTFSRLLAQLDLNDVEPASGEVALIVPHEWSKPHGDFSRFGL
EGAEAIPYVSVQDRGGMPEPPGTSHERSEANSWLIGSLLSAYILARRAGLKVDLCREYTDWQDHPLLL
LPSPLTSTEKNLVHVHTGFWERVRKYVEEGGTVYASVCADAAIPEMGDVFGATLSDHTPVEQVTIKIV
APFGDLDPGEVFAFRPSADRAQHWPAILETAGEVLAVDQAGRPALVAHNYGSGRSLLSAYPIESYLAV
RPAAFEDEENTHRLYRALWSWAGLRPLFTSDHPSVEVAGLVGKKRGYAVLANHRPEAANVTITAREEL
KAVAQVTPSGLTPLELRGLAWSMEIAGYGGAIVEWRA*
>SOL_1_150_cov_2.743762_N_302494
NPANHYLGFPGRVSLMNRRMALGIAAFALCLSGVATGAATGEDAQGSLLTNGDFESDSKGANWPDDWP
HPASAQWEKEDGGHFLRLHPDAPGQMLVLYRAVPIPPECKALELTCRVRHTDVKPGPEAWHDARIIIH
FKDAAGRERVPAPAPLVFRGSSKGWTQCRARFLVPQKATLLEFMPALFKVAGGTLDLDDIVLKPVAPD
AVNQRAASAATRVEFQGTPVEPAKLPKELRVVGKQLQTPEGKEVWLQGLNVESLEWTAVGEHVLKSAA
VAMDQWKANAIRLPVKDDFWFGVGPWQKDGGKGYRRXXXXRLIDDVVQAAASRGAYVVIDLHRFGAPT
DDSLDFWKEAAARYKNHPAVLFDLLNEPHDISWEVWRNGGTVEDKKRNTAYESPGMQRLVDAIRQAGA
RNVAVAAGLAWAYDLSGVVKGYALEEKGGNGIMYSTHIYNWHRGWKENVLGAAEKYPILVGEVGCDAN
PMSFIPRDQQEDPATWAPDMLGFIQKHRLNWTGWSFHPKSSPRIILDWDYTPTPFWGEFVKAALGGRK
FEMGKMR*
>SOL_1_150_cov_2.601884_N_310141
LSFDLINEPPSMEEETRYVEVMKALVKGIREEDPDRLIVVDGKDVGRTPVFGVADLGIVQSTRGYDPM
SVSHYTATWVPKDSFQTFKVPTWPLIGDDGKLWDKAALKVLLIDKWQPLLDMGVXXXXKTPHDVTLRW
MRDLLSLWKEAGWGQALWNLKGDFGILNSQRTDVKYENYKGHKLDRKMLELLKEF*
>SOL_1_150_cov_3.085248_N_316911
MKRAVSVALVAALAAGAAAWAEDAAPSLLVNGNVEASTKDAGWPDGWPRAPGVTWELEDGNHFLRLRS
EKPGQTVLVYLAVPIQPEHKALELSYRVRYAEIKPGKESWFDGRIMMNFRDDAKQMAKPGPAHPNFRG
TSKGWQERRQKFLVPQGAKVLEIMPTLFQAEGGTLDFDDVRLVSISASEVPPPPPPPPIIPSETLTPA
DPKSLPPELHVAGNRLKTADGKAVWLQGLAVPSMEWSAAGENILQSIKVGVEQWKANVIRLPVKDDFW
FGRGKGQKDGGMAYRKLADAAADAAGSRGAYLALDLHRFGAPLPEHVDFWKDAATRYKNHPAVLFDLF
NEAHDISWEVWLRGGAIDDKRKAGGDAGAPGRRSPGMQALLDAVRATGARNIVIAGGLDWGYDLSGVV
QGFALEERAGGNGIMYSSHIYPWKKDWQVKALAAAERYPLFIGEVGCPPERLPFIP
>SOL_1_150_cov_2.789005_N_323010
MRTKRRFTPASCCHKTAAVVILSVILCLATQLYAYIPPLHVDGNRIKDPNGNVVVLRGVDLIDLGFLQ
DWQGGAINMINRLTNKTDSQGSSPGWYPKIIRINITPPDSVSGWPHPFNPGNTDLYDLLRLVVNYCAT
KDMYCIIDWHYVANTYEHVASTSQFWTYMAPRFADDRHVFFELFNEPINNTFGSDAANWASVKADMQT
WINIVRTYAPNNIILVAGPNWSQAIGPIASNPPTGSNIAVVSHIYPGHWRNPSWYQNHITTCAAVFPI
IMTEWGFSQSGSPDPGDLLNGTITGYGQPLSNFREQYKIGHSAWVASYDWGPPMFWSDWTLRIGEGEM
GGFTKDLLYTYRD
>SOL_1_150_cov_2.728000_N_327740
MQRLKPVKIEGTYFVRGNERFIPVGAHWVPSTGLHWPNEWNPDAIEADFSKMQKMSFNIVRFDLFWAW
FEPYPGVYNPEAFNQFDYFVEMANKYSIYLHPTFFIGGETGYYDVPWRNGRHPHGDPDMLRLQTDHVS
KFAKKYATEPSIAAWDLTDEPPYWISRGKETTDAMGVNWTRLISGALRRYDKNHLICVGTDQEDLRHG
PFRPDLIKDEVDFLSVHPYPIYLPALFPDQMVSERMTYCGAFQVCFSGGAGLPVMVHELGSSSAQYSN
ERIARYDMTSIFSSFAAGANGFILWDFTDAAPDSWKRVPYKLAPHETQFGLCTYDHINKPAGEEFLKF
TKVVAEMSMSNLEPEKATVGMVVPFEWSKPYSDFSKFGLPLPGFLPYIPAQQMSVVNGIKPPDFEEEN

RYLMGAYLNSFIQARRSDLKVAFPREFEEWQQYHMLCLPSPLNSTSCDMIHVYTTFWSQALTYMEAGG
NVYSTFSGDSAIPEMQSLFGASLSDHKVTEDVEIIMSKDFYGLKEGDTFKYKADISNFRHWGAVFKVY
GGRVIAKDQDGDPAIIIFEKGKGKALICAYPIECYLAATPQIYERGDETYRLYQAFANWVGAQSLFST
NT
>SOL_1_150_cov_4.122271_N_333507
MSMPSRGHKLTRRQFIWGAVAGAAGVAAGSLGGKAGLASANTAGSGAMEKQQPRGGGGVPRYGRWHAE
FDPRTRAERVEFTGPGGIAEVRPTFQHLPADLVYDDHGYETSAPRAEQVLAVRFTPTEVGRYHYRAMA
GEEVVARGELQCEPSDHPGYVQRSSRDPRYFAYSDGSPYCAIGLNLCAPDGGWGGKTLGAGTYRRWFR
QLSENGGNFARLWVSARYFNAQGEIAGELDPAAFARLDAVLDLARQYGIRLKLCLENFRSLDPALSSQ
HLALKHPADGRSPANMDEWFQGDAWQQLWWKRVNAYLARYGNDPTVMAWELWNEINCCVTSDWSVQRE
WTRRLLPMVKAKSPRNLVTNSIGSFDMERYQSWYDDFKMEEMDFQQVHRYLDQGAPWDICHYDAPAFS
KDAVERARRPDRPVLLAETGAVNDGHSGRFRYYQADHRGIIFHDTTYPAFFAGAAGTGHIWHWDEYVD
NKNLWGAYRPFADLVAGLKLDEEQFRPFDLTSGAVW
>SOL_1_150_cov_2.534842_N_356481
YWSAVSVHPYRQSPPETAAADYARLRRLIERYAPAGKPVPILSGEWGYSSAWANFTEEKQGKYLPRQW
LTNLANNVPISIWYDWHDDGRDPKEAEHHFGTVLNGYFAGRERPYDAKPAYLAARTLATALAGRRFNK
RLAVGSADDYVLIFSDARCDGVRLAAWTAAASPHAVVIPAGPGRFAVTGHTGDALPALAADAAGLPVT
LTDSPQYLAPEGPNDLLRVAAAWESAPAAVRMPARRSATVALGLANPLARPITVATGRGAAAEVGPGE
RILITTTFDLMRTADPAPVRIECDVKGLGRIAQEMAVSATNPLRLSLVPPAGANLEVRVENPSGEPLR
AAMRLTDLDGLKPAAARKPFQIKAGDRETTVRFPLEGPAAAACRFGACVEDAAGEAIAVAPAVSFAAV
DDFSRYTAETLPQAYRVVGDGDAQVASTQTASRASPPDGPPAPAVATLKIAYSCEAGWKFVRLVPQSD
EMKKIEGRPRALGLWLYSDGTGNSPRVRFTDATGQTFQPASDPMKWKGWRWVEFRLDDPHAGHWGGAG
DGRIHYPIRWDTLLLIDNVGRQKVQGEVYLAAP
>SOL_1_150_cov_3.930261_N_361291
MKNNRREFLKKVVVGAVANVIPAFAQQPEETKKISISPAQTLLPRWRGFNLLYLFTKQGYSKPIEDDF
RWIADLGFDFIRLPMDYRIWIEGSDINKIKEEPFENIDRVVEWGRKYKIHTCLNFHRGPGYCVNQSWL
EPFNLWKDQKALDAFCFHWELFAKRYNGVPSSAVSFDLINEPPAPSETSTSSVGSKMSRADYERVVRA
TTKTIRDADKQRLIIIDGLSTGNDPVPELIDLGIAQSCRGYIPPQVSHYRANWVDRDSSFPTPIWPDK
EGKTHHWDRDRLQKHYQLWADLAKKGVGVHCGEGGAYSKTPHDVFLAWWRDVLEILTGFGIGWALWNF
RGSFGILDSGREDVQYEDFHGHKLDRKLLELLKEFK*
>SOL_1_150_cov_2.797445_N_362133
MVKQLAVIVVFAASVCVAGEKPDAFEINKLIGRGINIGGALDSPTKEGEWGVTLQEEYFQIIKDAGFN
SIRLPVRWDTRAANEPPYTIDPNFLKRVDWAINNCLSRNLPVVLTTHHYDELYSNPDGHKDRFVAIWK
QIAERYKDYPCTLIFDPL
>SOL_1_150_cov_2.797445_N_362134
MSKSLKLSIFYVAAFLILVGFVSAAEPGSKPAKPDAFAMNKLLGRGVNIGNALEAPKEGDWGVKIKEE
YFDIIKQAGFNSIRLPTRWSGHALTEKPYTIDPNFFNRIDQVVNFAISRNMPIIVNIQQYAEXXXXEL
YTEPMAHRERFMALWKQIAEHYKDYPDTLLLELFNEPDDALTPAMWNEWLKEALAIIRKTNPNRTIVV
GSANDSWITYLKLLELPEDDRNIIVTVHHYFPHNFTHQGAPWMTPEKVARSVEDMKFIHQDLNSNGNS
DYNTWPGTKWTGTAEEKKAMTDIFDIGAAWGKEHNRPINLGEFGSYKKADMESRARWTKFIADTAAQR
GMSLMYWEFCADEFGLYDRQTKSWRKELLEAVIPPKQ*
>SOL_1_150_cov_3.577160_N_366319
SDKKGFIRIDTNDHSLGCARDKAFLSYENGDTRLNIGQNIGWNAGEVYSWNNYITKMHIAGENWVRLW
MCRYGGDGGVLLEWKNGTYSGYFGGAGKLSMQTAQRLDRFVEIAEQNDIAIQLTLQHHGQFSTTVNPD
WNENPYNIAAGGFLNNPAEFFTDPNAQRLTKNKYRYIVARWGYSPAIFAWELFNEVQFTNGWGSDRAS
VVNWHKEMAAFLRSTDPFKHPITTSSHGSGFENIWGLADINLIQVHYYGNDTVRYFEQTARGLADFNK
PVLMAEFGAGSTAGVDNPESNPNGLPEPYSTQMREALMLHDGIWSSFHVKSSAHLWWWDCYIDPFDLY
DEFTALSVYAGNENLADYNLSKAARAVSGAEALYANPVLSDFWAVSAQTVFTLQDDYFPGMENLSRWL
HGSSKTAYRSNPTFNLNMPTDGSLKIHVESVSAWGNNRLRVLVNAVQVFSSSYANGSSNFIITVPLSA
GEQSVKIINSGQDWFNISSYEFAPNNVAPLDSIGLSSNQRTFIWIYDTGSQYGKTAHGVFHNETVSVK
GLDDGHYVVDVYA
>SOL_1_150_cov_2.457944_N_369102
MHSSYYIAEPFDHTRSAGTAATPPDSLDIGSERMIASGKPAENGLQFPGQGRVGREMGGKTAKHGYTA
RDGWFLDRLGRHTLLRGVNLSGSTKVPCSPNGATHLGVDFQNWADVSFAGRPFPLAEADEHLSRIAHW

GFNTLRLLVTWEAIEHAGPGRYDEAYLDYVREVVKKAAEHGLLVFIDPHQDLWSRWAGGDGAPFWCFE
WAGLRPERFVEAQAVELNAVDWLNNYNRVPTATMWTLFFAGDTFCPELAGV
>SOL_1_150_cov_3.350158_N_372674
MNRRDFLKTGGAFAAISITGLKAQAMSKSHKPIKSFSQYRGFNLLAKFGEWGPRAKFEEEDFEIMKEW
GFDFARIPMSYWRWASKDDW
>SOL_1_150_cov_2.423151_N_378270
MKIADKVEARQAGSGRPSAVVCALLRNGETRRVAAKWLSEIGRAMLIAVLGGVACSTAWADAVSENTA
AREWAIKIGWLQGIRKVELIRSDVISVTLDAGITGAIVHPTPEYQGKSQDVIAGCGYATDFVKPAAFT
ITSATDANYNAETSPADVSMNSLPLKNKAGGGKINGVAAPGCAWTIFYTPLYQYEYYLFLPKPLKSGA
SYKISVNTTKPKTPGVRYDSLFAYDESTTASKVIKINQVAFSPVAKTRYAYLGWWAAGKGPVDYSSLS
KFSVVNEATGATALTGAITLRAVPEETMVKLTGEKVYEMDISALRPGTYHICIPGFARSETFAVGGNN
VYALYYYTMRSIFHQRCGQEFGPPYTWAVRPACHTKFWEAGFPVQGAEFMN*
>SOL_1_150_cov_2.860180_N_378866
AKNCINLFIDSHQDVWSRFSGGDGAPGWTLEAIGMNISNIHRTGAAFLHQNNTGPLPRMIWITNSTKL
AAATMFTLFFGGNYFAPETKIDGEPVQEYLQRHYIDSIKQIAMRLKGLPNVVGYGTMNEPLCGYIGWR
DLNKPKAVVEVGAIPSPFQSMLLGAGIPQEIDVWERRITGPKLVGKRILNPDGFRIWQEGHDCIWRDN
GVWDLDERGYPQLLRPDYFDNSIDGQQVNFSNDYYLPFINRFIESIKSVHPKAIFFIDEEELHREVPV
FVTTLDRVVYPPHWYDGVVLFLKLFNSYLGYDEVADKIIFGPWSIRRSFKEQLARFRKHAVKYMANAP
VLIAESGIAFDLNNKKAYRTGNFKAQVRAMDRTLRAFDDNLLSYTLWNYTSDNTNEHGDMWNNEDFSI
FSRDQQNDPNDIHSGGRAIEAIVRPFAKATAGEPLQMSFNISSKVFEFTFRHDPLVSAPTLIFIPEYH
YPRGYDVIISDGNYEIDPKEQILIYYHSQTREIHRVQVRPSK*
>SOL_1_150_cov_3.492498_N_382411
WRHLITTSYAGPQGDPAVDSLPELDFVQSHDYGSKDMEKAFGEHLDAKPAASDRPHFHGEFGIDDGKK
TAELDPTGIHLHNALYACVGQGQAGTPMSWWWDSYIHPRNLYPIFGSFARWIDGFDFVAQKARRAEVQ
VTAEDLLLKEPTTLKPVKGTWQPADFNQPLTAQVSRDGLMSYNVPLSDLLHGIGHHKPLHNPVTFELD
VPEAATFGVEVKGISGFGDAILQITLDGKLALEKQMPLPQNPKDVVHDYDGVYSIALPAGKHTVKVEN
LGKDWLTVATYTIPWLTVATRIGGPLRALGVVGEGRALLWVQNKLHTWASATAKDFKPTPVKGARLHV
LGLRPGRWAIERFDTVKGAVTKSEESVVGPDRKLTIPLADITWDAAFRLEHVGE*
>SOL_1_150_cov_2.222295_N_384078
VRLPMHYNLYTLPVEKEAVSGENTWLKEGFAYTDKLLKWCAKNQIYLILDLHAAPGGQGNDRPIADVD
TTRPQLWESEANQQKTIALWGKLAERYKNKEWIGGYDLINETNFKMEGNEPLKKLFLAVTEKIRSVDN
NHIIFIEGNQFANDYTGLTPPWDNNMVYSFHKYWNAATIETIRKYLDMRDQFNIPLWMGESGENNNKW
YSEVIQLLESNNIGWAWWTIKKIGSESGIMNVTKPQDYQKVIDYWAGKGSKPSVEEALQTFMELAENM
KLENCKVNYSVLNALFGK*
>SOL_1_150_cov_4.253447_N_384358
MRNNAASIFSRDPDRNTIFSIHMYGVFDMASEIQSYVSTYVNNGLPLVIGEFGWNHSDGNPDEDAIMA
TAQSYGIGYLGWSWSGNGAMGLRPFR*
>SOL_1_150_cov_2.553513_N_384442
MRYILLIVLASILILTGCKKPESVTVQPLQFRNVYLDDEGIIRWSDNNEEVALFGANYCLPSASDYRA
AGYITNDRKKIIDQDMAHFARMGWDGLRLCLWGDWENSDSLGNLVV
>SOL_1_150_cov_2.844667_N_389003
SRLLTLPPGRRYSSAMDRRVNSAPRAGIRHNAKKEISIVPVLLVVLGLIFAGPGPPSPSSSSLWSEDF
ESPDAANRWRYANGPEFPGASGSFRIVEAPVHGGKRAGRLEYDFSKGGVYVEAWTVFPAPIPGREVSF
WASLSTPGTEVRIRLTDETGQTFQSRFPAPFVAAWTEFHVRVGDFEEFWGGANDGVFHGPLARISVLA
CRGLDRFPKGALVLDDIRASPSAEAVTDPFGVDARPPFHTGLVSDLVGVESNFVTHFPLDTRQIELAK
AAGFGFVRSPLHWDRVEKTKGRYDFKAWNDLVRALSSSGLGSYLILCTSNPLYYDGPGAYDYMWGPRT
EATRRGFAAFAREAARNFAGRNVVLEVWNEPNIQNFWHPKPYVRHYNLLAAAVLDAVKGAGLKVPIIV
GSTSTVDLPYLETLFKLGGLARVDAVSLHPYRATPPETFYPEFATAMAAVRADAGRTDLPFYSGEWGY
SSTWFGGRTAAALNTQALHAIRLILTDLYIQYMEEQPGQPAP
>SOL_1_150_cov_3.272023_N_406240
MRSVATSILAMILVAGVSAWAAAQQPQVKGEAAPKTGPNGTPMGLGFNVHITGPDSDWDAIKAAGVTL
IRADFTWGAVERTKGEYDFSTYDRMLDALDKRGIRVDFGLEYRNPIYTNPETTEEGRDAYARWAAASV
RHFKGRNVVWEIWNEPNVGFWHGNKGEKMNSAEFAAEYVALVKKAVPAMRAADPDCYILGGSVSCLWR
DSFQWLDAALKQGLLQTGINALSVHPYGFPRPETCIDANQPGTVAGQGYGVLREMMAKYNAPKDFPVL

NTEVGYPIGKKVTLDQQAMLFVRQYMVDQMCNIRMTIWYNWDERDAANHRVRSRGPEPLPVYNACKNM
TAEMAGYHFVERLKVGSAVDYVLAFENGSKGRKIVAWTVPQARDESQDKAVAHDVGIPTGSTGGPVAV
RDLYGKEVQAKAAAGTVTVTLTGSPQYIDL
>SOL_1_150_cov_2.608511_N_408402
PHTKEETRHVEVVKALVKAIREEDPDRLIVADGKDVGRTPIFGIADLGVVQSTRGYDPMSVSHYTARW
VPKESFESSKAPTWPLKGDDGKVWDKAALKAKLINTWMPLVDKGVQVHVGEWGCYNKTPHDVALRWMR
DLLTLWKEAGWGHAMWNLRGDFGVLNSGRTDVQYEDYKGNKLDRKMLELIKEY*
>SOL_1_150_cov_3.517844_N_417052
MNLRKVFLWISMVGLLAYGAEAVAAKEAIVLNEKEYFEGPGFSFLVFHNNYQVGFQGGLQMIQNGERI
LDSGDLLLTPKSGGLEGPELRVLRRVVDRERSTATVYGEIGGWDTGYQLICRTDGERIFITLKLDRPI
DWSKVERAGFRICLYPGTYFSKSYQGDSGSGVFPQQYTGQMILSGPTKTLRVAQEDLLHSFAMSRADG
ALLLIDP
>SOL_1_150_cov_3.542561_N_422545
RKMGKEQAMPPPALLQQIMGHVQFLLGAVPPVGHMLRDVHHGVPPTGENPTVTSTQQSICASSNCQER
SLKVKVVSALALALARFTAAAEMPEVVHVGMVAPDIISVTVQAGRAEYGQQVPYQPQPNDRVDRKTHQ
RWVHRDGKFIGALVGKDEKILYTPDRAVGPKLDTAWADMPAIYAIRCAEDPNYAGDGRAPVAVHRKSR
PTDFARVGPWQMDAPMEHSLYLRLPRPLKAGKRYAITFRDSRLPDQSFVYDPTRLRSEAVHVSHLGFR
PDDPAKVAFLSCWMGSGGGVTYKEGLRFDVLDANGKSVFQGKVALSRAAADPEDAYKRNYNGVPVYEM
DFSPLKAPGTYRVAVEGVGCSHPFPIADDTWRKAFTVSARGFYHQRSGIELGPPYTTFKRPRPFHPDD
GLKVFLSNCGLMDSGNGLNRKDNNFGNLNKGRTDQLVPNAWGGYMDAGDWDRRIQHLVVS
>SOL_1_150_cov_3.136701_N_423852
MIKLFTVILVSLITTVTITPACKAADEPNAFAINKLIGRGVNIGNALEAPNEGEWGVTIEEQYFDLIK
QAGFDSIRLPVCWPAYAMNKEPYTIDPNFFKRIDWAVSNCLSRNLPIIITIHHYNDLYENPAGNKDRF
LSIWAQIAEHYKDYPSNLIFEPLNEPHNNLSAGEWNKLLKEAIPVIRRSNPTRIVVIGPANYNDIYQL
ESLELPKDDSNIIVSLHYYLPYEFTHQGAFWVPDANAWLGIKWTGTNEEKNAVIRDFDVAAGWARKNK
RPICVVEFGAYEKADMDSR
>SOL_1_150_cov_3.136701_N_423853
ILKEMIAAIRISNPHRTIVICPAGLLCIDNIHLLKLPEDDRNIIVSIFYYSPLEFTQQGATWVKDSNK
WLGTKWTGSEDEKQKIVKDFDIAAKWGKENNRPIYLNEFGTYEKAGLESRVRWTKCVAETAAQRDFSL
SYWEFCSVFGLYDLQTKSWRQPLLEAVIPTKQ*
>SOL_1_150_cov_3.382622_N_432501
KWSREHRRPILLSEFGAYEKGPMESRARYTAHVARTAESLGWAWTYWQFDDDFIVYDLDQDQWVEPIR
KALVP*
>SOL_1_150_cov_3.328025_N_447941
MSAGKGNIKWILIFIFISCVIPLKSQAPFTRGVNLSGWFQVSNPGEIQYTKFTKKDLVNIRTLGCDVI
RLPIPMHDMTSGSPDYILDPLYFSFLDSVVTWCEQLNLYLILDNHSYDPDGDTNPAVADILVKVWMQM
ASHYKDRTKYILYEILNEPHGITTSAWGTIQNLAINAIRTYDTKHTIVVGGSNYNTYTELKNLPVYTD
TNLLYTFHFYDPFMFTHQGATWVNPSMAPLSGVPFPYNPAEMPACPASLKGTWIESSLNNYHSDGTTS
HVKQLIDNAVTFRDSRNVKVFCGELGVYIQNSDDADRCYWYKIVREYLEEKNIPWTTWDYKGGFGLFN
KLSNEFFEHDLNVRLLDSLSFNIPPQTPFSVLPDSTGFMIYTDYVEQNIE
>SOL_1_150_cov_11.980048_N_448609
MVVKFRVLVLLLFVSVSTAAAPARPAPQATQAPGKEAGPSAGDQWDDLKLWYSRPAMFWTEALPVGNG
RLGAMVYGRTDIEEIQLNEDTVWTGGPYDPSNPEALTALPEVRRLVFAGKFREAQELYGRKMMARPYN
QQKYQPLGDLRLSFTGQGRPTDQNKQLSRYRRELDLDTAIAGVSYTIGNVSYRREVFSSPIDQVIIVR
LTADKPGKVSFRAGLTGRKNELSLDDTYAAITEIDGKPAASPSDEFFRTEADAPDTLVLRGRTATYLG
IAGRVEYQARLKAVAEGGSLGTEGDSLVVSGADSVTLVLAAATNFVNYRDLSADPEVRVTEVMKAVAG
KTYDRLRSDHVAEHRRLFRRVRLDLGRSDAEGLPTDERVKAFSKTDDPQLAALVF
>SOL_1_150_cov_3.917468_N_450153
FEGFKSMGGAPDWPAAGGGKPWFISYYLDEMKKASDANGRRIFDILDVHWYPPEDDGNGHGICDDRED
AQTFNARMQAPRTLWDTDYVSPHETYPDGGSSWVNKWWNAQFLPILPRFKSDIATYFPDTNIAITEYT
WGPSTQWATGIATADFLGICGKYGVYMTNYWGEGGYIDTAVKMYRNYDGLHSTFGDTNVPATMSDKVN
SSIYASVFASNACELHLIVINKNQTDNITGTFNINSPQNFLSGRVWKFDNTSSTISETTGIYTITNNS
FTYTIPKTSVCHIVLRVIPLNVSITSPLDGDMFTSGDDIVIEANASAVNGFVTKVEFFQGSTKLGEDT
DSPYSYTWNDVNVGRYSLTARATDNNDGTTTSTAVNINVFSGDATGFILREWWTGISGTSVSNLTSDI

NYPTNPNGRALLTSLEAPTDWAENY
>SOL_1_150_cov_2.390561_N_455999
MWKAMGQSTLTIGSLLVLLTLAKVTFGFASSVAAEPNAATKIPDPFAVNKLIGRGVNLGNALDAPKEG
EWGVTLKEEYFDLVKQAGFNSVRLPVRWSAHALTEKPYIIDPNFFNRVDWAINCALSRNLPVIVNVHH
YMELYTEALAHKERFMALWKQIAEHYNDYPDSLLLEILNEPDDALTPEIWNEWLKEAHSIIRQANPTK
TIVIDSANDAWISYLKLLKLPEDDRNIIVSVHHYFPLEFTHQGASWVTREKIASFVKDMEFINQSVPS
WVTVGDSNAWMGTK
>SOL_1_150_cov_3.681255_N_461349
FPRQANGPGMFDKKGALVAAKPMAYGKQLVVAPEDENLRLTIRSGTGDLQLIDGRYQHNNGWFVVRSL
VAPGATTKAIEWVITPNVINGWIGDPVIHVSQIGYHPAQQKTAFIELDKNDPKRENIELVRVGTDGKQ
QTVISQKPEEWGRFLRYDYLKLDFSSIKNPGMYLVKYGKQQSQPFRIASDVFERSVWQPTLEYFLPVQ
MCHMRVNEKYRVWHGLCHLDDALMAPVNYNHFDGYIQGGSTLTKYKPGEHVPGLNIGGWHDAGDYDLR
VESQSGEVYILTQSYETFNLEYDETSVDQHSRIVEIHQPDGKPDILQQIEHGALSVVGGYRNLGRLYR
GIICPDLRQYVLLGDGVNMTDGLIHSAEVKSGEKTANHSSLTDDNRVFTEDNPGRELTVAAHMAAASR
VLKGFNDTLSAQCL
>SOL_1_150_cov_3.600334_N_466061
IVDGLSWGQEPCPEFIDLGVAQSCRAYSPSALSHYRASWVAGSEQKPVPVWPGVVHRGEVWDRARLEE
FYRPWAELARRGVGVHCGEGGCFKNTPHDVFLAWFRDVLEILTAHGIGWALWEFRGSFGVLDSGRTDV
AYTDWHAHALDRALLDLLREF*
>SOL_1_150_cov_4.921569_N_473592
MAIENLQFGQVPSSFTKGALTVAVILAAALLPDSSRAAERSQMQLIAVAPDGQGFIEQDSGRPYIAFG
TNYYDPDTGWAPKIWRQFNAQRVRQHFGVMSELGVNCARVFLTAGSFQPDPQRVEEQALEKLDELVKI
AGETGIRLILTGPDHWEGVPSYWQPDRFAGKSALDALQRFWDVVGRRYKGEPAIFAWDLLNEPELPWF
VQGWLGQWNAWLQQTYRSWDELKAAWGKELTEADRWGEVAVPRNQPDLGNPRLRDWQRFREHLADEWV
RCQVEALRRADPTHLI
>SOL_1_150_cov_4.375427_N_473909
MDMLHVKSDRIVDELDNPIWLRGTCVGGWMNMENFLDGYPGAEHQMRAHLAQELGESKARFFFDRLLD
HFFAEEDVAYIKSLGATVVRLSLNYRHFESDAYPFEYLEPGFQRLDQAVGWCANHGLYIILDMHSVQG
WQNTDWHCDNASRHVLFWHDKCYQDRFIALWQEIARRYKGNPTIAGYNIMNEPLVNTPFGRFRENRTP
DLASYYRPDWERINRIYRRAVEDIRQVDPEHIIFLEGDYFSNLFMGLKPPIIENLVYSSHNYNTGPEE
RWDFERQRSYFLGLEGTQYTQKYNVPLWVGEFGTGYAFPEEEQRLRLQALDAQIRVFNQYSTHWTIWN
YKDIGAMSLVWLHPDSAYMRAIRPVLEAKRALE
>SOL_1_150_cov_3.754961_N_478259
MCRVKSPSLFLFIIAVSLVACSCPRLSAADEPNATKFTPDPFKINKLLGRGVNLGNALEGPNEGAWGV
ILQEEYFQLIKDAGFNSIRLPVRWSAHALNEPPYTIDPKFFGRVDWAVKNTLSRNLALVFTMHYYNEL
YSDPNGHKERYLALWKQIAEHYKDYPETLLFELLNEPQGSLDIAGWNVLLKEALAVVRRSNPYRTVVI
GPANFNDIYKIKTLELPKDDRNIIVTFHYYLPYRFTHQGAPWVPDSNKWLGMKWTGSEDEKRLVVKDF
DLAANWANQNNRPIHLGEFGAFEKADMDSRARWTKCVADTAVEHGFSFSYWEFCSGFGLYDRRTKSWH
KELLDTVIPPAASPP*
>SOL_1_150_cov_3.390964_N_481085
DDLYDLLRTVVDYCKLKDLYVIIDWHYVANTYDHVETTSEFWAYMAPRFANDSHVLFELFNEPINDLN
DDWIFNANDTADWLSVRSDMQTWIDIVRDYAPNNLILVAGAFYSQVIGPAASYPLTGDNIVIVSHIYP
GHFLNWCWSNGCGSSSGSYRNEITTCAAVYPVIMTEWGFSQSNNPDPGDLLNGTISNYGQPLADFREL
YGIGHTAWVASYNWGPPMFWTDWTLRCGEGEMGCFVKDKLYEKRNSDQPTPNFIDFADFTTQWGRTNC
SASNVWCSGADFYQDGSVLFDDLQAFVDDWLFLE*
>SOL_1_150_cov_3.114035_N_484947
SHASGALGISGDKFTIGGKPTFLLGVSYFDALGWSKADLDALHARRFNLIRIFLDWSILATKDNTPSP
LVPRGFVNPDGSLNNAESLLNLVRACAARGSMVDVTILNAIYDAANPLSKTLNMQSRENAVRNAVRLL
KQEPNVFFDVCNEHDVAWNGKATTLTHAEVKALIRAALAEHPGAILTVSSSGGHLPNARNSREELDAG
VMLISPHFERTPDWFDRTDDRVKSVKNDTRSAGRNVPVYLQEEQRRGWTKVSPPKSEFLQAAREAVSS
GAAGWVFHTHAGFDLRSSNFLANLDPVEREVLDSLGSEVFRTPPPGKSRVLRVHPTNPRYFTDGTQNP
DGSLRAVYLTGSHTWANLQDNGLTVGSPTIATDPPPAFDYDAYLKLMQASNLNFMRLW
>SOL_1_150_cov_3.231317_N_490650
MSKRTGISALIMVSLLVLVTFALTSCMSEAEKAKRDQDTRKAFEMNKLLGRGVNLGNALEAPHEGEWG

```
VTLQEEYFQIIKDAGFNSIRLPVRWSARALSEKPYTINPSFFKRVDWAVNCAIKNNLYVMLNIQRYDP
LTDDPNSYHFERFVGLWQQIAEHYKNSPDLVVFELYNEPYKALTPELWNDLVKKTLPVVRKTNPNRTI
VIEPTIIIEPNFVVCLDKLKIPKEERNVIVSIHYYTPLEFTHQGTPWMGERSKAWVGTKWTGTDAEKK
VVTDFFDAAAVWGKDNNLPVNLGEFGTYKKVDAESRVRWTKFIAESAAQRGMSVFYWDFCAEFALY
>SOL_1_150_cov_2.735950_N_491854
MKKLQKSYGSLIKIALLFSATIASTACGYYTTQGQDIIDCKTGQKVLLQGFGLGCWLLPEGYMWGIRE
LNRPRHFEKAIVDLIGEQNAAEFWRLYHDNFVTEGDIKAMKEWGVNSVRIALLASMLQPRDSQPDAPP
YVYSEEGFKYLDNLVRWCDKYKVGVIWDMHGAPGGQNAENISDSDGVARLWTEKEKYWPLCIDLWYKI
AERYKTEECIIGYDFLNEPLLRRYEGININLLRELYVELTKKVRTVDKDGIIFVEGDDWAQNFSMLEP
LNWDEHLVIAFHSYPPTSSQNGLKKWDDLRNKYNVPLWHGETGE
>SOL_1_150_cov_1.678733_N_497921
MXXXXSWQLANEPRPGRDNELGKSNLPAFYRWIDETAMFIHSLDTNHLVCTGCEGTVGTLMSEKIFVE
AHRTASVDYLNFHLWAFNWGWFDPERWEETLPS
>SOL_1_150_cov_1.678733_N_497922
MKPQRIPLLLVTCSLILYFSSCAILSLVQGREDFIRIRGTQFIHKGAPYYFAGTNLWYGGYLGSPGST
GNRPRLLRELDALCENAVMNLRVLAASEESYIRRSVRPAIQSAPGVVDDSLLQGLDYLLAEMAKRDMH
AVLYLGNYWEWSGGMAQYNVWTGRVGADPENPSQGWGAFMDSSASFYSNPKAVEFHRNYXXXXVLATC
QRTAAGTG*
>SOL_1_150_cov_1.706740_N_500477
NISLILCLWSFDMLQPNALAKNHERNKQLLENIENIQAYIDNALIPMVEALKGHPAILCWEIFNEAEG
MASDIEWGGWTATTTSFEPYIQRFINMTAGAIHRTDSNALVSNGCWAFKVMTDIKTTRNNKNLYRNDR
LIEAGKDSLGVLDFYMVHYYEWGGTEYSPFHHPASYWQLDKPIVVGEFSARDPYAGIDSKAAYDSLFN
NGYAGAISWTWTGHDSNGNITDATPGLQYLFNNYPDDISVDYKSRAINFLPQVPKEKPLQNK*
>SOL_1_150_cov_2.814273_N_502223
DLYAIIDWHYVGDDTWTKVTQTSAFWSYMAPRFAGDSHVLFELFNEPLNTSPGNEAQDWATCKADMQT
WIDIIRTSAPNNLILVAGPSWSQQIGPSAADPFPTSNPHNTNIVMVAHIYPGHWLSGSQSWYLNHITT
ALTRYPVFMSEWGFCQTASYDLLRGTITNYGQPLWDWREARKISSSAWVTDYAWEPPMXXXXCFIHTG
TTTSREREKRRLLQHRRD*
>SOL_1_150_cov_3.182825_N_506009
VNTTEMWQAESFDAATIDRELALAQDLGLNTTRVFVQYLVWKHDPEGFRKRLDQFLAIADKRGLSTMF
VLFDDCKFSGKEPYLGKQDDPVPGILGSGWTPSPGHVRVVDKAAWPDLEKYVVDVVGRFASDRRVIAW
DLHNEPGNSGMGNKSLPLVRACFGWAGKAKPSQPLTIGVWNGGLGDLNKAQVELSDVVSFHNYGNLDA
VKGQVAGFKAHNRPIICTEYMARTIGSFFKTHMPWFKQERIGCYSWGLVNGKMQCQYPWSSKKGDPEP
KLWHHDIFRKDGTPYDPDEVAFIRKFLRKQE*
>SOL_1_150_cov_3.008499_N_515334
PVSYQHFDLYAQGPTTDTPYQPGEHIPGLNIGGWFDAGDFDIRTPSQYQVVLSLVQTWETFHVTRDET
LVDQKIRYVEIHHPDGVPDLLQQIEHGTLGLIAQHRAVGHAIPGIIEPNLSQYTHLGDALTITDNLVY
DPNLADHESNGFASGTFDDRWAFTSKSTPLNYGSIAALAAASRALRGYNDKLADECLETAEKVWIEEH
GHEPNLFRFGNTTGGQVEPEELTAAIELLITTKDAKYAKRINELLPIVEKQFGRFAALAVRAMPYMDD
SYSKKIETLARAYKKDLDKFYEQNPFGVPISTGGWAGSGWVIYFANNCYLLHKAFPEIIGSEYTFRGL
DFLYGCHPGSDISFVSAVGTRSKKIAYGNN
>SOL_1_150_cov_2.190665_N_537614
IGFNEKDEFNPGMIYEVRKWHNDEVVYSGNLTEWNKGAVDFTSGDKGWWFDFTPVNKEGEYYIYDKQQ
QAGSYKFLIASNVYKDILKVVMRTYYYQRLNSPKEKPYTEDPWTDAAAFIDSGQDKEATYVYDKGNAA
TAKDLSGGWMDAGDYNKYVTFASSPVHMLLTAYQQNPGVFTDDYNIPESGNGIPDIIDEVKYELEWIK
KMQHDDGGVLIKLGNIDYNSSNPPSADKRPRYYGPECSSSSIATAAMFAHASLVFSQFSELAGYTSDL
KVRAIKAWDWYKNNFRSDTCDSQEIKAGDADLNLEKQDEMEVVAAIYLFVLTGDERYNEVVMNRFYIT
SPFYDIYSYLYFSY
>SOL_1_150_cov_2.199593_N_549154
PDFPESLYLVYPINNSIYDKEALREYFNKMHDHGVTVLRVWGEAPDHGYRYLLLEDPVGVFNPAFARF
FDDVFELAEDYKIYILLTPYDTFWHHFSWSYYPYNVANGGPCHSESEGLTTDKCFEYQKARMKWFVDR
YGNSDYLFGWDIMNEIDWTWGGQSAATIRTWTDRMSSWLIQYERQKWGKNHIITVNTAGTPWEEDLIY
SVFQHPNVDFATTHMYYSPIANPPDVIAPAVTVNEIVKLELSKFAVGDMRPYFDSEDGPIDGELPEAF
DNEYYHNMIWAHLASGGAGSNLRWPFRVPTGPTDGMFDSDLAMSQIIKNIKWSKFSSVNIDNNVTVTN
```

TGVHT
>SOL_1_150_cov_1.868448_N_553457
NFADFLSKMASRYKGRIQAYAVWNEPNLAREWGNKPPNAGEYTQMLKKAYQAIKKADPNAIVVSAGLA
PTTELSQRAMPDTQFIETMYKAGAKPYFDMLGAHGAGYKAPPDMDPGAVANDPNYYNVGDPNCPGPAC
RIYCFRHVEDLRKIMVANGDVDKRVVVLEFGWTRDERTNSPYHWHRVADQFVQGDYMVRAYQYAKDHW
QPWIGVMSLIYMPDVKWTQNDEQYWWSVMEPSPIDQLYLKAPYVMLCIYLHKERGLGSCPYAPQ*
>SOL_1_150_cov_3.447531_N_553705
YYTTKGKDIIDQRTGERVILRGIGLGGWLLPEGYMWGIRQLDRPWQFEDAITELIGEEKAIEFWRIYH
DNYVTEQDFSAMKEWGLNTVRIPLLASKLQPRKGQPDIPPFIYSEEGFRILDSIVKWGEKYHIGVIWD
MHGAPGAQSEENIADSDGQARLWTEKDKYFLLCIDLWYKIAERYKDYECIVGYDLLNEPLLRRYPHVN
NKLLREIYVLLTDTIRKIDSTGIIFVEGDDWAQTFDILEPLDWDPHLVIAFHSYPPTSNAKGLERWNE
LRNKYNIPLWHGETGEQGPPYEINILSTTFLEQANVGWAWWTHKKFENQSQPWIVTTTAGFNKILDYW
K
>SOL_1_150_cov_2.395812_N_562495
VTGLGYKPPMRPHDRRSGADRVREPWPGYIVGGGHSATGWHDVEEDARTNEICINWQGALVYALAAFA
DGTPR*
>SOL_1_150_cov_2.467505_N_562977
DGRFSVLFNGERRLSAALKRLPDFPDWGKGRHYYLADFSSLTETGRYLVRAQDDSVRAASGTISVAKD
ALFRKIAPALVHYFRESRWLDAGDHDIGVYGTDRRVDAWGGWKDAGGDDGKYLSHLSYANFMNPQQAG
LTAWALARSYDASPDRFRSLGLANDIVEEAFWGADFMHRMLSPEGYFYMTVFEGWGRRDAPRVITGYV
GEEGIYTGDYHAAFREGGGMAIAALSRAARLARSTGKSGAFAGSVYLADAERAFAHLSSNNTRYDDNG
KENIIDDYCALIAAVELAKSTEKLEYQQAADARAASLMARITPGGWWRSDDGERPYYHSIEAKM
>SOL_1_150_cov_2.926393_N_564374
PGVTEPDYPYFKGLDSYGIGDDNHSLYGPIPGFVPGGPNKDYSGLATPPRGATYYERFYRDWNDNSPR
GWYRTKVWELNENSISYQGPYVALIAGFMSAVTPPPPDTTPPAAPTNLTATAIRSSQIDLDWSNNTEG
DLLCYTVYRGATSGGPYNLVAGNVAVSAYSDTGLAESTTYYYVVTATDTTGNESARSNQAGATTPSSA
GTMHVSQIPNGGQKGQNPGRWADVYIVNNVGAAVVGATVTVTCTGWDNGTQSNVTETKTGVTGSGGKV
RLVTIVDTGSMCVTNVTHATLIYDSGQNVVTCIYW*
>SOL_1_270_cov_64.260584_N_3597
MRLFLFWFFTLFLSGLASAKDVTIYNGETVKFSSWSAWDQNGSSIVQLKKDSFSKPNHFRATIKNKNW
WGGVAYVPNNWNPIDLSKASTISLAVKSPNFRQVGVSLFDINKKSGQTVTLNVTPAYQINSISVNELS
SGIDLSKVTAIVFSESLNGSKSVVIDVDDIVASISDTPPPDPTPTPNPDPTPIPSGSTMKTNGRFLYD
ACGQKVVMRGVNQMTCWTDWVGTPRDGLPMFAEISKTGANVVRIVWISDAGANTPAITIAQLDTAITN
AVANGLIPMIENHDKTCMWSTKDVTDVLNWWTTPEMKALIAKHQKYLLINFANEMSAPNKTEYVKEYS
RVLTTMRSAGIHVPIVIDSSGCGQDEAMIMNAGPALIAADPDHNVVMSLHIYWMDQNAARIAKAVNDS
VALNIPMIIGEFASVSTDCSTPILWKEIIKQAQINQIGYLPWSWDNQNACATHAMTKDNNMSFSTLWG
WALELSVTDPNSIKNTSVRSKCMGPAPVPTPTPTPVPSTGGLDLGQDGILQIHAIGDSITSNPGWRCT
IYDALTKIGVKTEFLGTVVDAYPKCVQKKSDGHSGYNTSNVYSEVDGWLGSIPKPELTLIMLGTNDVA
WWTVEPEADVVARLNKVVDKVKLNSPKGVIFVASIPPEGGPNAGTNVPLLIPPNSRDRNTLVNNYNKG
IADMVSKRKDLGDSIYFVDIFPSLTTADLYDGIHPNDVGNQKIGRAFASKITGMLPVKSKKAKMRSNI
NRLP*
>SOL_1_270_cov_64.260584_N_3763
MRCFSIIMMLLFMAGCGSSTKSLFKQPTPSNHQEESIQPKIDDVYDKQILSTTSWRVDKGVILKNDIP
IFLRGISWFGFETNELVVHGLWTGRSIASFLQQVRDLGFNALRLPVSPQVFRDGFASGHGKPKPIDNL
NELLSEAKRLDINVLLDLHNCDYKAGLNGNPIGCNGGLDNWLNTLGQMAELSLHYDNVLGIDVFNEPY
NVSWKNWSNLSAQAARKILAINPRLIIYVEGVGSVDTNTGGLGGPNWGGNLVEAGNNLPDIPVSRLVL
SPHAYGPSVSWQSYFSDAAGFPGNMDKIWDSHFGYLTSKGFVVSVGEFGGRYTERDKVWQDTFISYLV
KKDMRNFFYWSLNPNSGDTGGILNDDWTTVNKDKMDLLKKLF*
>SOL_1_270_cov_10.673595_N_8781
MKLFLFLLFSFFSIYGYADQTIYDGEKVKFSASSTWDQNGSTLQQRTDATGIYLRANIKVVNNWGAAA
YTPKNWQPLDISGFKSVSLMLKANVPLKIKIGAYDANKKTTYSPEYAIGTRYTKIIVPLANTEIDLKN
VLAIVFAIQKTGKYIVDIDNIILISECTIPTPTPTPTVPPTPTPTVPPTPTVPPTPTPTVPPTPTVPP
TPTPTVPPTPTPTVPPTPTPSPNLTIIYNGETVPFSVGSAWDSNGSTLIQANVAAHSAPNHLRANIKN
LNWWGGVVYLPNNWNAVDFSKSDTLSFWVKSSVNTTLSFGLYTTTAGPTVNAAITTNYTQVSIPLNQI

SSGIDLAKVTGLVFATSQAGTTTYVVDIDDIALSGIASTPTPTPTVPPTPTPTVTPTPTPTTPPTTGT
TMKTNGRFLYDACGQKVILRGVNHMTCFTDWVGIARDGLPMFSEIAKTGANTVRITWRTDLPSDATLS
QLDAAITNAAANKLIPMIELHDQTCNWSTAAITQILNWWIQPNVVAMIKKHEKYLLVNFANEMGAWGT
TPTEFKTEYTRAILAMRTAGIHTPIVIDSSKCGQDEVMITQVGQDLIKADPDHNLIFSLHIYWTDQNA
ARIAKAMTDSIALNIPMIIGEFASVSVDCSTPILWKEILKQSQINQIGFLPWSWDHQNACATHSMTKD
ANQSFSTLWGWALELAVTDPNSIKNTSVQSQCFGNVTTATTIKTKIKTVKPKNIKLPAHKVAKKP*
>SOL_1_270_cov_10.673595_N_8823
MKYLCFSVFVSLFLLLTPNQLISDPVSENGYLSIKNSQLVNQEGIPLQLKGMSSHGIQWYGKFVNYNA
IKELRDTWGLSVFRIAMYLNEGGYMSKPELKNKMIEGIDAAISLGVYVIIDWHILSEKDPNVYKNQAL
VFFEEMATKYRNYPNVIYEIANEPNNTNWANNIRPYSIALASKIRSIDTHNIIIVGTNVWSQSVDEAA
NNPLPPEYKNIMYACHFYAGSHGSWLRDKITYAMNKGIAIFVTEWGTTNSSGNGGNNYNEARAWMNFL
ADKKVGWANWSFSDHSESSAVFNPGANPNGGWSDGNLKETGRFVKAAMLLQTN*
>SOL_1_270_cov_11.601940_N_12207
MRLCLAIFLFFIVSFTSLYADVSTHGRLQVKGSRLVDQYGAEIQLKGMSSHGLQWFGNFANKNAMREL
QQDWGQTVFRAAMYTKEGGYLDNPSVKSKVYEVANAAVELDQYVIIDWHILSDRNPMWNVNKAKEFFD
EMSKYYAAYPNVMYEIANEPNGGDVTWDNAIKPYANAIIPIIRKNDPNNIIIVGSSNWSQNVQDPARD
PLPYSNIMYSCHFYAATHGQWLRDRISSAMASGIAVFITEWGTMDSSGNGGINYNETAAWMKFLDANK
ISWANWSLSDSGQTHSILRSGSNVNGGWTDANLTASGKLVKSYMLPSP*
>SOL_1_270_cov_28.606756_N_19029
MRLLCRLLSLCVLLVTSCYQAVEAKELPTFSGTNQYYLFYKPQEMVDDFFMRAKQLNLTVVRTWAFCD
GPSHDGYCFQPSPQSYHEPSFVKLDYIVAKAKKEGIRLILPLVNNWENFGGIPQYLKWFNLYNHDDFY
RDSRTKETYKAYIKHLLNRRNTITGVLYKDDPTILAWELGNELRSYDLPVFYTWVEEMARYIKTIDPN
HLLTTGSEGAIATDVYQTHKSPDIDFVSFHLYPEHWGFDLTRSNQYIRDHVKIARSLNKPVFMGEFGL
RDKGKRRDAFQGWYQIIKEEKIEGAAFWLLSGRQPDGSLYPDYDGFTVYVPESTDVNDVIKGYSDYAK
TIPINQSGGDL*
>SOL_1_270_cov_18.711169_N_21131
MLKVYFLFFLLLTSCGEKMTSNSISWLKAKNSKIFDESGAEIQLKGVSSHGLAWYADYYHKNSIKYLV
DNWGIKVFRAAMYTEEWGGYIGNRSIVKKVDEIVEAAIELGIYVIIDWHILRDGNPTTHEKEAKEFFD
IMSKKYANVPNVIYEICNEPNGYVNWRNSIKGYAERVIPVIKVNSPKALIIVGTATWSQSIEEPADDP
LKFDNLAYALHFYSGSHGAWLRERISYALSKGLTIFVSEWGVSQANGSGGTYPESTRDWLSFLDSKKI
SYVNWSLAPKAETSAILKTSARPDGNWKDEDLSSAGLMVKGFYVKKQD*
>SOL_1_270_cov_11.874740_N_27167
MKVKAFLFFLFFFVLFLDSNIALGELIYNGESAPFVASSSWDQNGSTLKESTKAPRSKPKHLRASITL
KNWWGAVAYVPKNWAALDISSSSSLVFWAKAGKNMDLLIQLFDLDKKNSAQIPIKLTYNYQKFSIPTS
SFTGVSLSKISAIIFAVGGRAGTSSYIVDIDDVAMTGSVTPPTPSPTPIPTPSPTPSPSPSPSPTPTP
SPTPIPTPTPIPSGTTMKTNGRFLYDACGQKMVLRGMNHMTCWTDWVGTARDGMPMFAEMAKTGANVV
RMSWITGENTTTAQLDAAITNAAANGLIPMPELHDYTCNWGTANITNVINFWTKPDMIAMIKKHQKYL
LINFANEMSAPSSTEYIKEYTRAVVAMRAAGIHVPIVFDSSSCGQDENMIKTAAPALIQADPDHNLIF
SLHIYWTDQNAARIAKAMTDVNALNIPMIIGEFSVKSVDCATPILWKEIIKQSQINQIGYLPWSWDHQ
NACADHSMTKDANQSFSTLWGWALELSVTDPYSIKNTSVRSQCF*
>SOL_1_270_cov_16.439319_N_27544
MKIIANIFLFTLFTFLLFVQTACGIEFAQVKDQKIIIDNKEFQFSGANQYYFFYKPQTMVDEVFEDAK
SLNMNVIRTWAFCDGGMHDGFCFQPEARKYDEPTFKKLDYVIYKAKKENIKLVLALANNWGDFGGINQ
YLSWTKKYNHDDFFRDEDMKSIYRDYVKYVLNRVNTYTGVAYKDEPTILMWELMNEPRCGDKYALYNW
IDEMSGYIKTIDTNHLVTTGSEGAIDSDPFEAHKSKNIDVVSFHLYPDWWGFNEQQANEYIVKHAQVA
KSLNKPVFLGEYGLKDRLKRNEIFTNWYSLANQNNVSGMLLWILSGKQMDGSLYPDYDGFTVWCPEFT
TMCDNLKKLSIEKIGAQLSYP*
>SOL_1_270_cov_98.701334_N_29922
MKRSCLILVFFLFLIPTTLLSYPVDDNGWLGVQNSQLVNQKNIPIQLKGMSSHGIQWYGKFVNYNAMK
ELRDNWGLSVFRIAMYLNEGGYLSRPDLKNKMIEGIDAAINLGIYVIIDWHILSERDPNVLKVPAIQF
FEEMATRYRDYPNVIYEIANEPNNTSWANNIRPYSIELYNRIRAIDPYHIIIVGTNAWSSAVEEAANN
PLPPYCYNVMYALHFYAGSHGSWYRDKITYAMSKGAAIFVTEWGTTNYSGNGGNNYGEAQNWMNFLAE
KKVSWTNWTISDHSESSAVLNPGANPNGGWTDANLKETGRFVKRAMLSK*
>SOL_1_270_cov_98.701334_N_29936

MRVLFSIILMFLFFSCGDNNHNNKNIDSKIPVDINTPAQQPAATNEVWRTAHGKIYKNNEIIQLRGIN
WFGFETPDLVVHGLWIGRSIESYLRQIKDLGFNSLRLPLSPEVFMSGYKSTQGYEKPLDNLNKLLKVA
NDLGIYVLLDLHTCSSKKGLVGSPLACEGYTDQSWFSTLKQMADLSLVHPNIFGIDLYNEPYKLTWKE
WRELSTAASKHVLGVNSNLLIFVEGTSGNGSNWGGNLVDAANELPDIKLSQLVFSPHAYGPSVADQPY
FKDPAFPANMPKIWDTHFGYLTSKGFVVALGEFGGRYVAQDVIWQDAFIDYLLKKGMTNFFYWSLNPN
SGDTGGILNDDWTTVNKDKYELIKKLF*
>SOL_1_270_cov_9.370175_N_30011
MFRAIIVFMFLFNPFGAIAMSQSVVEEMGQLSVRGSEIVNQRGEPVQLRGMSSHGLQWFGKLINEEAL
LDLRNNWGITVFRAAMYTVQGGYVENPKVKTKLLEIVDAAISLGIYVVIDWHILYDNNPQIHQGKAVE
FFSEMSKKYAGIPNVIYEICNEPNGNISWGGNIKPYAEAVIREIRKNDSKNIIIVGSGSWSQNIQDPA
NDPLRYQNIAYALHFYSGSHGNWLRERIDYARSKGIAIVATEWGTTDASGKGRIFIRESDQWLDFLDA
RKISWMNWSYSNAGESSAAITWDYKLTESGRYVKSRMLK*
>SOL_1_270_cov_8.023786_N_34350
MSIPQSHRRRVRLAFLLLALMVRFAFPGNSVESPDAFQQVGRLGRGVNILGYDPIWDNFAEGRFKERH
FKLIHDAGFQTVRINLHALQRMDAGNGYKLEDAWLSTLDWAVKNALANDLMVILDLHNYNDVAKDPEA
FKPRILAFWKQIAERFKDAPDSVLFEILNEPNGKLTPKLWSEFWSEALAIIRATNTARTVVIGPPFWN
GIGHLDKLALPADDRNIIVTVHYYEPFPFTHQSAPWSKETVHLSGVSWGTEAEKKRVEDDFGRVQKWS
LEHRRPILLGEFGAYDKGPLESRTRYTAHVARTAESLGWAWTYWQFDSDFIVYDIDRDRWVEPILGAL
IAEKE*
>SOL_1_270_cov_74.435899_N_35106
MSRFGRVSFMLLVLLLGTTFCYHHLDGKSLPTFSGANQYYLFYKPKVMVDDVFKSANELGLTVIRTWA
FCDGGFHDGFCFQPKAYEYHEPTFVQLDYALAKAKQSNIKLILSLVNNWGDFGGIPQYLAWYKLSSHD
DFYRDERVKDTYKAYVKHLLTRVNTITGVAYKDDPTILAWELGNELRSYDLPNFYLWTDEMARYIKSI
DPNHLLTTGSEGSISTDVYQTHKSDAIDFVSFHLYPDHWGFDQQRSNQYIRDHAQAARSLNKPVFMGE
FGFRDKSKRRDVFSQWYQIMKEEKVTGAAFWILSGKQADGSPYPDYDGFTVYYPESTDVIDVIKGYSN
HVKSQSLPIQESGGDIQNN*
>SOL_1_270_cov_162.827989_N_43331
MKIIMPFLLLLSSSAFSQFLYDGEAFPFSQAVKWDNNSSVLEESVKLPFSKPKHIRTSIVSVNYWGAA
GYVFDNWMPIDFSNNITLSFALRSSQKDTLLGIQLHDITGKTSSFARVETKYNYETIVVPLSMFADVD
LKNITAVIFSLSKPGTTTQIIDIDDIELFGKKKINPISGVMTTRDKTLLDACGKEVVLKGVNHMTVYT
DWIGTPRDGLPMYEEIAKTGANTVRIVWSYKENTTAKELDNAITNAAKNKLFPMVEVLDRTCQWSREA
FDEILAFWLKPEILSVLKKHEKYLLLNFANELGNNVTTLETYKSEYKRAVEKLRAAGVRSPIVIDAPG
CGQDEDTMLKAANYLINSDPLHNIVMSLHLWWVPQNDERITKLFTESARLNIPLVIGEFSGLAVDCKT
PTSVSHILKLANDHKVGWLPWSWDSANGCKEHSMTTDNTFNGLFGYGKEVAVTHSYSIKNTAVRSQCF
*
>SOL_1_270_cov_12.010587_N_47578
MRKFLKFLIFLSILFYGTAFSAVIYNGESVPFSAASTWDQNGSTLKQTNIVPHSKPKHLRANVIMKNW
WGAAAYVPSNWAAIDLSKASTISLWLKASAKIDVSFQLFDETKKTSAIYKLQAVPKYTKYAIPTSVLT
GVDLTKVTALVFGVSRSGSATYVIDIDDIETVGAAPVPTPTPTPTPTPIPTPIPTPTPSPTPIPTPTP
TPTPTPTPNNGTLDFGADGKLQLHPVGDSITDNPGWRKYIYDGLTKSGIKVDFIGTLTGQNPPFPEDQ
HDGHSGYSTRNVNDEIAGWFSQILKPELTIIMLGTNDVAWWIVEPVSDCVARLNLLIDKVRANAPKAT
IIVASIPPESSKILEPNKWDRANLVKEYNAGVAKIVADRKAKGELIHFVDIYSVLTLADLYDGIHPND
AGNVKVGQAFLTKIKSLISSSPTPTPTPTPTPIPTPTPTPPSPTPTPSPTPTPSPTPTDMINYRGIQG
ARPTNTQASGDVTFTIDLATKAPISPYIYGYQGFGNAWDKSLNFTMYRAGGNRWSAYNWENGISHAGS
DWRYQNDDYLCYGQGVTIANCATSGAVTKWFLDQTFNKNGSGLTSQAALVTIPLLDYVAFKSTDGDVC
PAANIDPAQPWECLTRSYLTTVFKPSKAKKGSAFSLTPDPNDGAVYQDEYVNWLRNYKTTVAPDKKIF
LELDNEWALWGSTHNRIFPLGDYCKHNPTPTSVLAKTLEYGAAFRAAFPDALIFGPVDYGWYSMASFQ
GFGQCDSTGYGMNFYDFWLKGIKGKNVVDVLDFHWYSEATGNVGRIISGNDTDNVEARLQAPRTLWDS
SYVEASWITDSLNKSAISLIPRVRKQIDANNPNMGIAITEYNHGGESHISGALAEADTLGIFGREGLF
AASFWPLTTPAQNYIYGAYKMYRNFDGAFSTFGNTAILSLTNSSSRSSVYASIFNQDPSKMIVVAINK
TAQPLAANITVAGASYSKMAVYQLTSSSANPVYVSTVDFAGKYTMPSYSVSTLVLANXXXXXXXXXXX
XXXXXXXXXXXXXXXXXXXXXXXYG*
>SOL_1_270_cov_7.222600_N_60440
MRQHVRMNQQKRRANSGIVIMAVALFILVNLSWSLSTEYLGNTILRIPSSGVIDGYIQYVYGDKIYEN

GTEVVWRGVGASYLLHAKDYIEAWNRHLPEIQEMGLNTVRLAFRFPDSNTGADGYKSSDTLNYTMLDN
VLGWLNLHGLKAILDCHNCLDMYGDFGSQKLINDWIALAGRYRFDPRIAAYELFNEPYWNTRDSSVRS
QEDVARAYQTLTNEIRKVDPEHIVIWEAQPHLPVLETISEYFDANMVFTLHKWYDKEDVGFRIWNVTT
FSFANIAGMVEMRSKVNKPFWLGEFGSYYPWNSSNPEWLLTNETLQRCEEQILGWSLWMHESTGEKTW
DEYLQLFPLESDQNSVTRKPWILPLPNMLDSIISQRHSDIFEPCRIELRHNNDQVTFKGSNLVILVVT
SHLLSDGSSETVGEQELTLNAELTITNEENTVNHPGDWKTVIYILNN*
>SOL_1_270_cov_5.938352_N_63422
MKEIIKHLKITFLEIATVSLLLILASQSVFSAMVGSSIIHSSGQIVTLSPLHVEGSALKDVNNNTIYL
RGCGKIHWDDDPTGWWWNTSWDSNYCVWDESAVRYHLKTMKSWGMNLVRFHTVAEWWLTDPIVFKDEN
GVTRWTGSYRNNLKRAFQIAQEEGMYVIMDLFAIKNGYYYYSNQNTQPPGCIPFTPYVRDPGEIAIFP
DGKQSFVNYWANVANELKTYPNVIFELYNEPNNMGFENQTTMVRADWFDACQKAITAIRNTGSENIIE
VQWWWGSAPGTLYDARLMWVEDYPLTDPENNILYSTHLYRCFYNPTWGPCYNYTECLNGMINCQFEYV
VKTLNKPLIIGEMGVNMFYSGTDLQHELDWARNVLSICNQWGISYSAWDWTITDQWHLISSNGLGDGP
SAWGQVLINAIAEGGTRANP*
>SOL_1_270_cov_6.809161_N_96733
MDGVVNVFKCNWLYLDFAKVRLVIIMITNLQINRKDKTMKNLHRNIIKIVFLSSAIMASTAYGYYTTK
DQDIIDRKTGEKVLLQGFGLGCWLLPEGYMWGLRTLDRPRQLEKAIVDLIGPQDANEFWRLYHDNFVT
EGDIKAMKAWGVNSVRIALLASMLQPRDGQPAAAPYLYSEEGFSYLDNLVRWCDKYKVGVIWDMHGAP
GGQNAENISDSDGAARLWTEKGKYWPLCIDLWYKIAERYRNEDCIIGYDLLNEPLLRRYEGIDVKLLR
QFYVELTEKIRTVDPNGIIFIEGDDWAQNFSMLEPIDWDKHLVIAFHSYPPTSSQDGLKKWDDLRKKY
NIPLWHGETGEQGPPFIVNTVSTEFLNSANVGWNWWTHKKFDRLTQPWCCPKTEGFQKIIDYWKGTGP
KPSKEWAKRWLFDQARKTHSDQCEFTPDLVRSLVPLNPDSYLASRGIIAPKIIQQPKDVGLEVGDCAT
LIVGAGGYPVNYQWKKNGKILPGENNFKLRIQNPSLKDNKAEYTVTVSNKKGSDTSRKVTLTVKPYSG
PIIAKASVPVEIDGVVDEVWKNAELLPLANVALGEKPSAENLSGAFKILRDQTNLYLLVQVTDDLKMH
TAEEGYENDGIEIYIDYDNSKSDRYDSDDFMFRYVWSESEVLSVIGNPGPGVKGAQKNLDKGYIMEIA
IPWKAIGGTPKEGQYIGIDVHVNDNDNVRRDCKITWKAKRDISHQTPSVFGTMKLSE*
>SOL_1_270_cov_6.550195_N_97369
MGADKSENPGQTGNPGLYSPAMRTPLAHLVALVLLAAPAACFGAEPSMEAVRVSPDHKGFVLHPSGRP
YIPWGHNYGSVDILDRMAKDPARVERDFEDMKAAGTTVARIHPEMPLLVTGPDKADPQAIDRLSRLLA
IAEKTGIYLDVTGLACYKINNRLAWYDAMDEEARWKTQEFFWETLARTCAPSPAVFCYCLVNEPAAAA
KKTDGWYVGKMGDVEFCQRLTLDAGTRSGDDIFREWTKRMVAAIRKHDKERLITLGMLPFPGAYKAAA
EQLDFVSPHLYPKSKKVDEEIALLKKFDWGKPLVIEETFPLSCGADDERDFLLKSRGIAAGWIGHWPD
EPPAQLAELRRTGKATIHSAIWLSWVELFKELGPQMTGRK*
>SOL_1_270_cov_5.478909_N_101904
MVDEIMETAARMNLNVIRTWGFCDGIKKDGYVFQPSPRVYNENTFKKMDYIIYKASQYNIKLIIPFVN
NWDDMGGMNQYVRWAKGSNHDDFYADPWIRQVYKDYVKHFLTRVNSITGVAYKDDPTILMWELANEPR
RQTNNGVEFQWWVDEMGLFIKSIDPNHLLAIGDEGFLANKPDGDWMHNGSQGINFIANNQSKYIDVAS
FHMYPDPWGLNEKQAKEWIEEHIDIAKNILNKPVFMGEFGIADKGKRDRVYSEWYDLAIARGLPGIVF
WLLSGHQDDGSLYPDYDGFTVYTHENTSVVIRNASMRFLDRSLDNLD*
>SOL_1_270_cov_12.317342_N_104836
MSSASGFNPEAKYAVTMWEFSWLVRRTGDEAEYADWDKVLDELAERGYNCIRLDAFPHLVAKGPDGQI
VEQFTILPQPDSFMWGNHKPVQVEPRSALVEFIGKAADRGIYVGLSSWYNLDTRNRVQMIQSPKDYAR
IWLETLDLLSEAGLHDRIVWVDICNEFPVRRWAPGPYTDIFKSKRVGDLWMTLKLSRKWDEGVKQRIK
NYFDGAITPLREKYPDLKYTFSFQFMGSRQMQAIDVGTFDLAEVHIWVSDYMKWMFRTGQVLLHIGFP
YPRSLRIHVKRMANLYPKYRDKYASMLEARIDFWAEWGKKNNIALFTTEAWGPINYDEVTPGGTGGEW
DWVKDIAEHGVRMASEREWQGICTSNFCQPHFEGMWADVGWHKRMTDLILRR*
>SOL_1_270_cov_6.582913_N_106433
MAADFRLLHVDGPRIVNGRHEDLRLRGFCLGGWMNMENFILGYPGHESGFRAAMARVLGEDKARFFFE
RFVHYFIGEDDLCFLKRSGCNVIRIPLNYRHFESDGIPFEYKEEGFALLDRAVGWARNQGLYVILDLH
AVQGWQNRGWHCDNGCRTAHFFGQRVFEDRAVALWEELAGRYRSEPFLAGYNLMNEPDADEVKWLNHF
YRRATDAIRAIDPNHIIFLEGNRYSQQFDELDPPFDKDTVYSSHNYAVPATEEMAYPGEFKSESYDRS
RLEREYDERTAYMMRHGVPNWVGEFGAIFGGETSDASRLQVLADQIEMIETRGHHWSIWTYKDIGKMG
LVCLDPSSEWMRRTRPVREAKRLLRCDHWIEKQPGSIDPLLPQIGAAARKALPDLPLDAQQLEAELSG
AVCETTLAQALLPAFAEQFRGMTEEEIDRMMQSFAFVNCVPRSELVRLLDRRLGRHREAATA*

>SOL_1_270_cov_4.680876_N_167000
MIRTAGPWFKDEHGRTLLLRGVNLGGSTKVPFTPDGATYRSEGFFNHRQVSFVGRPFPLDQADEHFER
LRAWGFDFLRFLVTWEAIEHAGPGIHDTEYLDYVRKVVQKAGEHGISLFIDPHQDVWSRFSGGDGAPG
WTLEAVGLDMARFGQTGAAVVHQVRGDPFPRMIWPSNSLKLAAATMFSLFFGGNDFAPQTEIEGVPVQ
EYLQGHYIESIRQLAIRLKGLSNVVGYDTLNEPLQGYIGWEDLNGPGGMLKIGECPSPFQSMLLGAGI
PQEVGEWSMGIRGARRVRSALVNRDRARAWLPGFDCLWKRHGVWDLDPEGRPRLLRPDYFVRVRGRQV
DFAADYYRPFANRYAAAIRTVAPEAIIFLEGEPNREGPKWGPDDAPNIAYAPHWYDGFVLFMKSFSPF
LAADMQGRLTFGARAIRRSFAAELAMYPQAAAERLGDAPTLIGEFGIAFDMNGKKAYRTGDFGAQIQA
LHRSYRAMEDNLLSCTLWNYTADNTNRRGDQWNDEDLSLFSRDQQTDPKDIHSGGRALRAAIRPYPKK
TAGAPIRMAFDWRSRVFDFEFRHDPRLEAPTEIFVPSFQYPSGCVIKVSDGTYELNGEAQILTYRHSA
AQDLHRIVLRPR*
>SOL_1_270_cov_3.809077_N_174848
MDKRSLQIGINLGGWISQYSAYSHRHFKTFITADDIKRIADWGVDHIRLPLDYPVLEDDRKPGVYKES
GFEYIESCLNWCEENGLRLILDLHKAPGYSFDALNESSLFGSPALQDRFLGLWQAMAKRFADRMDDTL
AFEMLNEVVLPDSGSWNLLIKRVAACIRSLDPQRLIVIGGNHYNAPDELANLEVLDDPNILYTFHFYA
PLTVTHQKAPWIPALAQFNQQVEYPGQAAGLEAFLEANPELRNTLGTEVGRQFDVGYLESVLQPALEF
AQRFGQPVYCGELGVYERAPMTTRLNWTRDVIELLNQRRIGYAVWTYKNLDFGLVDKDGRIVSQELIE
IVSHR*
>SOL_1_270_cov_8.393896_N_190007
MFKIFCVLCFFFSGIALGDMLPLYNGETVPYSKGVAWDKNKSKICESIKNPYSPTKHIRAQIKNRNWW
GGVGYFQNNYKPTDMSSFKFLSFYAKSNKKVDLIFELFDSKEVVSTQIVFQVDKEYKKYLVPLELMQK
VDLKNVIALVFATSQKKNADFIVDIDDIELNTDMPFPPVPTPDPTPIPTPTPPTPGTDTIRIIGKDIQ
DACGQKIVMRGVNQMTCYTDWIDTPRDGLPMFKEIAKSGSNAVRIVWTYGEGLTIAQLDKAVQNAKDN
ALFPIIEIVDQTGKWSREAFDTVIKYWTAPDMVSIITKHQKYLIVNFANEMGTGAVTKEQWVEEYSRA
VIAMRKSGIKVPIMIDTSNWGQDETFILASGKKIIEADPIHNVVFSLHVWWTDQNAARIETTMKNINA
AGIPFIVGEFSSVSVDCKTPIKYKELLKYAQENQIGWLAWSWDHENECPTHAMTRDKEQTFASLWGWG
LEIMTTDINSAKNTSVRSTCF*
>SOL_1_270_cov_4.849926_N_195021
MKYLFLLLLVFAGTAKGAETILYNGETVKFNTSSAWDTNGSSLVPSSAYPCSSPSHLRATLKVKNWWG
GAAYVPSGWSNVDMSKATYLSLSVRSAQAGKVSISLFDAGKKSSARLSIPLSPSCKNVQIPMSNFTGV
DLSKVQAIVFSVSVAKSPTYVVDIDDIKMITPTPTPTPTPTPVPVPTPTPTPVPVPTPTPVPVPVPTP
APSSSLTMKTNGRFLYDACGQKMVLRGVNHMTCWTDWVGEPRDGLPMFAEIAKTGANVVRIVWITTEG
TTTAQLDAAITNAVNNGLIPMVDNQDKTCKWSTADINAVLSWWTKPDMLALIKKHEKHLLLNFANEMS
APDLTSYVSEYSRVLVALRAAGIHVPVVLDSSGCGQKEQIIMDAGPALIKADPDHNVVMSLHIYWTDQ
NAARIAKAMTDSVALNIPMIIGEFSAVSVDCSTPILWKEIIKQAQINQIGYLPWSWDNQNSCATHAMT
KDNNMSFSTLWGWALELAVTDPNSIKNTSVRSSCLK*
>SOL_1_270_cov_4.982634_N_210386
MCESTXXXXCALPISFQYGNADENNKLSETWFQILDWAIEQALSNNLTVILDFHEFNAMARDPLGKKA
RFLAIWKQIAERYKDYPDEVIFEILNEPNRELTPELWNGFLGEAXXXX*
>SOL_1_270_cov_3.460439_N_212561
MSQNPLHIDGCWFKDEQGRVVILRGVNVAGNSKVPPFIPFTDAALLDPLKEWGMNVIRLVLIWEAIEP
EPGKYNEGYIDAMETLVNAAGERGIYVILDMHQDMFSRYVNGGCGDGAPSWAIDPSIPQDEPSNDERC
IDWINGLNDKNVLRAFDSLYANANSIRDHYISMWAHIARRFGDHPAVIGYDLMNEPIGDEVSQLALLY
EDAGAAIRKVDPDGILFVEPSILTSFGAIYSQLPPLSLGNYAYAPHFYSASLLVTDIFSTSEADKSFA
DFNSKVKEFXXXXILGESRCSWENLECILKRLKSRNTSLTYIEDSMIVSMGELNGITAQDGAQWLWMA
GTGRITQSLMTRVIYAGTLRLEAMLSVLPGYHKSWRSAITESTWSGRTSRKSLQPPCCTFP*
>SOL_1_270_cov_4.709763_N_232553
MNRTTNHQTVLKLFSLFSFSFFLLTFTFVDTQAATLPELKIPDGFGVNIHFTGEPKDLDLIRDGGFKF
IRMDFSWSSIEREKGVYNFELTGYDTLTAGCIKRGIRILYILDYSNRLYETEQSVRTDAGRTAFAAFA
EASAKRYAGKNILWEIWNEPNIKQFWSPQPGVDDYCKLVESAASKIKQADPTGLVVAPATSTIPFDWL
ESCFKKGLLNHIDALTVHPYRPKNPETVIKDYARLRELITRYATQGKNVPIFSGEWGYSNVNWDNTRL
SNVQQAEYLARMFLINLYQNVPVSIWYDWKNDGPDPNEREHNFGTVERDLSHKAAYAAALTITKNLSG
YSIKERLDTGSENDFVFKLSNGKNQAIAFWTTGQEHEISLPVSGSAKLVNMVGCTSNLTLTDNYKAKI
SPSPQYLILEPQ*

>SOL_1_270_cov_3.792257_N_271664
MYRAPGFAAWKAFYYNRADTEKPEKYAGPWNHKADHRGPNQATEARVYRWVKNPHYDPVGTEIADPAP
HDVSGSWWDAGNFDKYMGNTTLCHNELLLGVELLGAAPKDGDLAIPESGNRLPDVLDEIRCATAWLIR
MADSTGAAWGRVYEKTGCPPEADTSPVMLTQQTSGATMNRAAALAWASTVWQERKLDPAFAKQCMDES
MKSWQLLQTKPHPWPPDAKDPKKPAYTGEWFFVDFEKCRALAAACYFRATGKPEYEKIVQESASKWSI
PPGENVEVWPTIWVYVHTPGADAATVKKLRDMLSGAADGVVKQTGENRGYAAGVRGYWWGSNRAIGQA
GLNCLVAAELTEDPAARARYLAAAEEYVHYLNGRNPIGLCFWSNMKALGAENSVMVMFHAWVGNFSAK
EGQKYIGEGEGKIGPFPGMVVGGVNGGMKKYINNLDWRQNPWEFNEPCITYQSSCASLLMYFGLKGK*
>SOL_1_270_cov_4.386578_N_271805
MDSLILDIGSERMIASGKPAEKGLQFPRQGRVGREMSGKTAKHGYTARDGWFLDPLGRHTLLRGVNLS
GSTKVPYSPNGATHLGVDFQNWADVSFAGRPFPLAEADEHLSRIAHWGFNTLRLLVTWEAIEHAGPGR
YDEAYLDYVREVTKKAAEHGLLVFIDPHQDLWSRWAGGDGAPFWCFDWAGLRPERFVEAQAVELNAVD
WLNNYNRVPTATMWTLFFAGDTFCPELAGVQGRLQDHYVGALCALAERLADLDNVLGYDTLNEPSGGY
IGRGEDLLQAQATFTATGAPQPFSALEHLAAADGQTVRRPDGEVLNPAGVSIWRDGCPWRRAGVWDID
KEGNPLLLSASYFREVKGEPVTAWGFMVSFIRRLRDALRRVHPGCFIFIEGSPFELPTYWDDPDPLVC
NARHWYDATTFASRKFRPDSYQSLSGKTVSGVQEIAEEFTGQMGYLQLFSREQMGNPPMLIGEFGIPY
DMNDGEAYETGDYSLQEIALDANYRALEALLLNSTQWNYTTDNSHAHGDQWNHEDLSIWSRDDQKDPG
DPDSGGRAVRAFCRPYVRHAAGRPTRMAFDPATATFELEIEGDAKVTAPTLVYVPRLHYPDGVEVSVS
>SOL_1_270_cov_5.380976_N_281674
MGFADPCSVFLSPGDASSEPRALASTAFKIRGREPPLSITPEPRLTVNRAACAALAVFAVLILGLAAV
FVLTIMIGLLPMPGRPEATPTAPRPSPTSHASVETPPVDTPVAPAVQTPSPTPPGTGFDYGIQVHLFH
LDAGYVAELVNNLQFRWIKHQIEWKEFEPTKGQYLWEPIDQIVNPAHSSGLNVLLSVVKAPDWARGGH
TTEDGPPVDYSHYGDFMAALAQRYCGKVQAYEVWNEQNLRREWNTARPLSAAEYVDLLRVAHERIKAA
CPTAIVVSGGPTPVGYTSATAIDDFEYLRQMYEAGLPNYCDAVGVHPSGFNNPPDWHYPPAYSEAGDS
ESFRGNRQFYFLN
>SOL_1_270_cov_3.616499_N_304562
MMRAICAMIVLLAISAAAFGEAALAANAGEVVFAAGFEEPKAIDGWSVRSGAGSVTLDAGSESAKALC
VTAAADAKGAATVERPLPLDRVRGCKLLLSAMVRAEGVSAKPQSWNGIKFMMPMTAGGSKLWPQAAIE
VGTFGWRRVVWQAYVPDDATQAVLVLGLENVSGRVWFDDVKITVRRVLPPLVARAAAGPIYRGHTLPR
LRGAMVSTNATEEDLRVLGQEWKANLIRWQLCGFKPRMDTGDTAAYETMLAEHLKKLDAILPACEKYG
LMVVVDLHTGPGQWAESGRGLFTSAACQKRFVEIWREIARRYKGAKAVWGYDLVNEPHESPFDPELSD
WQELAERAAKAIRAIDPERTIIVEP
>SOL_1_270_cov_2.998284_N_310345
MPFEIHRGTNISHWLSQSKTRGQERRTWFTRTDVQRLAGLGLDHLRIPIDEEQMWNEKGVREQEAFDL
LDAALDWCRDANLRAVVDLHILRTHYFITAEEPRLFADPAEEVRFAGLWTDLSAHLRRRPNDGVAYEL
MNEAVAADPAVRLAAAQKTGRQTWEAIFRAAEGSTKALRDAIAAVAFSPTVQAD
>SOL_1_270_cov_3.426061_N_323712
MMTLWASQSQAEILYDGETATYSTGQAWDKNGSTIMPSKAAPYSSPKHIRAKIVVKNWWGSVAYMPKN
FQPIDISSQRSFSVYLKAAKPANIILQLFDANKQTSAKYQVPVETTYSQYIIPLKAFSGVDLAKITAI
VFAASQKAPISHTIDIDNIEALGAAVPEPSPTPSPAGTAMKTNGRFLYDACGQKMVLRGVNQMTCWTD
WVGTPRDGMPMFAEIAKTGANVVRMSWIRDAGAGVPPISIAQLDAAITNVVANKMVPMPELHDYTCGW
SAANITNVISFWTQPEMIALIKKHQKYLLLNFANEMSAPSTAEYVKEYSRALVALRAAGIHVPIVFDA
SGCGQDENSILAGGPLLIEADPDHNVVMSLHIYWMDQNAARIAKVMADATAKNIPLIIGEFASVSVDC
TTPILYKEIIKQAQINEVGYLSWSWDNQNACAAHSMTKDSNQSFSTLWGWALELAVTDPGSIKNTSVK
SNCF*
>SOL_1_270_cov_3.389274_N_332979
FHCYEPFKFTHQTAGWAGDSVRGLKNAPYPSSPEAVAKVLAGLPDEKARQNMTQYGKENWNAAKLDAL
IARAAAWGKKNGVPLTCNEFGVYRNALAADRCRCIADVRTACEKYNIGWAMWDYAGGFAVATGKPGSR
VPDPETVKALGLTMPK*
>SOL_1_270_cov_3.943680_N_342273
MKTKPLLTLAGLLLVPLTALAAEPSSNDAPGRWPAAKAWAWYRKQPWIVGFNYVPSTAANTTEFWSGE
TFDENTIDRELGWGARLGFSSCRVFVQYLVWKHDSDGLRKRLNRFLSLAEKQGLSTTLVLFDDCAFGD
PPQTEPYLGKQREPIPGMIAPSWTPSPGLKAVTDQSAWPDLERYIEDVVGACGQDKRVLMWDLYNEPS
KSRPLVEAAF

>SOL_1_270_cov_4.800954_N_352373
RPIAVPNVVYSVHMYNPGSFTHQGVFGEWVKKIPYPGETEGKMWDKAALEASLKPVIDFQKAFGVHIY
VGEFSAIRWAPDGSACRYLKDLIDIFESRGWDWTYHAFREWQGWSAEHGPDKADAKPAAEPTDREKLL
REWYAKNQKPQG*
>SOL_1_270_cov_3.599860_N_358474
MRFFCPFLIAMLLFINPLFAQTVSEAGRLKVQGCYLLDKSGYAVQLKGMSSHGLQWFGKFANINAMRE
LKEKWDQRVFRAAMYTEEGGYLANRNLKNKVHEIVRAAIELDIYVIIDWHILSDRNPMWHIYEAKEFF
DEMSRTYAGYPNVFYEIANEPNGGDVRWDNAIKPYAKEVVSVIRKNDPEGIIIVGSGTWSQDVQDPAN
DPILEKNIMYSLHFYAGSHGQYLRDRIPAAMSKGIAIFVTEWGAMDSSGRGGLDYGSTDAWMKLLEKY
YVGWTAWSLSDSQDSHAVLRPGSSPNGGWGEGNLSANGKLVKRYMLQ*
>SOL_1_270_cov_5.120311_N_361832
MKFSNLLFLFKPFIPIFTFLFISFGTNNLNARTEKASIRKPKGFLRTNGYDMVDEDGNKVYLKGVGLG
NWLLPEGYMWILGKYADRPRRIEALVYDLIGKEKGKEFWENYRKYYITEADIKRISELGFNSVRPALN
ARWLLDEDREELVYNEETFKLLDNLIKWCKKYDVYVILDMHGAPGSQTGE
>SOL_1_270_cov_4.514039_N_366335
YTRAITAMRLAGIRTPIVIDSSECGQSETMIMAVAKDLIAADPDHNLVFSLHIYWMDQNAARITKAIT
DSVALNIPMIIGEFAHKAVDCTTPILYKEIIKQSQSNQIGYLPWSWDKENGCAEHSMTKDNMFSSLWG
WALEVAITDPNSIKNTSVQSQCF*
>SOL_1_270_cov_4.289432_N_392078
IPSLFWHTATVSDLVGEPLDQLGNPGSKSIALIRKYTEDVVRRYENSPAIWGWEFGNEYNLGADLPNA
ATHRPPAVPKLGTPAERTERDELKFAHLRTAFVAFAETVRKFDSQRVIFSGNAIPRASAWHNFHEKSW
KADTEAQFGEIFLRDNPDPMNSVTIHLYREDKDKYPGGAASIDEAVALATRCAAKAGKPLFIGEFGAA
RAAGSREAQQALFEEFLGAIEKHRVPLAAFWVFDLAGQDKDWNVTFQNDRAFMIELVSRLNVRLRAEK
QEKP*
>SOL_1_270_cov_3.067220_N_407469
YGDQIHAWQIWGEPNVSTYFRGPSPATYASLLKQAYLAAKYADPTALVILGGLANDASAAFPGVTFIP
PDAFLQGIYDYGGGQYFDAAARHPYATPNQDGFHYLDQQLSNFRQVMVLNGDSDKPIWITENGWPSYS
QWTEAMQAQWLGDSYRYMAGLDYIGPIFWYNLTDKGTDPNEWEHNTGLVRRDWSPKPVYYAYQEFIAS
H*
>SOL_1_270_cov_2.830268_N_409837
MPEEKQAKSIDQATLEMIAKADMFAVISFRTGPGRSEFTFYWGTQGDWFTEDYYNDTVWEDEPAQEAW
VAMWRYTAERYKGNPVVVGYDLMVEPNSNDVWFNEWDPETFYAQHGSSSYDWNQLAQRISDGIRQVDA
QTPILIGGDAYSAINWLPYVKVTGGARTVYLVHQYSPVQYTAQEPGEEGYTYPGEFDIDGNGSTETFN
RNWLEKTLFSVIEGFTSEHAVPVAANEYGVRRWAPGASDYLRDLMDLFESHHMNSALWEWSTSWPERA
TNYDDYNFLHGLDPDNHEDVDNAL
>SOL_1_270_cov_2.162045_N_421173
VVVLVLGLAVGSPCAWAEEGKSPASAKAPPKIRVDRERHGFADAAGKPFVPFGVNYYRPGTGWAPQLW
KQFDAEATRRDFALLRKHGANVVRVFISFGSFYTEAGKLDADGLAKFDKLLDLADAAGLYVHPTGPDA
WEGMPPWTRELSVFTNDTNEPCMKALEDYWRLFAARYRDRSTIWAYDLRNEPKLAWDTPYMRTQWEAW
LRERKQDPAAVPDPKAAPPAACLAEYQRFRESIAEKWVARQAKAIHDADPEALVTVGLLQWSVPAQRI
KVNDYAAFRPSVIARHLDFMELHFYPLVSGAYTYGGAAAEAANLAVLESMARECARPGLPLVIAEFGW
YGGGPLDPGGTPATEEQQAEWCRHLVEVTAPMACGWLNWGMYDHPQAKDVSRLTGLFTVDGA
>SOL_1_270_cov_7.944395_N_426875
PTPTPTPTTGTLDFGTDGKLQIHTIGDSITENPGWRTYIYNEMTKLGYKTDFIGSVTDQWPQSTESQH
DGHGGWSTSNVIDAIGGWFTTILKPELTIIMIGTNDVAWWSAETPADVVVRLTTIIDKVRANSPNGYI
FVASIPPESSKVIDIVKIDRAQLTINYNKGIAALVAKRKAMGEKIQFVDIWSSLIVSDLVDGIHPSDA
GNIKVGTTFLNAIKTLLSNVPAPTPTPTPTPTPTPTPTSNPIPTGTTMKTDGRFLYDACGQKMVLRGV
NHMTCWTDWEGTPRDGIPMFGEIAKTGANVVRIVWIYTEGTTVEELDRAITNAAANKLIPMIEIHNQT
CNWSTAAVTEVLNWWLKPEVVALIKKHQKYLLVNFANEMGNGGTTATSFKNEYTR
>SOL_1_270_cov_2.024479_N_435803
MHIDGPCFKDESGRTLLLRGVNLGGSSKVPYTPNGATYIREGFFDHRQVSFIGRPFPLEEADEHFARL
RKWGLTFLRFLVTWEAVEHAGPGVYDQEYLDYLHAVIAKAAQHGIRLFIDPHQDMWSRFSGGDGAPGW
TFEAAGMDVTKFQPTGAAFLHPVHGDPLPRMIWPXXXXGRSP*
>SOL_1_270_cov_3.720779_N_443434

LMSDARIPAVRVDFEWADIELHKGTYRWGEYDALVSILVSEGVNVVPILTYSPQWATDGDSKFSSQTS
PDDFGAFVGALARRYRDRIDVYQIWQEANTSLYFDPPDPHRYSELLNIAYLNVKYYDPGAVVVMAGLA
NDASAAYPGTVFIPPEDFLRALYENEALFDVVARHPFTHPLETLDTLRQRMQTIRQTMSQHGDNYSQL
WVTEYTASTNLGHPGGLSEEEQASWLSQSLRVLLNEHLADKVFWYNLRDTVVDSDPFFAYSGLVHHDL
TPKPAWYSFCELVNEYP*
>SOL_1_270_cov_1.700888_N_465842
MTALAVILLSLFAFFILNFLKKLSYSPDKSNFTLFKDSDGIKYIYHGVNICNYSKHSPDLLPWMTKED
YGRLNKWGFNLVRFLVFWEAIEPEKGKYDTAYLSKLTEHLNILDSLGIKVIIDVHQDLFSKKYSGNGF
PLWAAQDGGKPFKKVKGPWLLNYFQAAPRACCNNFWKNKELQNRYIAMIDYLITYLQGQRNVIGIDVM
NESFPPLMKLLSFEKKVLTPFYCDILMKIKNMTKCLFVEPAIYCSTGFPSNIGKVTLRNSAGYMPHYY
DVFCHENKPYGFLNKILMKRSLAIKARESQLINSPFLIGEFGVSEKVKNFKKYIKDFCGIMDSYCGSW
CWYCYDKTTF
>SOL_1_270_cov_4.400599_N_470147
VRRPAAHRLRGAYVGSRRAPARRRAPPAPLRAVGPRPPGAGPEGRPMRIDGACRATCRGLIALVLGLL
LAGATGGPVPAERSEAGLPGSAAPPPEAAAPAVKAGDLVFAADFEDADVLGAWSGSPRTDAGRQGGRS
VVVTCADGASAAAVTVPLPAAHLRGCKIIFSAYVKAEKVSDRPQPWNGVKFMAPIDSPSGRLWPQGAI
PVGTFGWRRVVWQTFVPEDARAMTLVLGLERVTGTAWFDDVRVVVRRPPIVRRPRPAAAGPVYTGRDL
PRLRGTMVSCDPTREDLRVLGRLWNANLIRWQLLGWQPPAERTALDAYDDFLESHLKRLDAALPWCEE
YGLRVVVDLHA
>SOL_1_270_cov_2.569815_N_481148
LDDLATEVVVADVAFNPPRTWLLQQAAQRGMSTIDGIEVFIEQGAINFRLWTGLEPERTIILGSNRWN
SAITFYELDVPADDANIILTFHFYNPMLITHYRAPWTPVGRYEGPVHYPGQPIKPEDWGGLAPDLKAL
LGAQNVFYNGSVMERDLAPALAVQRRTHLVLYCGEFGVHHAAPDPVRIAWYRDFRGVLARHGIAWANW
DYKGSFGLFDAKGNPTAVVEGLLK*
>SOL_1_270_cov_2.480042_N_489951
MIRIDGPWFKDEHGRTLILRGVNLGGSSKVPFRPNGATYIREGFYDHRNVSFVGRPFPLEEASKHFCR
LRAWGLTFLRFLVTWEAVEHAGPGIYDQEYLDYLYAVVRKAGEHGIHLFIDPHQDVWSRMSGGDGAPG
WTFEAIGMDVTRFSETGAAIVHAMHGDPFPRMIWVSNYTKLAAATMFTLFFGGNDFAPRTRVNGEPVQ
EFLQRHYMNAMKQVVQRLQGLPAVVGYDTMTEPSAGYI
>SOL_1_3_cov_39.558537_N_5061
MKKILSLLLLMFALQSYGESFVKASGTNLVQDGKRYIVKGTNQYYLFYKSKKMIDEVIDDAAAMGLNT
LRTWGFCEGELKDGYCFQPSPRAYHEPTFQKMDYIIYKASQKGIKLIIPLVNNWDAFGGMNQYVSWSP
TAPKEHDAFYTDAWTRQLYKDYASYFLNRVNTITGVRYKDDPTIMMWELTNEMRAQSDYSGSKIQDWV
IEMSAWIKKIDSNHLLSTGMEGFGRNEGKDWLHDNSQGTRYIENHKVKDIDVVAFHLYPDHWGISKEY
ALYWIASSIKLAHDQVGKPVICGEFGFQNRYERAAVYKDWYSMHKAKNGDGMMFWLLSGRQDDGSLYP
DYDGFTVYFPEDKEVVQVIKEGG*
